# Delayed reward information is underweighted in reinforcement learning with dispersed feedback

**DOI:** 10.1371/journal.pcbi.1014459

**Published:** 2026-06-29

**Authors:** Miruna Cotet, David Poensgen, Ian Krajbich

**Affiliations:** 1 Department of Psychology, The Ohio State University, Columbus, Ohio, United States of America; 2 Complexity Science Hub, Vienna, Austria; 3 Faculty of Economics and Business, Goethe University Frankfurt, Frankfurt, Germany; 4 Department of Psychology, University of California, Los Angeles, California, United States of America; 5 Department of Economics, The Ohio State University, Columbus, Ohio, United States of America; Peking University, CHINA

## Abstract

Learning is fundamental to adaptive behavior. In the typical learning task, each action is associated with only one outcome, which could be immediate or delayed. However, actions often have multiple consequences that unfold over time. Here, we used behavioral and eye-tracking experiments to study how people learn when their choices yield both immediate and delayed reward information. Importantly, the rewards themselves were all delivered at the end of the study so there was no reason to weight immediate and delayed reward information differently. Instead, we found that our subjects overweighted immediate reward information. Moreover, this bias increased over the course of the experiment and was still present when learning from others’ choices. The gaze data reveal mixed evidence that subjects looked more at immediate vs. delayed feedback, and across subjects, the relative dwell proportion did not predict the behavioral bias. Our results indicate that people prioritize not just immediate rewards, but immediate reward information. Unlike temporal discounting, this form of impatience is a clear mistake and leads to objectively worse outcomes.

## Author summary

Decisions often produce several pieces of feedback. Some occur right away, others occur later. Although these signals can be equally useful, people may not treat them that way. In our study, we asked whether humans learn differently from information that arrives immediately versus information that arrives after a delay, even when both are equally valuable. Using a combination of behavioral experiments and eye-tracking, we found that people consistently placed too much emphasis on the immediate feedback. This tendency grew stronger over time and even appeared when participants were learning by watching others’ choices. Our findings show that people aren’t just drawn to immediate rewards—they are also overly affected by immediate information about those rewards. Unlike classic impatience, where immediate rewards are often genuinely better, immediate information provides no benefit in our setting and overweighting it leads to poor decisions. Our study reveals a systematic and costly quirk in how people learn, and offers an explanation for why people often seem so shortsighted.

## Introduction

Learning is fundamental to adaptive behavior [1–3]. To thrive, organisms must learn what things to pursue and what things to avoid. Reinforcement learning (RL) theory [4] is the dominant framework for understanding how organisms do this [5]. In the RL framework, learning involves updating reward expectations based on the difference between experienced rewards and prior expectations. With enough experience, one’s reward expectation should converge to the true expected value [6,7].

In the typical RL task, each action is associated with only one reward, which could be immediate or delayed. However, outside the lab, actions often have multiple consequences that unfold over time [8]. In such cases it may be difficult for the organism to learn properly. For instance, credit-assignment is a well-known problem in RL where organisms sometimes misattribute rewards to the wrong actions [9,10]. This problem can occur when there are more actions than outcomes or in complex environments where there is ambiguity about which action(s) led to the reward. What remains unclear is whether people learn appropriately when there is no such ambiguity but there are more outcomes than actions, i.e., when a single action leads to both immediate and delayed rewards.

It is well known that organisms often discount the value of delayed rewards, meaning that they often prefer smaller immediate rewards over larger delayed rewards. This phenomenon is referred to as time discounting or delay discounting and usually thought of as a preference [11–15]. However, it is possible that at least some part of delay discounting is due to inaccurate learning. A difference in the processing of delayed relative to immediate feedback could generate discounting-like behavior that is based on learning frictions rather than preference.

There are many reasons why immediate and delayed reward feedback might have different effects on behavior. One reason is limited attention – people may prioritize attending to one kind of feedback over the other. Biased attention can lead to biased choices [16,17].

A second reason is agency – people learn better from things they choose compared to things they don’t choose [18–21]. Delayed feedback may feel less agentic than immediate feedback. If agency-related boosts in reward prediction error only apply to the most recent choice, then one would expect a preference for options that yield larger immediate rewards.

A third reason is memory accessibility – people may find it easier to retrieve immediate feedback than delayed feedback at the time of choice, leading to a stronger influence of immediate vs. delayed feedback on choice. We know that people put more decision weight on information that is presented earlier [22], considered earlier [23,24], or retrieved from memory earlier [25,26]. If immediate feedback comes to mind more quickly than delayed feedback, for instance because of the speed of striatal associations, then this could produce a bias towards overweighting immediate feedback.

A fourth reason is an asymmetry in reinforcement learning – people may learn from delayed feedback at a slower rate than from immediate feedback. This possibility arises from research in neuroscience, indicating that immediate and delayed rewards are processed in distinct neural systems. The hippocampus has been shown to be selectively sensitive to delayed feedback, while the ventral striatum has been shown to be selectively sensitive to immediate feedback [27]. Moreover, amnesic patients with damage near the hippocampus are impaired at learning from delayed feedback but not immediate feedback, while patients with striatal dysfunction due to Parkinson’s disease are impaired at learning from immediate feedback but not delayed feedback [28,29]. We also know that animals are impaired at learning when there are longer delays between actions and outcomes [30,31]. Given this evidence that immediate and delayed rewards are processed independently, it seems plausible that these two types of feedback might not be properly integrated into a single, unbiased, total value. While we do not employ brain imaging or patient data in our study, we do explore the implications of two learning systems for behavior.

To study how people integrate dispersed feedback we use a reinforcement learning task in which decision-makers receive feedback about part of the total value immediately after their decision and the second, equally important part, one trial later. Thus, after every decision they need to learn about the option they just chose as well as the option they chose previously. Importantly, the rewards themselves were all delivered at the end of the study session so there was no reason to weight immediate and delayed rewards differently. In some trials, the stimulus with the larger total reward had a smaller immediate reward. Overweighting immediate rewards could lead to errors in these cases. We modeled learning in this task using an extension of a simple RL model [32], embedded in a dynamic choice model [33–36] testing whether our subjects learned from immediate rewards at a higher rate than from delayed rewards. Using eye-tracking, we also tested whether our subjects preferentially gazed at immediate rewards compared to delayed rewards. We correlated the individual learning rate parameters to the proportion of dwell time difference spent on either type of reward to test whether attention could explain any differences in learning rates. We also investigated whether any learning biases were linked to distorted declarative memories for those stimuli. Finally, we also tested whether any learning biases were related to working memory or intertemporal preferences.

To preview our results, we find that people are impaired at learning from delayed feedback, that this immediacy bias is somewhat reflected in aggregate but not individual gaze measures, and that this behavioral bias may be linked to temporal discounting over much longer timescales. Our subjects put roughly twice as much weight on the immediate feedback as the delayed feedback when making their choices. In the aggregate our subjects tended to look more at the immediate feedback than the delayed feedback, though this tendency did not correlate with the behavioral immediacy bias. Interestingly, the behavioral immediacy bias was still present in a passive learning task where subjects observed others making the first 63 decisions. Finally, subjects who showed a greater immediacy bias in the learning task were, in one experiment, more likely to choose a smaller-sooner than larger-later reward in a standard intertemporal choice task with delays on the order of months. In summary, we find evidence that people do not optimally integrate immediate and delayed feedback and that the bias to discount delayed feedback may be linked to a more general tendency to discount the future.

## Results

**Fig 1:**
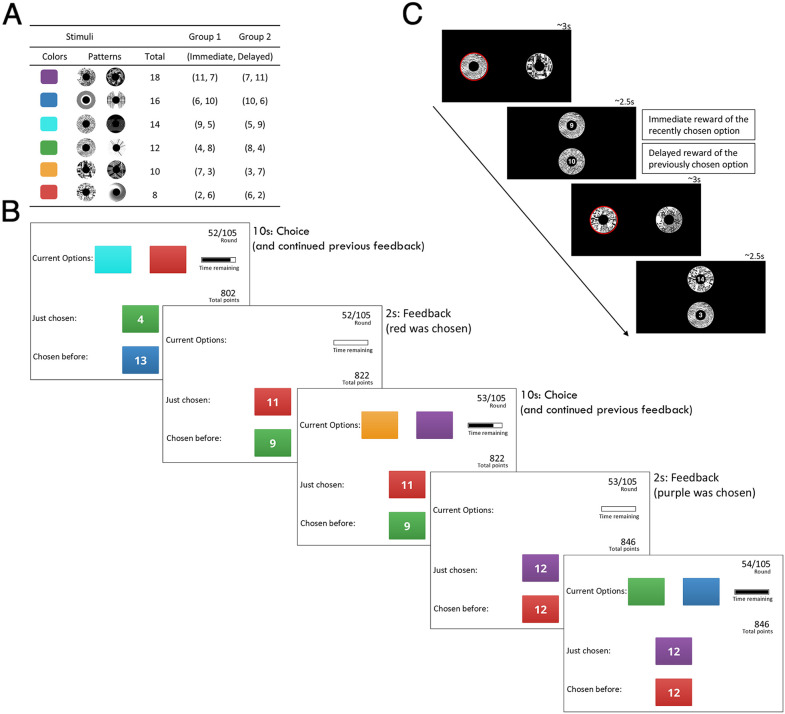
Experimental design. **(A)** For each subject/block there were six unique stimuli, colors in Study 1 or patterns in Study 2. Each stimulus had a total reward value ranging from 8 to 18, in steps of 2. For each total reward value there was an ascending version and a descending version based on whether the immediate feedback was smaller or larger than the delayed feedback, respectively. The two feedback components were set by dividing the total reward in half, adding 2 for the larger component, and subtracting 2 for the smaller. For each total reward value, Group 1 saw the descending or ascending version and Group 2 saw the opposite. A small random number was added to each underlying feedback component to make learning more difficult. Assignment of colors and patterns to total rewards was randomized by subject. **(B)** Study 1 timeline. Each trial, subjects chose between two colored rectangles. Below the current options were the immediate feedback from the previous choice and the delayed feedback from the choice before that. Subjects also saw the trial number, the time within the trial, and the total accumulated points. After each choice, the boxes slid down the screen, feedback was presented, and then new options were shown. **(C)** Study 2 timeline. Each trial, subjects chose between two patterned discs. After each choice, subjects saw the immediate feedback in the center of their chosen disc. Below (or above) that, they saw the delayed feedback in the center of the chosen disc from the previous trial. Study 2 differed from Study 1 in that the feedback did not continue to be shown during the subsequent choice phase; this change was to facilitate eye tracking.

226 subjects learned the values associated with 6 stimuli, represented by colors in Study 1 (Colors Task; N = 102) and patterns in Study 2 (Patterns Task; N = 124; Fig 1). An additional 114 subjects completed Studies 3–4 (Patterns Task), reported in the supplementary material. Each stimulus generated a different number of points. Feedback for each choice was not given at once but split into two components: one shown directly after the choice, and one shown after the following trial’s choice. Thus, each stimulus had an immediate and a delayed feedback component. Both were equally important in determining the subject’s earnings at the end of the study, which were based on the total points earned (Text A in S1 Appendix). Each feedback component had a baseline value (from 2 to 11 points) to which we randomly added up to 4 points in each trial (Fig 1).

To identify whether subjects would overvalue options with higher immediate than delayed feedback, some stimuli had descending feedback (high immediate and low delayed) while others had ascending feedback (low immediate and high delayed). Each study had two groups of subjects and each group had stimuli that alternated between ascending and descending as the stimuli increased in total reward (Fig 1).

There were 105 binary choice trials in each study, separated into blocks of 21 trials with every combination of stimuli. We excluded subjects whose accuracy on trials with both options ascending or descending was below 60 percent. We excluded trials in which the two options were identical as there was no (in)correct answer on such trials. We also excluded the first block of trials (21 trials) since subjects had very little information about the stimuli during that block. Finally, we also excluded trials in which subjects did not choose within the time limit (see Methods).

### Behavior

To preview the behavioral results, we find that subjects displayed a learning bias in favor of immediate feedback. Subjects were more likely to choose a descending option than an ascending option, were more likely to make an error when the ascending option was the correct choice, and put more weight on the immediate feedback than the delayed feedback. Moreover, this bias increased over the course of the experiment.

For the following analyses, we have 87 subjects for Study 1 (43 in Group 1, 44 in Group 2) and 90 subjects for Study 2 (47 in Group 1, 43 in Group 2) after exclusions. We also excluded 1 trial in Study 1 and 61 trials in Study 2 due to subjects missing the time limit.

**Fig 2:**
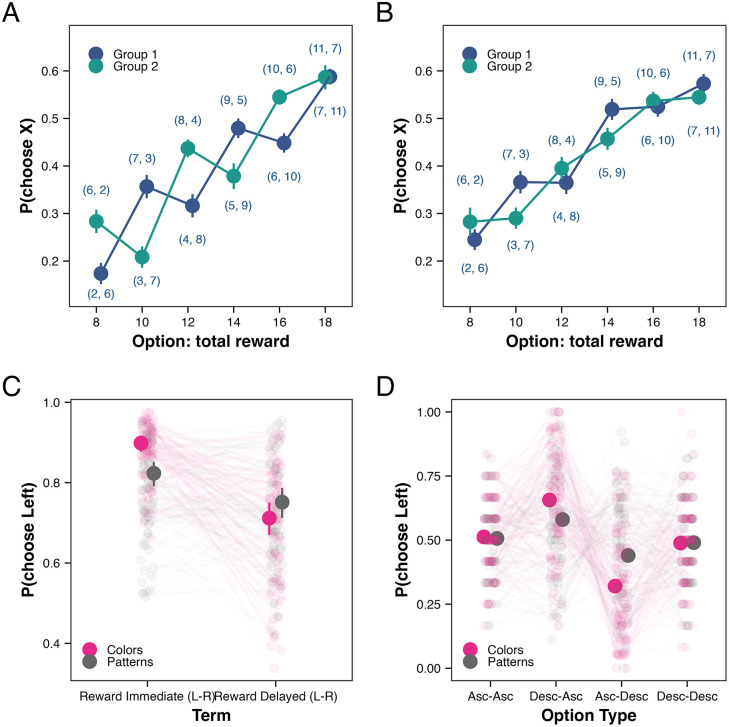
Choice behavior (A,B) Probability of choosing an option as a function of the option’s total reward. **(A)** Study 1 (colors). **(B)** Study 2 (patterns). **(C)** Probability of choosing the left option as a function of the difference in the experienced immediate and delayed feedback, based on a mixed-effects logistic regression. Dots represent subject level effects and bars represent standard errors of the fixed effects. **(D)** Probability of choosing the left option as a function of whether the left and right options, respectively, were descending (Desc) or ascending (Asc). Dots represent subject level averages and bars represent standard errors across subjects.

Looking at subjects’ choices, we found that they put more weight on immediate feedback compared to delayed feedback. For the same total reward, the option with the higher immediate feedback was more likely to be chosen than its counterpart with the lower immediate feedback (Fig 2A,2B). Incongruent trials (worse option descending, better option ascending) had lower accuracy rates than congruent trials (worse option ascending, better option descending) as evidenced by a paired t-test on the average accuracy rates at the subject level (Study 1: congruent *M* = 0*.*88, incongruent *M* = 0*.*53, *t*(86) = -9*.*28*,* 95%*CI* = [-0.42,-0*.*27]*, p <* 10^*−*13^; Study 2: congruent *M* = 0*.*77, incongruent *M* = 0*.*62, *t*(89) = -4*.*34*,* 95%*CI* = [-0.21,-0*.*08]*, p <* 10^*−*4^).

We confirmed these results using regressions of *Choose Left* on differences in the average immediate, delayed, and total rewards between the left and right options, as well as whether the options were ascending or descending. We used mixed-effects regressions with random intercepts and slopes at the subject level. All continuous variables were z-scored. For each trial we calculated the relevant average feedback seen by the subject up to that point in the experiment. Choosing the left option was more likely when it was descending as opposed to ascending (Study 1: *p <* 10^*−*13^; Study 2: *p < .*017; Fig 2D, Table A in S1 Appendix) or when the right option was ascending rather than descending (Study 1: *p <* 10^*−*14^; Study 2: *p <* 10^*−*4^; Fig 2D, Table A in S1 Appendix), controlling for the difference in total reward. Subjects put larger weights on immediate than delayed feedback. When regressing choice on the immediate and delayed feedback differences, the weight on immediate was higher than on delayed by a factor of 2.4 in Study 1 and 1.4 in Study 2. (Study 1: *β*_*Immediate*_ = 2*.*18*,* 95%*CI* = [1*.*97*,*2*.*40]*, β*_*Delayed*_ = 0*.*90 [0*.*71*,*1*.*10]; Study 2: *β*_*Immediate*_ = 1*.*54 [1*.*33*,*1*.*75]*, β*_*Delayed*_ = 1*.*11 [0*.*91*,*1*.*31]; Fig 2C, Table B in S1 Appendix) A Likelihood Ratio Test comparing the immediate and delayed coefficients revealed significant differences (Study 1: *χ*^2^(4*, N* = 87) = 860*.*74*, p <* 10^*−*15^; Study 2: *χ*^2^(4*, N* = 90) = 350*.*1*, p <* 10^*−*15^).

**Fig 3:**
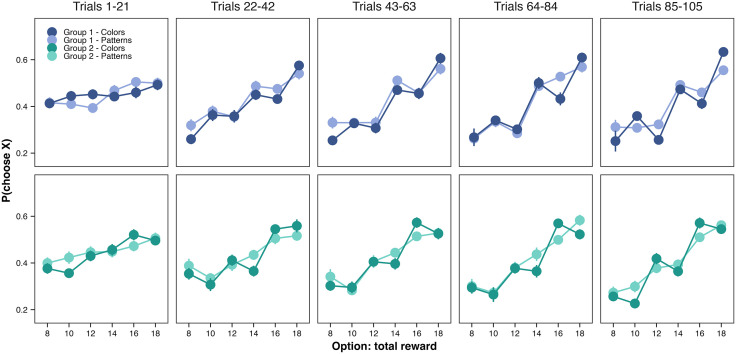
Behavioral bias over time. Probability of choosing a stimulus given it is in the choice set as a function of the total reward of the stimulus. The graphs show both the learning of total values (average slope) and the immediacy bias (zig-zag pattern) across trials. Though visually subtle, statistical analyses reveal that the immediacy bias increases across trials (Table B in S1 Appendix). Dots represent subject level averages and bars represent standard errors across subjects.

The bias to overweight immediate feedback over delayed did not decrease as the experiment progressed; instead, it increased (Fig 3). Building on the previous regression, we included interaction effects between trial number and the immediate/delayed feedback. The interaction of trial number and immediate feedback was positive and significant (Study 1: *β*_*Immediate*:*Trial*_ = 0*.*38*,* 95%*CI* = [0*.*28*,*0*.*49]*, p <* 10^*−*12^; Study 2: *β*_*Immediate*:*Trial*_ = 0*.*34*,* 95%*CI* = [0*.*26*,*0*.*43]*, p <* 10^*−*15^; Table B in S1 Appendix), while the coefficient for the interaction of trial number and delayed feedback was also significantly positive but smaller (Study 1: *β*_*Delayed*:*Trial*_ = 0*.*12*,* 95%*CI* = [0*.*04*,*0*.*20]*, p* = *.*003; Study 2: *β*_*Delayed*:*Trial*_ = 0*.*19*,* 95%*CI* = [0*.*11*,*0*.*26]*, p <* 10^*−*6^; Table B in S1 Appendix). A Likelihood Ratio Test comparing the immediate and delayed interaction coefficients revealed that the increase in the delayed coefficient over time was significantly smaller than the increase in the immediate coefficient over time (Study 1: *χ*^2^(4*, N* = 87) = 860.74*, p <* 10^*−*15^; Study 2: *χ*^2^(4*, N* = 90) = 350.1*, p <* 10^*−*15^). This indicates that the immediacy bias increased over the course of the experiment.

**Fig 4:**
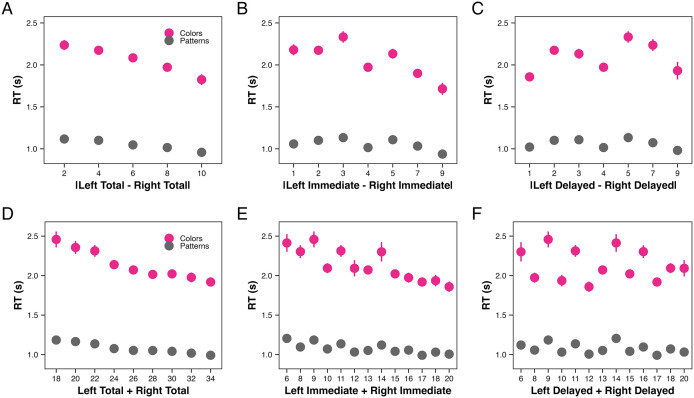
Value difference and overall value effects on response time (RT). (A,B,C) Absolute value difference (|VD|) effects on RT for each study. **(A)** RT decreases with total |VD|. **(B)** RT decreases with immediate |VD|. **(C)** RT does not decrease with delayed |VD|. (D,E,F) Overall value (OV) effects on RT for each study. **(D)** RT decreases with total OV. **(E)** RT decreases with immediate OV. **(F)** RT weakly decreases with delayed OV. Dots and bars represent mean and standard errors across subjects.

The learning bias was also evident in subjects’ response times (RT). We know from past work that people make faster choices when there is a larger absolute value difference (|VD|) between their options or a larger overall (summed) value (OV) of their options (for appealing options) [37–40]. Since subjects’ choices were more influenced by immediate than delayed feedback, we expected to analogously see more influence of immediate than delayed feedback on RT. This was indeed the case. Immediate feedback had a larger effect on RT than delayed for both |VD| and OV. We regressed log(RT) on |VD| and OV, either in total rewards, or separated into immediate and delayed feedback. Larger total |VD| and larger total OV decreased RT (Study 1: *β*_*|VD|*_ = *−*0*.*05 [*−*0*.*07*, −* 0*.*04]*, p <* 10^*−*13^*,β*_*OV*_ = *−*0*.*06 [*−*0*.*08*, −* 0*.*05]*, p <* 10^*−*12^; Study 2: *β*_*|VD|*_ = *−*0*.*04 [*−*0*.*05*, −* 0*.*03]*, p <* 10^*−*9^*, β*_*OV*_ = *−*0*.*05 [*−*0*.*06*, −* 0*.*04]*, p <* 10^*−*10^; Fig 4A,4D & Fig N in S1 Appendix; Table C in S1 Appendix). However, when we separated reward into immediate and delayed components, we found that immediate but not delayed |VD| significantly decreased RT (Study 1: *β*_*VD_Immediate*_ = *−*0*.*04 [*−*0*.*06*, −* 0*.*03]*, p <* 10^*−*9^; *β*_*VD_Delayed*_ = *−*0*.*002 [*−*0*.*01*,*0*.*01]*, p* = *.*786; Study 2: *β*_*VD_Immediate*_ = *−*0*.*02 [*−*0*.*03*, −* 0*.*01]*, p <* 10^*−*3^;*β*_*VD_Delayed*_ = *−*0*.*01 [*−*0*.*02*,*0*.*00]*, p* = *.*056; Fig 4B,4C & Fig N in S1 Appendix, Table D in S1 Appendix). The difference between immediate and delayed effects was significant (Study 1: *χ*^2^(6*, N* = 87) = 114*.*19*, p <* 10^*−*15^; Study 2: *χ*^2^(6*, N* = 90) = 82*.*67*, p <* 10^*−*14^). We also found that immediate OV had a larger effect on RT than delayed OV (Study 1: *β*_*OV Immediate*_ = *−*0*.*07 [*−*0*.*09*, −* 0*.*05]*, p <* 10^*−*12^; *β*_*OV Delayed*_ = *−*0*.*03 [*−*0*.*04*, −* 0*.*01]*, p <* 10^*−*4^; Study 2: *β*_*OV Immediate*_ = *−*0*.*04 [*−*0*.*05*, −* 0*.*03]*, p <* 10^*−*8^; *β*_*OV Delayed*_ = *−*0*.*03 [*−*0*.*04*, −* 0*.*02]*, p <* 10^*−*5^; Difference: Study 1: *χ*^2^(6*, N* = 87) = 95*.*29*, p <* 10^*−*15^; Study 2: *χ*^2^(6*, N* = 90) = 52*.*07*, p <* 10^*−*8^; Fig 4C,4F & Fig N in S1 Appendix, Table D in S1 Appendix).

### Computational model

We sought to capture the relative effect of immediate and delayed rewards using a formal learning model. To do so, we employed an RL model embedded in a drift diffusion model (DDM), but allowed different learning rates for immediate and delayed rewards. We focus on this differential-learning model because our post-task data (described below) indicate an impairment during learning rather than decision-making, but later we also account for possible biases during choice.

We modeled 176 subjects, 87 subjects from Study 1 and 89 subjects from Study 2. One subject was excluded from Study 2’s modeling analyses due to too few valid trials.

We fitted a reinforcement learning drift diffusion model (RLDDM) [33–35]. Given that each stimulus (*s*) was associated with two rewards, one immediate and one delayed (*r*^*I*^*,r*^*D*^), the total value for a stimulus was the sum of the predicted values for the immediate and delayed rewards (*V*). The two values generated two different prediction errors, one for the immediate reward and one for the delayed reward on each trial. The learning rates (*α*^*I*^*,α*^*D*^) controlled how much the values were updated after each reward. In order to determine if learning occurred at different rates for the immediate and delayed rewards, we also fitted a restricted model in which the learning rate was the same for both rewards (*α*^*I*^ = *α*^*D*^ = *α*).

**Fig 5:**
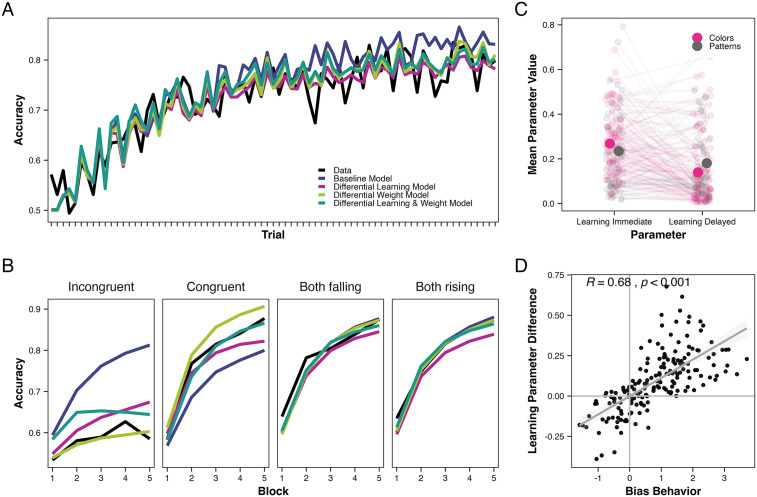
Model parameters and fits. **(A,B)** Average choice accuracy in the data and in the model simulations using the mean posterior values across trials and subjects. **(A)** Experiment level. **(B)** Block level for each type of trial. Incongruent choice sets: worse option descending, better option ascending, Congruent choice sets: worse option ascending, better option descending **(C)** Means and standard errors of the posteriors for the immediate and delayed learning rates. Dots represent subject-level learning rates for each task. **(D)** Correlation between behavioral bias and differential learning rate model bias. Behavioral bias is measured as the difference between the immediate reward coefficient and delayed reward coefficient in the regression of choice on rewards. Learning parameter difference is measured as the difference in the learning rates between the immediate and delayed reward.

Consistent with the model-free analyses, we found higher learning rates for immediate rewards than for delayed rewards. This was true for both studies (Study 1: *M*_*Immediate*_ = 0*.*27*, M*_*Delayed*_ = 0*.*14*, t*(86) = 6*.*97*,* 95%*CI* = [0*.*09*,*0*.*16]*, p <* 10^*−*9^; Study 2: *M*_*Immediate*_ = 0*.*23*, M*_*Delayed*_ = 0*.*18*, t*(88) = 2*.*75*,* 95%*CI* = [0*.*01*,*0*.*09]*, p* = *.*007; Fig 5C). According to subject-level WAIC, the model with differential learning provides a better fit for 48% of our subjects while the model with the same learning rate for both types of rewards fit 52% of our subjects better. Thus, about half of our subjects were better described by two different learning rates.

The differential learning model fits the data well in an absolute sense. It reproduces the learning bias observed in the data; the option with the higher immediate reward was more likely to be chosen than the option with the higher delayed reward, for the same total reward level (Fig K in S1 Appendix). The model also shows increased accuracy across trials (Fig 5A). The model does a good job of predicting accuracy for each type of choice trial (Fig 5B) and the correlation between subject-level predicted accuracy and observed accuracy was high (*r*(174) =*.*67*, p <* 10^*−*15^; Fig L in S1 Appendix). The model predicts behavior more accurately on congruent and incongruent trials compared to when both options were ascending or descending (Fig 5B).

In addition to the differential learning rates, it is also possible that people weight immediate and delayed rewards differently at the time of choice. This could occur either due to unequal attribute weighting [41] or due to discounting delayed feedback, relative to immediate feedback, during learning; these two mechanisms are mathematically equivalent assuming initial values are zero. To account for this possibility, we additionally fit models with different weights on immediate and delayed values, either with or without the different learning rates.

The model with equal learning rates and equal decision weights fit 32% of the participants best. Of the remaining participants, a majority (41%) were best fit by models that included different learning rates (best fits: unequal learning rates (14%), unequal decision weights (28%), or both (27%)). When considering data from all studies (87 for Study 1, 89 for Study 2, 40 for Study 3, 47 for Study 4), again the model with equal learning rates and equal decision weights fit 1/3 (33%) of the participants best. Of the remaining participants, a majority (53%) were best fit by models that included different learning rates (best fits: unequal learning rates (13%), unequal decision weights (31%), or both (22%)) (Table 1).

**Table 1:** Model comparison results using WAIC. Total sample size from Studies 1-4 was 263 subjects. N and P are the number of subjects and proportion of subjects best fitted by each model. Mean (WAIC – WAIC Best) is the mean increase in WAIC for all the models that were not the best model. Sum WAIC is the sum of all WAIC across subjects.

	N	P	Mean (WAIC – WAIC Best)	Sum WAIC
Differential Weight Model	82	0.31	1.24	61100
Differential Learning and Weight Model	59	0.22	1.58	61198
Differential Learning Model	34	0.13	1.88	61306
Baseline Model	88	0.33	4.79	61715

### Eye-tracking

As mentioned in the introduction, attention is one possible explanation for the differential impact of immediate and delayed feedback. To address this, we collected eye-tracking data in Study 2 (and Studies 3–4 in the Text B S1 Appendix) while subjects saw feedback and made their choices. We sought to test whether subjects allocated more gaze to the immediate or delayed feedback, and whether the relative fraction of dwell time on the two feedback components predicted the learning bias. To preview the results, we observed a tendency to dwell longer on the immediate feedback compared to the delayed feedback, but this gaze bias was not correlated with the behavioral bias.

For these analyses, we used 75 subjects, after excluding 15 subjects who did not pass at least three out of four calibration checks throughout the experiment. We did not exclude any trials.

**Fig 6:**
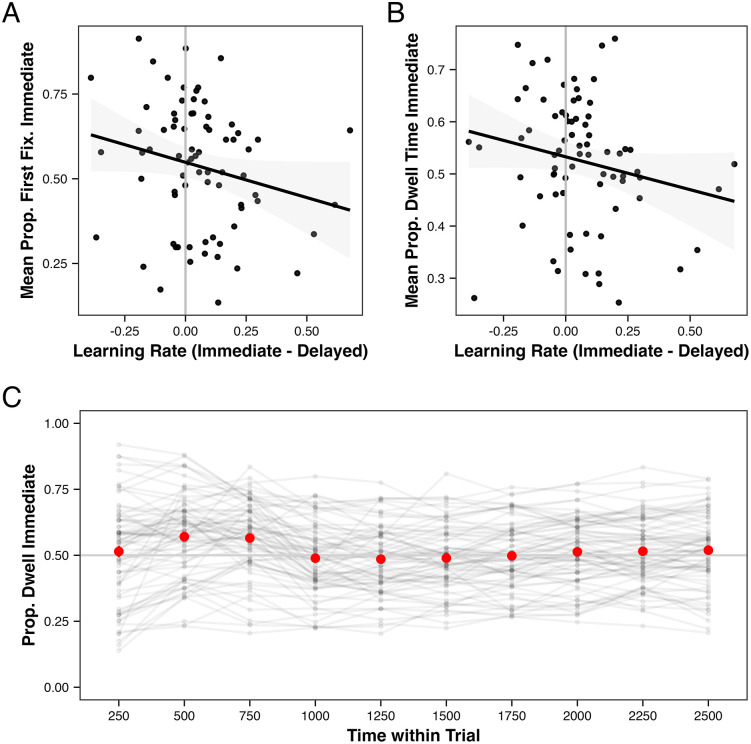
The link between gaze and behavior in Study 2. **(A)** The correlation between the difference in learning rate (immediate vs. delayed) and the proportion of first fixations to the immediate feedback (Spearman’s ρ(73) = *− .*23*, p* = *.*05). **(B)** The correlation between the difference in learning rate (immediate vs. delayed) and the mean dwell proportion to immediate feedback across trials (Spearman’s ρ(73) = *− .*24*, p* = *.*04). **(C)** The mean dwell proportion to immediate feedback within a trial for 250 ms time bins across the trial. Black dots represent each subject within a time bin and red bars are standard errors across subjects. **(A,B)** Dots represent each subject. The gray bands represent 95% CI.

On the whole, subjects tended to look more at the immediate feedback compared to the delayed feedback (Fig 6C). In a mixed-effects regression of relative dwell proportion on immediate vs. delayed, there was a marginal bias in favor of the immediate feedback when controlling for the size and type (ascending vs. descending) of reward (*β* = 0*.*05*,* 95% *CI* = [0*.*00*,*0*.*11]*, p* = *.*070; Bayesian model: posterior mean *β* = 0.05, 95% *CrI* [–0.005, 0.11], Pr(*β* > 0) =.96; Table F in S1 Appendix), and a significant bias when controlling for the predicted values and prediction errors (*β* = 0*.*09*,* 95%*CI* = [0*.*03*,*0*.*16]*, p* = *.*007; Bayesian model: posterior mean *β* = 0.09, 95% *CrI* [0.02, 0.17], Pr(*β* > 0) =.996; Table G in S1 Appendix). Using subject-level regressions, we found that 21 out of 75 subjects had a significant gaze bias towards the immediate feedback and 14 out of 75 had a significant gaze bias towards the delayed feedback.

Subjects were not more likely to fixate first on the immediate compared to the delayed feedback. In a mixed-effects logistic regression of first fixation location (immediate vs. delayed) there was no significant bias towards the immediate feedback, neither when controlling for the size and type of reward (*β* = 0*.*09*,* 95%*CI* = [*−*0*.*12*,*0*.*31]*, p* = *.*379; Bayesian model: posterior mean *β* = 0.10, 95% *CrI* [–0.11, 0.32], Pr(*β* > 0) =.83; Table F in S1 Appendix) nor when controlling for the predicted values and prediction errors (*β* = 0*.*21*,* 95%*CI* = [*−*0*.*07*,*0*.*49]*, p* = *.*138; Bayesian model: posterior mean *β* = 0.21, 95% *CrI* [–0.07, 0.49], Pr(*β* > 0) =.93; Table G in S1 Appendix). There was considerable heterogeneity across subjects in first fixation location. Using subject-level logistic regressions, we found that 12 out of 75 subjects had a significant first-fixation bias towards the immediate feedback and 11 out of 75 subjects had a significant first-fixation bias towards the delayed feedback.

Surprisingly, gaze biases did not positively correlate with choice biases. This was true both when using the behavioral bias calculated from the regressions (Dwell Proportion: Spearman’s ρ (73) = *− .*13*, p* = *.*27; Bayesian analysis *ρ* = -.12, 95% *CrI* [-0.34, 0.10]; First Fixation: Spearman’s ρ (73) = *− .*25*, p* = *.*028; Bayesian analysis *ρ* = -.24, 95% *CrI* [-0.44, -0.02]; Fig J in S1 Appendix) and when using the learning rates from the RL model (Dwell Proportion: Spearman’s ρ(73) = *− .*24*, p* = *.*04; Bayesian analysis *ρ* = -.19, 95% *CrI* [-0.40, 0.03]; First Fixation: Spearman’s ρ(73) = *− .*23*, p* = *.*05; Bayesian analysis *ρ* = -.23, 95% *CrI* [-0.43, -0.01]; Fig 6A,6B). If anything, the correlation was in the opposite direction to what we expected.

However, we did replicate the general finding that options that receive more gaze during the choice phase are more likely to be chosen [16]. A regression of *Choose Left* on the dwell-time difference between left and right options revealed a positive and significant effect controlling for immediate and delayed feedback differences (*β* = 0*.*43*,* 95%*CI* = [0*.*30*,*0*.*56]*, z* = 6*.*36*, p <* 10^*−*9^; Table H in S1 Appendix).

Studies 3–4 (Study 3 online, Study 4 in lab) yielded slightly different findings. In these studies, the correlations between gaze bias to the immediate feedback and choice bias were positive (rather than negative as in Study 2), but were only significant or marginal in Study 3 when looking at the choice bias from the regressions, and marginal in Study 4 when looking at the learning rates. There was also no significant overall dwell bias towards the immediate feedback in either study, though there was a significant first-fixation bias towards the immediate feedback in Study 4 (Figs J, T, U in S1 Appendix). Overall, these additional studies suggest that there may be a link between gaze during the feedback phase and choice bias, but that it is likely a weak relationship.

### Passive learning task

As described earlier, one possible explanation for the observed immediacy bias is agency. People learn better from things that they choose than things they don’t choose, and so it is possible that the immediacy bias is due to an increased sense that immediate vs. delayed feedback is due to participants’ choices.

To investigate this possibility, we conducted an additional experiment with passive learning. N = 57 participants completed this passive learning experiment, using the color paradigm from Study 1. Participants first observed 3 blocks of choices from a Study 1 participant. The feedback and timing of the feedback was the same as that of the matched partner, except that they could not see the foregone choice option on the choice screen. After observing 63 trials, subjects then made 2 blocks (42 trials) of choices on their own.

**Fig 7:**
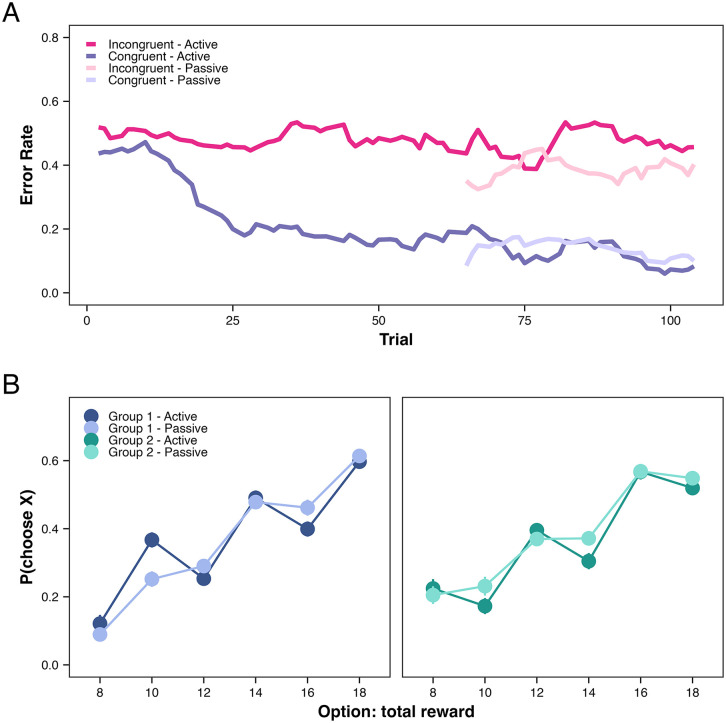
Passive learning experiment (A) Error rates and (B) choice probabilities for the passive learning experiment, compared to Study 1 (active learning matched participants) (A) split into congruent and incongruent trials, and (B) split into Group 1 and Group 2. Darker colors denote the active learning data while lighter colors denote the passive learning data.

Subjects in the passive learning experiment still exhibited a substantial immediacy bias in their choices. When regressing choice on the immediate and delayed feedback differences, the weight on the immediate feedback was substantially higher than the weight on delayed feedback (*β*_*Immediate*_ = 2.62, 95%CI = [2.11, 3.12], *p* < 10^-15^, *β*_*Delayed*_ = 1.48 [1.07, 1.88], *p* < 10^-12^, Fig 7, Table B in S1 Appendix). While the immediacy bias was perhaps smaller than in Study 1 (depending on the analysis, Text A in S1 Appendix), passive learning clearly does not eliminate the effect (Fig 7).

### Declarative memory

At the end of Study 2 (and Study 4), we asked subjects to rank the stimuli in terms of total reward, to indicate whether each stimulus was ascending, descending, or flat, and to estimate the average total reward for each stimulus. The results from these extra measures indicate that the behavioral immediacy bias is at least partly due to inaccurate learning, with memory generally biased in the same direction as subjects’ choices during the main task.

#### Rankings.

Subjects’ rankings were more aligned with the immediate rewards than with the delayed rewards. Using the same ranking responses, we separately computed Kendall tau distance relative to the true immediate-reward ordering and the true delayed-reward ordering. The average ranking error relative to the immediate rewards was 3.23 (Study 4: *SD* = 2.1) and 4.46 (Study 2: *SD* = 2.57), and relative to the delayed rewards was 5.76 (Study 4: *SD* = 2.3) and 5.89 (Study 2: 2.26). These differences are significant (Study 4: *M* = -2.52, *t*(45) = -2.52, *p* < .001; Study 2: *M* = -1.43, *t*(107) = -4.02, *p* < .001). This indicates that subjects encoded the rank order of stimuli primarily through the immediate reward components.

To examine how ranking errors related to choices, we used these same derived measures to separately predict accuracy and bias. A subject who prioritizes immediate rewards should have better alignment of their rankings with the immediate rewards and should behaviorally display more immediacy bias, worse accuracy on incongruent trials, and better accuracy on congruent trials. This is indeed what we observed. Subjects with rankings that were more aligned with the immediate rewards had higher immediacy bias in both studies (linear regression of immediacy bias on ranking error relative to immediate rewards: Study 2: *β* = −0.24, 95%CI = [-0.35,-0.14], *p* < 10^-4^; Study 4: *β* = −0.25, 95%CI = [-0.46,-0.05], *p* = .02; Table J in S1 Appendix). Subjects with rankings that were more aligned with the immediate rewards also had worse choice accuracy on incongruent trials (linear regression of incongruent error rate on ranking error relative to immediate rewards: Study 2: *β* = -0.04, 95%CI = [-0.07,-0.02], *p* < .001; Study 4: *β* = -0.08, 95%CI = [-0.12, -0.04], *p* < .001; Table I in S1 Appendix), and better accuracy on congruent choice sets (linear regression of congruent error rate on ranking error relative to immediate rewards: Study 2: *β* = 0.04, 95%CI = [0.02, 0.06], *p* = .001; Study 4: *β* = 0.05, 95%CI = [0.02, 0.08], *p* < .001; Table I in S1 Appendix).

Alignment with the delayed-reward rankings had somewhat the opposite pattern. Subjects with rankings that were more aligned with the delayed rewards had lower immediacy bias in one study, and higher immediacy bias in the other (linear regression of immediacy bias on ranking error relative to delayed rewards: Study 2: *β* = 0.10, 95%CI = [0.003, 0.201], *p* = .04; Study 4: *β* = -0.25, 95%CI = [−0.43, −-0.07], *p* = .008; Table J in S1 Appendix). Subjects with rankings that were more aligned with the delayed rewards had better choice accuracy on incongruent trials in one study, but not the other (linear regression of incongruent error rate on ranking error relative to delayed rewards: Study 2: *β* = 0.05, 95%CI = [0.02, 0.07], *p* < 10^-4^; Study 4: *β* = 0.02, 95%CI = [-0.02, 0.05], *p* = .33; Table I in S1 Appendix). There was no significant relation between rankings that were more aligned with the delayed rewards and choice accuracy on congruent trials (Table I in S1 Appendix).

In total, these results indicate a relation between subjects’ immediacy bias during the choice task and the alignment of their post-experiment rankings with the immediate rewards.

#### Reward comparisons.

In the reward comparison survey, subjects indicated which feedback component (immediate or delayed) was larger for each stimulus. The prediction for this measure was less obvious. On the one hand, better memory for the rewards should reduce any choice bias. On the other hand, better memory for the distinction between immediate and delayed rewards might indicate more disaggregated processing, which combined with favoring the immediate component, would lead to more immediacy bias. Our findings are more aligned with the latter story, with better memory for this comparison associated with a higher immediacy bias (linear regression of immediacy bias on comparison error: Study 2: *β* = −0.27, 95%CI = [-0.46, -0.09], *p* = .005; Study 4: *β* = −0.26, 95%CI = [-0.55, 0.03], *p* = .008; Table J in S1 Appendix), higher accuracy on congruent choice sets (linear regression of congruent error rate on comparison error: Study 2: *β* = 0.05, 95%CI = [0.01, 0.09], *p* = .03; Study 4: *β* = 0.04, 95%CI = [-0.004, 0.075], *p* = .078; Table I in S1 Appendix), and insignificantly lower accuracy on incongruent choice sets (linear regression of incongruent error rate on comparison error: Study 2: *β* = −0.02, 95%CI = [-0.07, 0.02], *p* = .22; Study 4: *β* = −0.05, 95%CI = [-0.11, 0.04], *p* = .068; Table I in S1 Appendix). Accurate comparative memory may therefore reflect immediate-reward-dominant encoding rather than correcting for it.

#### Point estimates.

In Study 4, subjects separately estimated the average immediate and delayed reward for each stimulus. A subject who prioritizes immediate rewards should have better estimates of the immediate rewards than the delayed rewards. Indeed, subjects made larger absolute errors estimating delayed than immediate rewards (immediate: *M* = 1.98, *SD* = 0.51; delayed: *M* = 2.68, *SD* = 0.73; *t*(45) = −4.97, *p* < 10^-4^), consistent with poorer encoding of delayed reward information. Accuracy in estimating immediate and delayed rewards was associated with higher accuracy on incongruent sets (Immediate: *β* = 0.18, 95%CI = [0.06, 0.31], *p* = .005; Delayed: *β* = 0.12, 95%CI = [0.03, 0.22], *p* = .01; Table I in S1 Appendix), but not significantly associated with immediacy bias (Immediate: *β* = -0.37, 95%CI = [-0.99, 0.26], *p* = .25; Delayed: *β* = 0.04, 95%CI = [-0.45, 0.53], *p* = .86; Table J in S1 Appendix). When we replaced absolute with signed errors, effects were largely non-significant and did not yield consistent or interpretable patterns, indicating that it is the magnitude rather than the direction of memory error that drives these associations. Thus, subjects indeed displayed better memory for immediate than delayed rewards, though memory for the reward components did not correlate with the immediacy bias.

#### Summary.

Across our memory measures, a fairly consistent picture emerges: our subjects displayed better memory for immediate than delayed rewards, and to some extent, this correlated with immediacy bias in the choice task. Also, better memory for the distinction between immediate and delayed rewards correlated with immediacy bias. This pattern suggests that the immediacy bias is not a product of inattention or general memory failure, but reflects asymmetrically better encoding of immediate reward information.

### Working memory

At the end of Study 1, subjects also completed a working-memory task – an n-back task using either n = 2 or n = 3. We regressed the accuracy and d-prime measures for both n-back tasks on the immediacy bias. For the 2-back task, accuracy was marginally negatively related to the immediacy bias based on the regression approach, and significantly so based on the RL model (Regression-based: *β* = *−*2.85 [*−*5.98*,*0*.*28]*, p* = *.*074, RL-based: *β* = *−*0*.*55 [*−*1.02*, −* 0*.*09]*, p* = *.*019; Table K in S1 Appendix). The d-prime measure was also negatively related to the immediacy bias using both measures (Regression-based: *β* = *−*0*.*31 [*−*0*.*60*, −* 0*.*03]*, p* = *.*03, RL-based: *β* = *−*0.05 [*−*0.10*, −* 0*.*01]*, p* = *.*012; Table K in S1 Appendix). However, the association was not present for the 3-back task (Regression-based: *β* = *−*0*.*12 [*−*0*.*54*,*0*.*30]*, p* = *.*556, RL-based: *β* = *−*0*.*02 [*−*0.09*,*0*.*05]*, p* = *.*519; Table K in S1 Appendix).

In Study 4, subjects instead completed a visual working memory task, namely a change localization task. There was no relationship between subjects’ performance on that task and their immediacy bias (Text A in S1 Appendix).

### Discounting preferences

At the end of Study 1 (and Study 4), subjects completed a hypothetical survey to assess their intertemporal preferences. We then regressed the intertemporal indifference point for all three time-scales on the immediacy bias. The only significant association was for the decisions between today and one month. The stronger the behavioral bias, the higher the impatience, significantly for the regression-based analysis (*β* = 2*.*04*,* 95%*CI* = [0*.*15*,*3*.*92]*, p* = *.*034; Table L in S1 Appendix) and marginally for the RL-based analysis (*β* = 11*.*04 [*−*1*.*69*,*23*.*78]*, p* = *.*088; Table L in S1 Appendix). However, this was marginal or not significant for the other time-scales: today and 6 months (Regression-based: *β* = 1*.*86 [*−*0*.*33*,*4*.*05]*, p* = *.*096, RL-based: *β* = 8*.*03 [*−*6*.*79*,*22*.*84]*, p* = *.*284; Table L in S1 Appendix) and 1 month and 6 months (Regression-based: *β* = 1*.*53 [*−*0*.*54*,*3*.*59]*, p* = *.*146, RL-based: *β* = 3*.*63 [*−*10*.*36*,*17*.*632]*, p* = *.*607; Table L in S1 Appendix).

None of these relationships were significant in Study 4 (Table M in S1 Appendix). This casts doubt on the relationship between intertemporal preferences and immediacy bias in our learning task.

## Discussion

In this article we found that people sub-optimally overweight immediate reward feedback relative to delayed reward feedback. In our task, subjects merely had to add together two (small) numbers to determine the total reward from their choice. And yet, subjects put between 1.4 and 2.4 times as much weight on the immediate feedback compared to the delayed feedback, roughly equivalent to counting the immediate feedback twice. This resulted in 15–35% (Study 1: 35%, Study 2: 15%, Study 3: 17%, Study 4: 25%) increases in errors when the option with the larger immediate feedback was the wrong choice. Surprisingly, this immediacy bias only grew stronger as the experiment progressed. At the same time, we also observed an attentional bias towards immediate feedback – subjects spent about 53% of the time looking at the immediate feedback (Studies 2 and 3: 52%, Study 4: 54%). However, this gaze bias generally does not correlate with the choice bias. We also found mixed evidence that this immediacy bias may be linked to impatience and working memory deficits.

Our analyses, which focused on both choice and RT, consistently revealed a stronger influence of immediate feedback on subjects’ choices. This behavioral bias favoring immediate feedback was further evident in RT, with immediate feedback exerting a larger effect on RT via both value difference and overall value. While RT significantly decreased in both the sum of and the difference between the immediate feedback components of the two options, it showed reduced or no such relationship with the delayed feedback, respectively. The relationship between value-difference, overall value, and RT has been well documented [16,17,34,37–40,42–48] and so its absence/reduction for delayed feedback lends further credence to the idea that people prioritize the immediate feedback.

Overall, we find substantial support for an asymmetry in reinforcement learning whereby people learn more slowly about delayed feedback than immediate feedback. We find mixed support for other possible mechanisms – limited attention, agency, and memory accessibility. Our eye-tracking data indicate that limited attention is unlikely to be the whole story. We find only weak evidence that people fixate more on immediate vs. delayed feedback. Our passive-learning data indicate that agency is unlikely to be the whole story. We still find a substantial immediacy bias when participants were yoked through others’ choices, though the bias did decrease somewhat. Finally, our memory tests at the end of the experiments indicate that unequal weighting of immediate vs. delayed feedback is unlikely to be the whole story. Our participants were worse at recalling delayed vs. immediate feedback, and for a majority of them, their behavior was better fit by a model that incorporated different learning rates. Thus, while attention, agency, and memory accessibility might each marginally contribute to an immediacy bias, we conclude that the bulk of the bias is due to asymmetric learning.

We used an RL model embedded in a DDM to better understand and quantify the prioritization of immediate feedback. We incorporated distinct learning rates for immediate and delayed feedback, which provided a better fit for approximately half of the subjects. Immediate learning rates were significantly higher than delayed learning rates at the group level. Although the two-learning-rate model provided generally good fits to the behavior, it isn’t an entirely satisfying explanation for the immediacy bias because while the model can produce growing immediacy bias over short time horizons, it cannot reproduce the continued growth we observed in the data. To confirm this, we generated simulated datasets from the model using fitted subject parameters with low, medium, and high learning rates (all with an immediate:delayed ratio of 2:1), and applied the same mixed-effects logistic regression analysis used on the real data. Across all conditions, the predicted interaction difference (*β*_*Immediate*:*Trial*_ – *β*_*Delayed*:*Trial*_) was centered near zero, while empirical values from Study 1 (0.26) and Study 2 (0.15) fell outside these distributions (all *p <* 0.02). Moreover, the model under-predicts accuracy when both options are ascending or descending (particularly ascending). Future work will need to investigate other mechanisms by which this immediacy bias might occur.

Consistent with a bias in learning, during the feedback phase we observed some evidence for a greater focus on immediate feedback. In Study 2 subjects spent a greater proportion of time dwelling on immediate vs. delayed feedback, while in Study 4 subjects were more likely to look at the immediate feedback first. However, these attentional biases showed inconsistent correlations with the behavioral bias. In Study 2 the correlations were insignificantly negative, while in Studies 3–4 the correlations were positive but only sometimes significant. The eye-tracking results from Studies 2–3 do need to be interpreted with some caution as the data were collected using online webcam-based eye tracking. Collecting eye-tracking data online has many advantages but it does sacrifice some precision relative to in-lab eye-tracking systems [41,49,50]. Thus, we put more weight on our Study 4 results, which revealed a marginally positive correlation between dwell proportion on the immediate feedback and the learning rate advantage for the immediate feedback. Overall, gaze may play a small role in the immediacy bias but it doesn’t seem to fully explain the behavior.

Also consistent with a bias in learning, we found that subjects with a stronger immediacy bias in their choices were also better on the end-of-study memory test where they were asked to report whether each stimulus was ascending, descending, or flat. Moreover, their memory for the rankings or points for the stimuli were, if anything, positively correlated with the immediacy bias. These results indicate that the immediacy bias we observed was not simply due to a lack of attentiveness. Subjects displaying stronger immediacy bias were better at recalling the immediate feedback but not the delayed feedback, suggesting that the immediacy bias may be due to overweighting immediate feedback rather than underweighting delayed feedback.

The act of choosing the alternative that has the higher immediate reward instead of considering how current choices affect future rewards has been termed melioration [51–53]. Melioration at the expense of maximization has been demonstrated in many animal experiments [54,55] as well as with human subjects [56]. Previous research has used the Harvard game to show the tendency of melioration over maximization [57–62]. However, that task puts subjects in a highly complex learning environment in which any deviation from the optimal choice appears impatient. Even Bayesian agents with perfect memory often need thousands of trials to avoid melioration and arrive at the optimal solution. The reason lies in the opacity and complexity of that task. Our task explicitly addresses this criticism by being much simpler and fully transparent and allows us to cleanly attribute the observed bias to overweighting of immediate feedback [63].

Our results are consistent with research in neuroscience that has argued for different learning systems based on immediate and delayed feedback. The dopamine RL response to delayed rewards is similar to rewards that are entirely unpredicted [64,65]. This indicates that the midbrain-striatal system is not entirely able to learn from delayed rewards [66]. Therefore, learning of delayed rewards might be supported by a different system [27]. There is also evidence for distinct involvement of the striatum and hippocampus for different types of feedback. This research argues that learning from immediate rewards is supported by an implicit memory system associated with the ventral striatum (VS), while learning from delayed rewards is supported by an explicit memory system associated with the hippocampus [27,28,66–68]. Both neuroimaging and patient data support the dissociation between these two learning systems – Parkinson’s patients (with VS dysfunction) are impaired at learning from immediate but not delayed feedback, while amnesic patients (with hippocampal dysfunction) are impaired at learning from delayed but not immediate feedback [27–29]*.* There is some debate about whether such evidence conclusively supports multiple systems [69]. It is also unclear whether this multi-system account can explain our results. Unlike these studies, we re-displayed the chosen stimuli when showing both immediate and delayed feedback. So, while the second feedback was delayed relative to the action, it was immediate relative to the stimulus. In any case, future work will need to examine the neural mechanisms underlying the immediacy bias presented here.

Another possibility is that delayed feedback is processed through a Pavlovian rather than instrumental learning system. In the literature, there is a distinction between Pavlovian learning, which depends on stimulus-outcome relations, and instrumental learning, which depends on stimulus-action-outcome relations [70]. In our design, delayed feedback is always the consequence of the participant’s action, making it inappropriate to characterize it as purely Pavlovian. Nevertheless, it is possible that delayed feedback is processed by a Pavlovian learning system that takes over when feedback is temporally distant from the action. Our passive control condition partially addresses this possibility: because participants observed outcomes without acting, any immediacy bias that persists across both active and passive conditions cannot be entirely attributed to differences between instrumental and non-instrumental learning. That said, we acknowledge that fully dissociating the effect of delay from the effect of agency is inherently difficult, since delayed feedback is by definition tied to an action. We did consider additional experiments where feedback is presented for non-chosen stimuli. However, we faced two major challenges. First, presenting feedback for non-chosen stimuli would eliminate subjects’ incentives to make good choices. Solving this problem would lead to major departures from our design. Second, and more importantly, we determined that demonstrating that action-related feedback is overweighted relative to incidental information would not justify concluding that delayed feedback is processed similarly to incidental information. The two biases could be comparable in magnitude, but entirely unrelated. Future work will be needed to resolve this issue, perhaps using neuroimaging to identify whether Pavlovian circuits are recruited during delayed feedback processing.

Our study contributes to the understanding of temporal discounting by providing an alternative explanation for its prevalence and resistance to correction [11]. Unlike traditional intertemporal choice paradigms, which focus on known options and delays, our research highlights the role of learning in shaping impulsive behavior. If reinforcement loses efficacy with delay, immediate consequences would receive undue weight relative to delayed ones. This would lead to a discounting of delayed rewards, not because people do not like to wait, but because the delayed rewards are harder to learn. Thus, the learning bias that our study demonstrates may be a natural explanation for why impatience is so common, while the opposite, future bias, is so rare. Our results offer some evidence in this direction by showing a correlation of behavior in the learning task with typical measures of time discounting. Moreover, if time preferences are influenced by experience rather than inherent traits, discount rates should vary across domains within individuals. Empirical findings support this notion [71].

Our study was designed to rule out temporal discounting effects. First, our delays were on the order of seconds rather than hours or days, as typical in intertemporal choice tasks. Nonetheless, some studies have shown that temporal discounting can occur even with delays on the order of seconds [64,72]. More importantly, reward discounting can not explain the bias because all rewards were delivered at the end of the experiment. One might think that subjects could have willingly foregone monetary reward in order to receive good feedback a few seconds earlier. The high losses observed due to the bias make this implausible. In study 1 for example, subjects on average lost €1.73 in incongruent and €0.64 in congruent trials, meaning that the willingness to pay for good early feedback must have been at least €1.09 to explain the bias in this manner (*t*(86) = -6.75*, p <* 0.001). Finally, the memory tasks indicate that subjects indeed misestimated total rewards as reflected in their choices.

Cognitive load is another potential explanation for learning frictions. Our task required subjects to process feedback from two different choices at the same time, one delayed and one immediate. This could explain why subjects learn incompletely, but it doesn’t explain why they favor the immediate feedback – cognitive load should affect the immediate and delayed feedback equally. Furthermore, once a subject has learned one feedback component (e.g., the immediate one) they should shift their attention and learn the other, leading to a decrease in bias over time. This is the opposite of what we observed. One possibility is that people might be holding in mind the immediate feedback in order to add it to the delayed one. This might explain why the immediate feedback seems to be double counted. Cognitive load has been shown to increase dwell time [73] and this might be one reason why we don’t observe an association between dwell time and the immediacy bias. In any case, future work could study our task in a less constrained setting where immediate and delayed feedback are given in isolation [27].

The prevalence of myopic behavior has significant implications across various domains. In financial decision-making, it can lead to inadequate investments in retirement savings and education, as well as overspending and debt accumulation. Myopic behavior is also associated with health-related choices, where immediate gratification often takes precedence over long-term well-being. Skill development and learning are affected by temporal discounting, as individuals may prioritize immediate enjoyment over the sustained effort required for skill acquisition. This can impact academic and professional performance, personal goals, and overall life satisfaction. This bias could also be relevant in the realm of social media. The focus on immediate rewards, coupled with algorithmic promotion of viral content, can influence users’ sharing and liking behaviors, potentially shaping online discourse and content consumption. Addressing and mitigating the effects of delayed discounting may involve strategies such as enhancing the visibility of future rewards [22] and emphasizing learning through observation.

In summary, we have shown that people show a bias to learn more from immediate feedback than even slightly delayed feedback. This has major implications for how we evaluate people’s choices. As opposed to ascribing impatient behavior to an unwillingness to wait, we argue that impatience may in part be due to delayed rewards receiving too little weight in learning, perhaps due to inattention to delayed feedback. This may explain why impatience and temporal inconsistency are such pervasive problems.

## Materials and methods

### Ethics statement

Study 1 was ruled as exempt by the joint Ethics Committee of the Faculty of Economics and Business Administration of Goethe University Frankfurt (GU) and the Gutenberg School of Management & Economics of the Faculty of Law, Management and Economics of Johannes Gutenberg University Mainz (JGU). Written informed consent was obtained prior to data collection.

Studies 2 & 3 were approved by the Behavioral and Social Sciences Institutional Review Board at the Ohio State University, protocol 2013B0583. Written informed consent was obtained prior to data collection.

Study 4 was ruled as exempt by the UCLA Office of the Human Research Protection Program. Written informed consent was obtained prior to data collection.

### Experimental paradigm

In Study 1, subjects had 10 seconds to make their decisions and lost 5 points if they ran out of time. In Study 2, subjects had 3 seconds and lost 5 points if they ran out of time. In Study 2, the feedback screen was presented for 2 seconds, and a randomly generated time interval between two and six seconds was added after each choice and feedback screen.

Study 1 was programmed using oTree [74]. Study 2 was programmed using jsPsych [75] and utilized webcam-based eye tracking using the Webgazer library [49] integrated in jsPsych [41].

At the end of each study, subjects completed a memory survey. We asked subjects to rank the stimuli in terms of total reward, to indicate whether each stimulus was ascending, descending or flat, and to estimate the average total reward for each stimulus (Text A in S1 Appendix). We only analyzed the memory surveys for Study 2, since the memory survey for Study 1 only asked the questions for a subset of the stimuli presented (Text A in S1 Appendix). For the ranking question we used Kendall tau distance between the true rank and the recalled rank. This measure counts the number of pairwise disagreements between two rankings. For the ascending vs. descending question, we computed the sum of the errors. If the subject answered that the immediate feedback was equal to the delayed feedback, we counted it as half an error. For the total reward question, we calculated the average error.

At the end of Study 1, subjects also completed a temporal discounting task and a working-memory n-back task [76].

In the temporal discounting task, subjects completed a hypothetical survey to assess their intertemporal preferences. In three series of questions, subjects were asked how they would decide between 100 euros today or x in one month; 100 euros today or x in six months; 100 euros in one month or x in six months. By increasing or decreasing x from question to question, we obtained indifference points in the range 100–132 euros for each time horizon.

In the n-back task, participants were presented with a sequence of stimuli, in this case numbers, one at a time. The task required participants to indicate whether the current stimulus matched the one presented n items back in the sequence. One sequence was a 2-back task and consisted of 48 stimuli of which 14 were target stimuli and one sequence was a 3-back task and consisted of 48 stimuli of which 16 were target stimuli.

We assessed performance on the n-back task using accuracy and d prime. Accuracy includes both correctly identified targets and correctly identified non-targets. D prime is a measure of sensitivity or discriminability used in signal detection theory [77] and is calculated as the difference in z-scored hit rates and false alarm rates. These measures were corrected to account for cases in which the hit rate equals 1 or false alarm rate equals 0. In order to correct the measures, we added 0.5 to the total number of hits and false alarms and added 1 to the total number of stimuli [78,79].

The 3-back task was not used for some subjects. In total, 87 subjects completed the 2-back task and 51 subjects completed the 3-back task. 1 subject was excluded from the analyses because their d prime measure was negative, indicating worse-than-chance behavior.

For Study 2 and 3, we excluded some participants based on their eye-tracking data. A short validation phase was presented after trials 21, 42, and 84. After 63 trials participants completed a new calibration and validation. If participants failed all 3 short validations, they were excluded. Each validation check consisted of 3 dots at different positions on the screen that participants had to fixate on. A participant failed the validation if they failed to fixate on any of the 3 dots, within the error margin.

### Modeling

#### Models.

For the differential learning model, the values for the immediate and delayed feedback components were as follows:


$$\begin{array}{c} V_{Ik+\text{1}}(s_{k})=V_{Ik}(s_{k})+\alpha^{I}(r_{Ik}-V_{Ik}(s_{k})) \end{array}$$ (1)

$$\begin{array}{c} V_{Dk+\text{1}}(s_{k})=V_{Dk}(s_{k})+\alpha^{D}(r_{Dk}-V_{Dk}(s_{k})) \end{array}$$ (2)

The total value of a stimulus was the sum of the immediate and delayed values.


$$\begin{array}{c} V_{k}(s_{k})=V_{Ik}(s_{k})+V_{Dk}(s_{k}) \end{array}$$ (3)

Each value was initialized to 0 at *k* = 0.

For the baseline learning model, the learning rates for the immediate and delayed components were the same (*α*^*I*^ = *α*^*D*^ = *α*).

For the differential weight model, the learning rates for the immediate and delayed components were the same but the estimated value of the delayed feedback was weighted less than the estimated value of the immediate feedback. The total value of a stimulus was the sum of the immediate and delayed values:


$$\begin{array}{c} V_{k}(s_{k})=V_{Ik}(s_{k})+\gamma V_{Dk}(s_{k}) \end{array}$$ (4)

where γ is the discount factor on the estimated value of the delayed component.

For the differential learning and weight model, the learning rates and choice weights for the immediate and delayed feedback were allowed to be different. This model was a combination of the differential learning model and the differential weight model.

The decision process itself was described by the DDM [16,80] with one boundary for correct responses and one boundary for incorrect responses. In each trial, the drift rate was defined as the difference between the values from the RL model. When the difference was high, the drift rate was higher, leading to more accurate and faster responses. On each trial, the drift rate was defined as:


$$\begin{array}{c} v\sim d(V_{k,correct}(s_{k})-V_{k,incorrect}(s_{k})) \end{array}$$ (5)

To compare models, we used the Widely Applicable Information Criterion (WAIC) [81]. This criterion is especially useful when the posterior distribution is not Normal. We computed WAIC for each subject and each model.

Model comparison provides only relative performance among models. To check whether the RL model captured the data well, we conducted a posterior predictive check. For each subject, we used the mean of the posterior distribution to generate 100 simulated choices and RTs using their actual sequence of trials. We computed choice accuracy at three levels: (1) at the trial-level when averaging across all subjects (Fig 5A, 5B), (2) at the subject-level when averaging across all trials (Fig L in S1 Appendix), (3) overall level when averaging across both trials and subjects (Fig M in S1 Appendix) and response times across both trials and subjects (Fig N in S1 Appendix) [80]. We excluded trials where the two options were identical.

#### Priors.

We chose weakly informative priors so as to have minimal influence on the posterior [82].


$$\begin{array}{c} \alpha^{I}\sim B(\text{1},\text{1}),\;\text{0}\leq \alpha^{I}\leq \text{1} \end{array}$$ (6)

$$\begin{array}{c} \alpha^{D}\sim B(\text{1},\text{1}),\;\text{0}\leq \alpha^{D}\leq \text{1} \end{array}$$ (7)

$$\begin{array}{c} a\sim N(\text{2},\text{1}),\;a\geq \text{0} \end{array}$$ (8)

$$\begin{array}{c} t\sim U(\text{0},\;minRT) \end{array}$$ (9)

$$\begin{array}{c} d\sim N(\text{0},\;\text{2}),\;d\geq \text{0} \end{array}$$ (10)

$$\begin{array}{c} \gamma \sim B(\text{1},\text{1}),\;\text{0}\leq \gamma \;\leq \;\text{1} \end{array}$$ (11)

#### Fitting procedure.

We used Stan to find the best fitting parameters for each subject separately, using the Monte-Carlo Markov Chain sampling method. For each subject, four chains were run in parallel. Each chain consisted of 4,000 samples out of which 2,000 were warm-up samples. We computed R-hat of all parameters to assess model convergence. This method compares the between- and within-chain estimates for model parameters. The maximum R-hat was 1.01 indicating model convergence [82].

#### Parameter recovery.

To test whether parameters of the models could be recovered well, we simulated one dataset for 176 subjects using the mean posterior of the fitted parameter values and the experimental trials encountered by each subject in the task. We then fit the model on this simulated data. We found large and significant correlations between the true and fitted parameter values and no significant bias in the recovery of the model parameters (Fig L in S1 Appendix).

#### Model recovery.

To test whether the differential learning model and the differential weight model could be distinguished from one another, we examined model recovery. We simulated one dataset for 176 participants using the mean posteriors of the fitted parameter values and the experimental trials encountered by each participant in the task for each model. We then fit each model on these simulated datasets and compared the model fits using WAIC to determine whether the model used to generate the data was also the best-fitting model. To check whether more trials leads to better model recovery, we also simulated datasets for each model using double the number of trials.

The model recovery analyses show that the differential weight model tends to mimic the differential learning model even when the differential learning model generated the data, but not the other way around (Simulated: Differential Learning Model, Fitted: 53% Differential Learning Model, 47% Differential Weight Model; Simulated: Differential Weight Model, Fitted: 72% Weight Model, 28% Differential Learning Model; Fig V in S1 Appendix). This is true even when using double the number of trials in the experiment (Simulated: Differential Learning Model, Fitted: 65% Differential Learning Model, 35% Differential Weight Model; Simulated: Differential Weight Model, Fitted: 71% Weight Model, 29% Differential Learning Model; Fig V in S1 Appendix).

## Supporting information

S1 Appendix Appendix. (PDF)
