# Supplementary material for: Delayed reward information is underweighted in reinforcement learning with dispersed feedback
Source: PLoS Comput Biol. 2026 Jun 29;22(6):e1014459. doi: 10.1371/journal.pcbi.1014459 (PMC13349302; doi:10.1371/journal.pcbi.1014459)
Supplement: S1 Appendix — (PDF) [file pcbi.1014459.s001.pdf]

Supporting Information for

Delayed reward information is underweighted in reinforcement learning with dispersed feedback

Miruna Cotet[1,2+], David Poensgen[3+], Ian Krajbich[4*]

[1] The Ohio State University, Columbus, Ohio, United States of America [2] Complexity Science Hub, Vienna, Austria
[3] Goethe University, Frankfurt, Germany
[4] University of California, Los Angeles, California, United States of America

[+]These authors contributed equally.
[*]Corresponding author: Ian Krajbich

Email: krajbich@ucla.edu

This PDF file includes:

- Text A
- Figures A to X
- Tables A to S
- Text B

Supplementary Text A

Supplementary Methods

Study 1: Colors

Subjects

The experiment was conducted at FLEX (Frankfurt Laboratory for Experimental Economic Research) in February and June 2018. 159 subjects (102 for the active learning condition and 57 for the passive learning condition) were recruited using ORSEE (*1*). All subjects signed a written consent, and the studies received approval from the Joint Ethics Committee for Economics and Business Administration of Goethe University Frankfurt and Gutenberg University Mainz.

Design

Subjects' task in the experiment was to learn the values of 6 abstract stimuli. The stimuli were 6 different colors. These values were initially unknown, and could be learned by sampling. The experiment consisted of 105 trials. In each trial, subjects were presented with two stimuli and could choose one. Each stimulus generated a specific number of points. These points were displayed after each round, allowing subjects to learn throughout the experiment. Payment depended on total points earned, giving subjects an incentive to learn the relative values as quickly and precisely as possible.

Feedback for each choice was not given at once, but split into two components: one shown directly after the choice, one shown with one trial delay. Directly after a choice, the immediate reward was displayed with a clear association with the stimulus chosen. One trial later, the subject would earn the delayed reward for this same choice, again clearly displayed in association with the stimulus (and alongside the immediate feedback of the current trial's choice).

A mixed design was used. Subjects were randomly assigned to one of two experimental conditions. Each of the two groups had to learn a different reward vector associated with the 6 different stimuli. The total values were evenly spaced from 8 to 18. For one group the following levels for the underlying rewards were used (immediate reward, delayed reward): (11, 7), (6, 10), (9, 5), (4, 8), (7, 3), (2, 6) while for the second group the following levels were used: (7, 11), (10, 6), (5, 9), (8, 4), (3, 7), (6, 2). These values were split so that, the immediate reward was 4 points higher or lower than the delayed reward. This arrangement maximized the number of rounds in which subjects had to choose between options close in value, but with opposite temporal profiles.

A small random reward from a uniform distribution consisting of either 1, 2, 3 or 4 points was added to each underlying reward to make learning more difficult. Subjects were informed about this in the instructions. However, the variance was small relative to the value differences between the colors. For options 2 points apart, the chance of the worse option appearing better was only .14 after a single draw for each, and quickly shrank with additional sampling. For stimuli 4 points apart, this probability was .02, and reversal was impossible for larger value differences.

The sequence of 105 decisions was split Into 5 blocks, each with 21 decisions corresponding to all possible two-stimulus pairings. In 6 rounds both options were the same. These choice sets were included for several reasons. Most importantly, they forced subjects to sample each color in regular intervals. This greatly limited the need for deliberate exploration and ensure that subjects would have to sample each color at least once per block. Consequently, even when choices were heavily biased, they would still continuously generate ample evidence to correct this bias.

The trade-off between exploration and exploitation was very limited in this task. This was partly because variance was so small, partly because choice sets rotated in a way which essentially removed any need for deliberate exploration. It ensured that inferior options would soon be sampled anyway: either when up against an even worse option, or itself. The causal structure was explicitly revealed to subjects, and values depended only on the stimuli, so that subjects did not need to infer or memorize states of the world or any rules. Subjects clearly saw which choice had which consequences. Credit attribution was therefore not an issue here.

Block randomization was used to assign subjects to one of the two groups. A pre-fixed sequence of placeholders for stimulus presentation was used which ensured that each two-stimulus combination appeared five times. The pre-fixed sequence was chosen such that if a stimulus was available in round t, it was not available in rounds t + 1 or t + 2. This ensured that subjects had seen as many realizations of the delayed reward as of the immediate reward whenever a stimulus was available. The rule further guaranteed that while the stimulus was available, neither the immediate nor the delayed reward could be on screen. Within each subject, the following aspects of the sequence were randomized: which

stimulus took which place in the sequence, which average reward vector corresponded to which stimulus, left and right positions of the stimuli per round, and which of two sets of stimuli was used.

To address the question of whether passive as opposed to active learning would reduce the bias, an additional condition of the experiment allowed subjects to learn from others' feedback for the first part of the experiment before making their own choices. The passive treatment kept all rules and mechanics of the main experiment with one change: in the first 63 rounds, subjects did not make any decisions, but could learn passively from feedback shown on their screen. The feedback shown was based on the decisions of a matched partner from the main experiment. The feedback and timing of the feedback was the same as that of the matched partner, except that the subject could not see the foregone choice option on the choice screen. This was both to limit similarity to a choice situation, and to not convey information about the beliefs of the subject in the active learning condition to the subject in the passive learning condition. Subjects were not explicitly informed how the data had been generated. Both subjects entered the final 42 rounds with exactly the same information. The sequence of choice sets in these rounds was again kept identical. Instructions were unchanged, with an added section explaining the learning phase.

Procedure

The experiment was programmed in oTree (*78*). An important feature of the experiment was complete transparency of its causal structure. Only the reward vectors were explicitly not disclosed to subjects; they received no information on their range, average or similar. All other mechanics of the experiment were clearly explained in the instructions. Before starting the main task, subjects played an interactive tutorial, which replicated the main task with slight modifications: Detailed on-screen explanations of all rules could be shown and hidden at any time. Instead of 6 colors, 4 shades of gray were used; after a few rounds had been played, their respective values (immediate, delayed) were revealed to make completely transparent how points are generated. The noise terms added to the rewards could be turned on and off at will during the tutorial.

The subjects then completed the main task. Once new options had appeared, subjects had a time limit of 10 seconds per decision, represented by a shrinking bar. If time ran out, a choice was made at random, and a penalty of 5 points deducted. After completing the main task, subjects were asked which of the colors gave the most points, the second most points, and the least points (both components combined). The same color could not be named twice; the questions were not incentivized.

At the end of the task, subjects completed an additional memory questionnaire. The first survey asked: 'Which of these images was associated with the highest (second, third, fourth, fifth highest or lowest) number of points?'. This question was asked only for the best, second best and worst option. The second survey consisted of a Likert scale asking: 'For which image was the first reward larger (smaller or equal) than the second reward'. This question was asked only for the second, third and fourth best options. The third survey asked subjects to estimate the number of points for each image. This question was asked only for the best and second-best options.

Afterwards, the subjects also completed a task to elicit their intertemporal preferences. This was done using a staircase estimator; this procedure is validated against incentivized decisions (*2*). In three series of questions, subjects were asked how they would decide between 100 euros today or x in one month; 100 euros today or x in six months; 100 euros in one month or x in six months. By increasing or decreasing x from question to question, effectively performing bisection search, indifference points in the range 100–132 euros were obtained for each time horizon. By nature of the staircase procedure, more extreme values are censored.

After the experiment, subjects were paid 0.05 euros for each point scored above 1,800 in the task, and nothing if below that threshold. This created steep incentives in the relevant region: Each of the 75 non-degenerate decisions effectively had a stake between 0.10 euros and 0.50 euros. Subjects could score between 1,715 – 2,065 points (ignoring penalties for timeouts), and random behavior was expected to earn 1,890. Subjects were paid an additional 2 euros for completion of the remaining questionnaire. Sessions lasted around 50 minutes. Subjects earned 10.78 euros on average.

Study 2: Patterns Task

Subjects

A total of 374 subjects were recruited from The Ohio State University between February and July 2021. All subjects gave written consent, and the study was approved by the OSU Institutional Review Board (2013B0583). Subjects could

participate in the experiment only if they passed a short initial eye-tracking calibration, a control questionnaire and a second longer eye-tracking calibration. 178 (47%) subjects failed the initial calibration and were paid $2, 49 (13%) subjects failed the control questionnaire and were paid $3 and 23 (6%) subjects failed the second calibration and were paid $4.

A total of 124 subjects completed the experiment. Subjects were paid based on their choices in the task. The minimum payment was $9 for completing the task and the maximum payment was $14. Subjects earned an average of $10. Subjects were paid 4 cents for each point earned above the 1,505 points threshold and could earn a maximum of 350 points. Subjects also lost 5 points if they did not make a decision within 3 seconds.

Our target sample size was 80 subjects who satisfy our exclusion restrictions, 40 for each group. We determined the sample size based on a previous pilot study. Our goal was to obtain at least .80 power to detect a significant Likelihood Ratio Test for whether the immediate reward has a higher coefficient than the delayed reward in a mixed effects logistic regression in which the outcome variable is the choice of the left stimulus and the predictors were the difference between the left and right option in the experienced average immediate reward and delayed reward.

Design

The design was similar to the colors task, with a few modifications that allowed for better eye-tracking measures. First of all, the stimuli were two sets of 6 abstract black and white art images. We choose these images to be similar in salience. The salience was computed using the Graph-based Visual Saliency toolbox in MATLAB (*3*). Secondly, we separated the choice and feedback stages into two separate screens. This was done in order to make it easier to measure subjects' attention to the feedback information. A randomly generated time interval between two and six seconds was added after each choice and feedback screen. Thirdly, subjects had a maximum of 3 seconds to make their choice, instead of 10 seconds. If no choice was made within this time, one stimulus was chosen randomly by the computer. The feedback screen was presented for only 2 seconds. Due to issues with timing on web browsers, the feedback was on average presented for 2.5 seconds. Lastly, the small random reward from a uniform distribution consisting of either 0, 1, 2 or 3 points was added to each underlying reward instead of either 1, 2, 3 or 4 points.

Gaze position was measured using subjects' webcams. We recorded the horizontal (x) and vertical (y) gaze position at each moment in time during the choice and feedback phases of each trial. We defined the Area of Interest (AOI) for the immediate reward as the top half of the screen and the AOI for the delayed reward as the bottom half of the screen.

Procedure

Potential subjects received a link which they could access to complete the experiment. First, subjects completed a consent form. Then they answered a few demographic questions and questions about preferred online payment method. Second, subjects completed a short eye-tracking calibration. Third, they read the instructions and completed a control questionnaire to check their understanding of the instructions. To advance to the second calibration, they had to answer 4 out of 5 questions correctly. After completing the second calibration, they advanced to the experiment.

A short validation phase was given after 21, 42, and 84 trials. After 63 trials subjects completed another calibration and validation. This was done to ensure high eye-tracking data quality. If subjects failed all 3 short validations, they were excluded. Each of the validation checks consisted of showing 3 dots at different positions on the screen that the subjects had to fixate. A subject failed the validation if none of the 3 dots were considered fixated within a certain radius around the dot.

At the end of the choice phase, subjects completed 3 short memory surveys. The first survey asked: 'Which of these images was associated with the highest (second, third, fourth, fifth highest or lowest) number of points?'. The second survey consisted of a Likert scale asking: 'For which image was the first payoff larger (smaller or equal) than the second payoff'. The third survey asked subjects to estimate the number of points for each image.

At the end of the experiment, subjects were shown their final reward and were paid with their preferred online method. Experiments lasted 37 minutes on average.

The analyses were preregistered at OSF (osf.io/mkgqy).

Study 3: Patterns Task Position Feedback Reversed

Subjects

A total of 169 subjects were recruited from Prolific between February and March 2024. All subjects gave their consent, and the studies received approval from the OSU Institutional Review Board (2023E1158). Subjects could participate in the experiment only if they passed a control questionnaire. 112 (66%) subjects failed the control questionnaire. A total of 57 subjects completed the experiment. Subjects were paid based on their choices in the task. The minimum payment was \$9 for completing the task and the maximum payment was \$14. Subjects earned an average of \$10. Subjects were paid 4 cents for each point earned above the 1,505 points threshold and could earn a maximum of 350 points. Subjects also lost 5 points if they did not make a decision within 3 seconds.

Our target sample size was 40 subjects who satisfy our exclusion restrictions. We determined the sample size based on Study's 2 data. Our goal was to obtain at least .80 power to detect a significant Likelihood Ratio Test for whether the immediate reward has a higher coefficient than the delayed reward in a mixed effects logistic regression in which the outcome variable is the choice of the left stimulus and the predictors were the difference between the left and right option in the experienced average immediate and delayed rewards.

Design

The design was the same as the patterns task, except for the position of the feedback on the screen. In Study 1, the immediate reward was always shown on the top half of the screen while the delayed reward was always shown on the bottom half of the screen. Here, we reversed these positions such that the immediate reward was always on the bottom half of the screen while the delayed reward was always on the top half of the screen. This allowed us to test whether the immediacy bias was instead just a spatial bias.

Procedure

The procedure was the same as for Study 1 except that subjects on Prolific did not have to answer any demographic questions or select a preferred payment method. We also did not exclude subjects if they failed the eye-tracking calibration.

The following analyses were preregistered at OSF (osf.io/37qa2).

Study 4: In-lab Eye-tracking Study

Subjects

The experiment was conducted at UCLA (University of California Los Angeles Behavioral Research Lab) in May and November 2024. 57 subjects were recruited for the study. All subjects gave their consent, and the studies received approval from the University of California Los Angeles Review Board. 10 subjects were excluded because their accuracy on the congruent choice sets was below 60%.

Design

The design was the same as the online patterns task (Study 2), except we varied the position of the feedback on the screen between participants. For half of the participant, the immediate reward was always shown on the top half of the screen while the delayed reward was always shown on the bottom half of the screen, while for the other half we reversed these positions such that the immediate reward was always on the bottom half of the screen while the delayed reward was always on the top half of the screen. This allowed us to test whether the immediacy bias was instead just a spatial bias.

Procedure

The procedure was the same as the online patterns task (Study 2) except that subjects did the task in the lab. Subjects also did additional tasks as in Study 1. We replaced the n-back working memory task with a visual memory task, namely the change localization task (*4*). Six colored squares appeared on the screen simultaneously. At the end of the trial, subjects saw the same six squares in their original locations, but one square had changed color. Subjects identified the changed square by pressing the corresponding number on the keyboard. During the test phase, each square was labeled with a number to facilitate response selection.

We also made a change to the declarative memory task. For the point estimation task, subjects had to estimate separately the average points for the immediate and delayed reward.

Subjects were eye-tracked while performing the task. Monocular eye tracking data were collected with a remote EyeLink 1000 Plus system (SR Research Ltd., Mississauga, Ontario, Canada), with a sampling frequency of 500 or 1000 Hz. Before the start of each trial, subjects had to fixate a central fixation cross to ensure that they began each trial fixating on the same location. We recorded the horizontal and vertical gaze position at each moment in time during the trial. The AOIs corresponded to the top and bottom part of the screen for the feedback and the left and right part of the screen for the choice. Similar to Study 2 and 3, subjects had a maximum of 3 seconds to make their choice. If no choice was made within this time, one stimulus was chosen randomly by the computer. The feedback screen was presented for only 2 seconds.

The following analyses were preregistered at OSF (osf.io/tuv48).

Supplementary Results

Colors and Patterns Tasks

Response Times

The learning bias in favor of options with higher immediate rewards was also evident in RT-inferred indifference points. If subjects overweigh immediate rewards, they should be slowest to decide when the descending option is slightly worse than the ascending option. This would correspond to the subjects' average indifference point. To test this hypothesis, we used a mixed-effects quadratic regression with logarithm of RT as the outcome variable. As expected, we found a negative quadratic coefficient for difference in total reward between descending and ascending options (Study 1: $\beta = -0.002, 95\% CI = [-0.003, -0.002], p < 10^{-15}$; Study 2: $\beta = -0.001[-0.002, -0.001], p < 10^{-11}$; Fig E, Table E). Moreover, the estimated peak of this RT curve was at -2.32 for the colors task and -0.81 for the patterns task, indicating that indeed subjects' average indifference point occurred when the descending option was slightly worse than the ascending option.

Passive versus Active Learning

For the passive learning conditions the sample size consisted of 57 subjects. Although there was only marginal evidence that passive learning reduced the immediacy bias when considering experienced average rewards, there was significant evidence for this reduction when considering choices of descending versus ascending options and error rates for congruent and incongruent choice sets.

There was only marginally significant evidence that the bias in the passive learning condition was lower than in the active learning condition when considering the experienced average immediate and delayed rewards. We used a regression of Choose Left on differences in the average immediate and delayed between the left and right options as well as a dummy for the passive learning condition and interactions of this dummy with the average immediate and delayed rewards. The coefficient for the interaction of the experienced average immediate reward and passive learning condition was marginally negative ($\beta_{Immediate:Passive} = -0.49, 95\% CI = [-1.04, 0.07], p = .085$; Table B). The coefficient for the interaction of the experienced average delayed reward and passive learning condition was positive, but not significant ($\beta_{Delayed:Passive} = 0.15, 95\% CI = [-0.31, 0.62], p = .517$; Table B).

We also checked for the immediacy bias by using regression of Choose Left on whether the options were ascending or descending, controlling for total rewards difference between left and right options. As before, we included a dummy for the passive learning condition and interactions of this dummy with whether the options were ascending or descending. There was evidence that the immediacy bias was lower in the passive learning condition when considering choice of descending versus ascending options. Choosing the right option when it was descending was less likely in the passive learning condition compared to the active learning condition as evidenced by a positive interaction coefficient between passive learning condition and whether the right stimulus was descending as opposed to ascending ($\beta = 0.95, 95\% CI = [0.23, 1.66], p = .009$; Table A). The same was not true for when the left option was descending, though the direction of the effect was consistent with a lower bias ($\beta = -0.43, 95\% CI = [-1.12, 0.26], p = .218$; Table A).

Incongruent choice sets had higher error rates in the active learning condition than the passive learning condition when looking at matched pairs of subjects (Active: $M = 0.53, SD = 0.30$; Passive: $M = 0.39, SD = 0.31, t(48) = -2.21, 95\%CI = [-0.28, -0.01], p = .031$). The opposite was true for error rates for congruent choice sets (Active: $M = 0.07, SD = 0.13$; Passive: $M = 0.16, SD = 0.24, t(48) = 2.02, 95\%CI = [0.00, 0.16], p = .049$) indicating that the immediacy bias was lower in the passive learning condition compared with the active learning condition. For these analyses we used only the subjects that passed the exclusion criteria from Study 1. We excluded 15 subjects whose accuracy on trials with both options ascending or descending was below 60 percent.

Reversed Feedback Position

For the following analyses, we combined part of the data from Study 2 with the data from Study 3. We selected the first 40 odd-numbered participants from Study 2 (22 in Group 1, 18 in Group 2) and the first 40 participants from Study 3 (15 in Group 1, 25 in Group 2). We used the same exclusion criteria as before – 71 trials were excluded from Study 3 because subjects were too slow. This plan was pre-registered.

Behavior

For the same total reward level, the option with the higher immediate reward was more likely to be chosen compared to the option with the lower immediate reward (Fig P). We confirmed these results using regressions of Choose Left on differences in the average immediate, delayed, and total rewards between the left and right options, as well as whether the options were ascending or descending. We used mixed-effects regressions with random intercepts and slopes at the subject level. For each trial we calculated the relevant average rewards seen by the subject up to that point in the experiment. We performed these regressions both when pooling the data from the two conditions and when using a dummy variable for the position condition and its interactions with the other variables in the model.

Choosing the left option was more likely when it was descending as opposed to ascending (Studies 2 and 3: $\beta_{LeftFalling} = 0.29, 95\%CI = [0.09, 0.50], p = .005$; Fig P, Table N) or when the right option was ascending rather than descending (Studies 2 and 3: $\beta_{RightFalling} = -0.39, 95\%CI = [-0.60, -0.19], p < 10^{-4}$; Fig P, Table N), controlling for the difference in total rewards. There was no main effect of position of the feedback on the choice of the left option (Studies 2 and 3: $\beta_{ImmediateBottom} = -0.007, 95\%CI = [-0.30, 0.29], p = .961$; Table N). The bias in favor of descending options was even stronger when the immediate reward feedback was at the bottom of the screen compared to when it was at the top of the screen ($\beta_{LeftFalling:ImmediateBottom} = 0.32, 95\%CI = [0.03, 0.61], p = .033$; Fig P, Table N).

Moreover, congruent choice sets had lower error rates than incongruent choice sets (Studies 2 and 3: $\beta_{SetCongruent} = -0.14, 95\%CI = [-0.23, -0.05], p = .003$; Table O). The position of the feedback on the screen did not make a difference on error rates (Studies 2 and 3: $\beta_{ImmediateBottom} = 0.01, 95\%CI = [-0.08, 0.10], p = .748$, $\beta_{SetCongruent:ImmediateBottom} = -0.07, 95\%CI = [-0.19, 0.06], p = .299$; Table O).

Subjects put larger weights on immediate rewards than delayed rewards. When regressing choice on the immediate and delayed reward differences, the weight on the immediate reward was higher than the weight on delayed reward (Studies 2 and 3: $\beta_{Immediate} = 1.48, 95\%CI = [1.26, 1.69], p < 10^{-15}, \beta_{Delayed} = 1.01[0.80, 1.22], p < 10^{-15}$; Fig P, Table P). A Likelihood Ratio Test comparing the immediate and delayed coefficients was significant (Studies 2 and 3: $\chi^2(4, N = 80) = 291.53, p < 10^{-15}$). The position of the feedback did not make a difference (Studies 2 and 3: $\beta_{ImmediateBottom} = 0.08, 95\%CI = [-0.11, 0.28], p = .405, \beta_{Immediate:ImmediateBottom} = 0.004, 95\%CI = [-0.42, 0.43], p = .984, \beta_{Delayed:ImmediateBottom} = -0.04, 95\%CI = [-0.46, 0.37], p = .845$; Fig P, Table P).

To test whether this behavioral bias increased or decreased over the course of the experiments, we added interaction effects with trial number to the previous regression. The interaction of trial number and immediate reward was positive and significant (Studies 2 and 3: $\beta_{Immediate:Trial} = 0.34, 95\%CI = [0.26, 0.43], p < 10^{-14}$; Table P), while the coefficient for the interaction of trial number and experienced delayed reward was also positive, but not significant (Studies 2 and 3: $\beta_{Delayed:Trial} = 0.06, 95\%CI = [-0.01, 0.14], p = .112$; Table P). A Likelihood Ratio Test comparing the immediate and delayed interaction coefficients was significant (Studies 2 and 3: $\chi^2(4, N = 80) = 242.52, p < 10^{-16}$). This indicates that the immediacy bias increases over the course of the experiment. The position of the feedback did not make a difference (Studies 2 and 3: $\beta_{Immediate:Trial:ImmediateBottom} = -0.11, 95\%CI = [-0.28, 0.06], p = .205, \beta_{Delayed:Trial:ImmediateBottom} = -0.03, 95\%CI = [-0.19, 0.12], p = .662$; Table P).

Response Times

Larger total |VD| and larger total OV decreased RT (Studies 2 and 3: $\beta_{|VD|} = -0.03[-0.04, -0.02], p < 10^{-5}, \beta_{OV} = -0.04[-0.06, -0.03], p < 10^{-7}$; Fig R, Table Q). Position of the feedback did not make a difference ($\beta_{|VD|:ImmediateBottom} = 0.004[-0.02, 0.03], p = .723, \beta OV : ImmediateBottom = 0.0005[-0.03, 0.03], p = .970$; Fig R, Table Q). When we separated value into immediate and delayed rewards, we also found that both immediate and delayed |VD| decreased RT (Studies 2 and 3: $\beta_{VDImmediate} = -0.03[-0.04, -0.02], p < 10^{-7}; \beta_{VDDelayed} = -0.02[-0.03, -0.01], p = .001$; Fig R, Table R). The difference between immediate and delayed was significant (Studies 2 and 3: $\chi^2(1, N = 80) = 3.851, p = .05$). Position of the feedback did not make a difference ($\beta_{VDImmediate:ImmediateBottom} = -0.002[-0.02, 0.02], p = .838; \beta_{VDDelayed:ImmediateBottom} = -0.001[-0.02, 0.02], p = .918$; Fig R, Table R). We did not find that immediate OV had a larger effect on RT than delayed OV (Studies 2 and 3: $\beta_{OVImmediate} = -0.03[-0.04, -0.02], p < 10^{-10}; \beta_{OVDelayed} = -0.03[-0.04, -0.02], p < 10^{-8}; \chi^2(1, N = 80) = 0.4542, p = .50$; Fig R, Table R). Position of the feedback did not make a difference ($\beta_{OVImmediate:ImmediateBottom} = -0.01[-0.03, 0.01], p = .315; \beta_{OVDelayed:ImmediateBottom} = -0.008[-0.01, 0.03], p = .423$; Fig R, Table R).

The learning bias in favor of options with higher immediate rewards was also evident in subjects' RT-inferred indifference points. We found a negative quadratic coefficient for difference in total reward between descending and ascending options (Studies 2 and 3: $\beta_{Total} = -0.001, 95\%CI = [-0.001, -0.001], p < 10^{-4}$; Fig E, Table S). Moreover, the estimated peak of this RT curve was at -1.26 indicating that the subject's indifference point occurred when the descending option was slightly worse than the ascending option, as expected. The position of the feedback did not make a difference (Studies 2 and 3: $\beta_{Total:ImmediateBottom} = -0.001, 95\%CI = [-0.008, 0.006], p = .707, \beta_{TotalSq:ImmediateBottom} = -0.00006, 95\%CI = [-0.001, 0.001], p = .902$; Table S).

Model

For the differential learning model, the learning rate for the immediate reward was higher than for the delayed reward for reversed feedback position condition (Study 3: $M_{Immediate} = 0.28, M_{Delayed} = 0.20, t(39) = 2.96, 95\%CI = [0.030.15], p = .005$; Fig P). According to subject level WAIC, the model with differential learning provides a better fit for 57% of our subjects while the model with the same learning rate for both types of rewards fit 42% of our subjects better.

For the weight model, we found lower weight on the delayed rewards compared with immediate rewards (Study 3: $M_{Delayed} = 0.52, t(39) = -14.23, 95\%CI = [0.45, 0.59], p < 10^{-15}$).

Using all models (Baseline Model, Differential Learning Model, Differential Weight Model and Differential Learning and Weight Model), the model with same learning rates and equal decision weights provides a better fit for 35% subjects, the model with differential learning rate and equal weights provides a better fit for 15% subjects, the model with equal learning rates and different weights provides a better fit for 27% of subjects, while the model with both different learning rates and different weights provides a better fit for 23% subjects.

Gaze Bias

For these analyses, we used 18 subjects from Study 3, after excluding 22 subjects who did not pass the initial calibration or did not pass at least three out of four validation checks throughout the experiment. We did not exclude any trials. On the whole, subjects did not have a tendency to look more at the immediate reward compared to the delayed reward (Fig T). In a mixed-effects regression of relative dwell proportion on immediate vs. delayed, the effect (i.e., intercept) was not significant when controlling for the size and type (ascending vs. descending) of reward ($\beta = -0.02, 95\%CI = [-0.13, 0.09], p = .733$; Table F), and also not significant when controlling for the predicted values and prediction errors ($\beta = -0.03, 95\%CI = [-0.17, 0.11], p = .664$; Table G).

Subjects were not more likely to fixate first to the immediate reward compared to the delayed reward. In a mixed-effects logistic regression of first fixation location (immediate vs. delayed) the effect (i.e., intercept) was not significant when controlling for the size and type of reward ($\beta = 0.31, 95\%CI = [-0.13, 0.75], p = .171$; Table F) nor when controlling for the predicted values and prediction errors ($\beta = 0.02, 95\%CI = [-0.59, 0.63], p = .958$; Table G).

The learning bias was significantly correlated with the first fixation bias but not with the dwell proportion bias. However, this was true only when using the behavioral bias calculated from the regressions (Dwell Proportion: $\rho(16) = .24, p = .33$; First Fixation: $\rho(16) = .49, p = .04$) but not when calculated from the RL model (Dwell

Proportion: $\rho(16) = .11, p = .65$; First Fixation: $\rho(16) = .33, p = .19$; Fig T). However, in this study the correlations were in the expected direction.

In-lab Eye-tracking Study

We used the same exclusion criteria as before, results in 99 trials being excluded from Study 4 because subjects were too slow. This plan was pre-registered.

Behavior

For the same total reward level, the option with the higher immediate reward was more likely to be chosen compared to the option with the lower immediate reward (Fig Q). We confirmed these results using regressions of Choose Left on differences in the average immediate, delayed, and total rewards between the left and right options, as well as whether the options were ascending or descending. We used mixed-effects regressions with random intercepts and slopes at the subject level. For each trial we calculated the relevant average rewards seen by the subject up to that point in the experiment. We performed these regressions both when pooling the data from the two conditions and when using a dummy variable for the position condition and its interactions with the other variables in the model.

Choosing the left option was more likely when it was descending as opposed to ascending ($\beta_{LeftFalling} = 0.37, 95\%CI = [-0.07, 0.81], p = .096$; Fig Q, Table N) or when the right option was ascending rather than descending ($\beta_{RightFalling} = -0.57, 95\%CI = [-1.03, -0.11], p = .016$; Fig Q, Table N), controlling for the difference in total rewards. However, when including the difference in total experienced rewards, the effects reversed ($\beta_{LeftFalling} = -1.80, 95\%CI = [-2.48, -1.13], p < 10^{-6}$, $\beta_{RightFalling} = 1.74, 95\%CI = [1.07, 2.42], p < 10^{-6}$, $\beta_{ImmediateBottom} = -0.21, 95\%CI = [-0.68, 0.27], p = .393$ $\beta_{LeftFalling:ImmediateBottom} = 0.57, 95\%CI = [-0.42, 1.55], p = .258$, $\beta_{RightFalling:ImmediateBottom} = -0.52, 95\%CI = [-1.50, 0.46], p = .299$; Table N). While this goes against the results of Studies 1-3, we also test for the bias in a different way below, using the points of the immediate and delayed rewards instead of whether an option was rising or falling, and find strong evidence for the immediacy bias. Position of the feedback did not have a significant effect.

Congruent choice sets had lower error rates than incongruent choice sets which is consistent with results from Studies 1-3 and indicate that a behavioral bias in favor of the immediate reward is present ($\beta_{SetCongruent} = -0.19, 95\%CI = [-0.31, -0.07], p = .002$; Table O). The position of the feedback on the screen did not make a difference on error rates ($\beta_{ImmediateBottom} = 0.10, 95\%CI = [-0.02, 0.22], p = .110$, $\beta_{SetCongruent:ImmediateBottom} = -0.13, 95\%CI = [-0.30, 0.04], p = .144$; Table O).

Subjects put larger weights on immediate rewards than delayed rewards. When regressing choice on the immediate and delayed reward differences, the weight on the immediate reward was higher than the weight on delayed reward ($\beta_{Immediate} = 1.49, 95\%CI = [1.20, 1.78], p < 10^{-15}, \beta_{Delayed} = -0.35[-0.55, -0.16], p < 10^{-3}$; Fig Q, Table P). A Likelihood Ratio Test comparing the immediate and delayed coefficients was significant ($\chi^2(4, N = 47) = 578.17, p < 10^{-15}$). The position of the feedback did not make a difference ($\beta_{ImmediateBottom} = -0.22, 95\%CI = [-0.55, 0.10], p = .183, \beta_{Immediate:ImmediateBottom} = 0.15, 95\%CI = [-0.42, 0.72], p = .615, \beta_{Delayed:ImmediateBottom} = -0.11, 95\%CI = [-0.50, 0.28], p = .580$; Fig Q, Table P).

To test whether this behavioral bias increased or decreased over the course of the experiments, we added interaction effects with trial number to the previous regression. The interaction of trial number and immediate reward was positive and significant ($\beta_{Immediate:Trial} = 0.29, 95\%CI = [0.18, 0.41], p < 10^{-6}$; Table P), while the coefficient for the interaction of trial number and experienced delayed reward was negative and significant ($\beta_{Delayed:Trial} = -0.25, 95\%CI = [-0.35, -0.14], p < 10^{-5}$; Table P). A Likelihood Ratio Test comparing the immediate and delayed interaction coefficients was significant ($\chi^2(4, N = 47) = 2922.2, p < 10^{-15}$). This indicates that the immediacy bias increases over the course of the experiment. The position of the feedback did not make a difference ($\beta_{Immediate:Trial:ImmediateBottom} = -0.002, 95\%CI = [-0.23, 0.23], p = .983, \beta_{Delayed:Trial:ImmediateBottom} = -0.04, 95\%CI = [-0.25, 0.17], p = .704$; Table P).

Response Times

Larger total |VD| and larger total OV decreased RT, but larger total |VD| was only marginally significant ($\beta_{|VD|} = -0.02[-0.040, 0.001], p = .067, \beta_{OV} = -0.03[-0.04, -0.01], p = .005$; Fig S, Table Q). Position of the immediate feedback at the bottom of the screen did not make a difference for |VD|, but it

decreased RT for OV consistent with a stronger effect ($\beta_{|VD|:ImmediateBottom} = -0.02[-0.06, 0.02], p = .305, \beta_{OV:ImmediateBottom} = -0.04[-0.074, -0.003], p = .033$; Fig S, Table Q). When we separated value into immediate and delayed rewards, we found that only immediate |VD| decreased RT, while delayed |VD| increases RT suggesting a strong bias towards considering the immediate rewards more than the delayed rewards ($\beta_{|VD|Immediate} = -0.04[-0.05, -0.02], p < 10^{-8}; \beta_{|VD|Delayed} = 0.01[0.002, 0.025], p = .022$; Fig S, Table R). The difference between immediate and delayed was significant ($\chi^2(1, N = 47) = 34.502, p < 10^{-8}$). Position of the immediate feedback at the bottom of the screen decreased RT further, but only for the immediate |VD|, while it did not make a difference for the delayed |VD| ($\beta_{|VD|Immediate:ImmediateBottom} = -0.05[-0.07, -0.03], p < 10^{-4}; \beta_{|VD|Delayed:ImmediateBottom} = -0.01[-0.01, 0.04], p = .244$; Fig S, Table R). We also found that only immediate OV decreased RT, while delayed OV increased RT suggesting a strong bias towards considering the immediate rewards more than the delayed rewards. The difference between immediate and delayed was significant ($\beta_{OVImmediate} = -0.03[-0.04, -0.02], p < 10^{-7}; \beta_{OVDelayed} = 0.02[0.01, 0.03], p = .002; \chi^2(1, N = 47) = 32.693, p < 10^{-7}$; Fig S, Table R). Position of the immediate feedback at the bottom of the screen decreased RT further for higher immediate OV and increased RT for higher delayed OV ($\beta_{OVImmediate:ImmediateBottom} = -0.04[-0.07, -0.02], p < 10^{-3}; \beta_{OVDelayed:ImmediateBottom} = 0.03[0.002, 0.048], p = .034$; Fig S, Table R).

The learning bias in favor of options with higher immediate rewards was also evident in subjects' RT-inferred indifference points. We found a negative quadratic coefficient for difference in total reward between descending and ascending options ($\beta_{Total} = -0.007, 95\%CI = [-0.013, -0.002], p = .012$; Fig E, Table S). Moreover, the estimated peak of this RT curve was at -1.26 indicating that the subject's indifference point occurred when the descending option was slightly worse than the ascending option, as expected. The position of the immediate feedback at the bottom of the screen had an effect only on the linear coefficient shifting the indifference point to -5.25 increasing the observed bias ($\beta_{Total:ImmediateBottom} = -0.01, 95\%CI = [-0.025, -0.004], p = .007, \beta_{TotalSq:ImmediateBottom} = -0.0001, 95\%CI = [-0.001, 0.001], p = .771$; Table S).

Model

For the differential learning model, the learning rate for the immediate reward was higher than for the delayed reward ($M_{Immediate} = 0.24, M_{Delayed} = 0.14, t(46) = 5.26, 95\%CI = [0.07, 0.15], p < 10^{-5}$; Fig Q). According to subject level WAIC, the model with differential learning provides a better fit for 60% of our subjects while the model with the same learning rate for both types of rewards fit 40% of our subjects better. When the immediate feedback was at the top (bottom), 36% (45%) of subjects were better fit by the model with differential learning compared to 64% (55%) by the model with the same learning rate. Thus, according to the model comparison, the learning bias in favor of the immediate reward was stronger when the immediate reward feedback was at the bottom of the screen.

For the weight model, we found lower weight on the delayed rewards compared with immediate rewards (Study 4: $M_{Delayed} = 0.42, t(46) = -29.92, 95\%CI = [0.38, 0.46], p < 10^{-15}$).

Using all models (Baseline Model, Differential Learning Model, Differential Weight Model and Differential Learning and Weight Model), the model with same learning rates and equal decision weights provides a better fit for 38% subjects, the model with differential learning rate and equal weights provides a better fit for 8% subjects, the model with equal learning rates and different weights provides a better fit for 47% of subjects, while the model with both different learning rates and different weights provides a better fit for 6% subjects.

Gaze Bias

On the whole, subjects did not have a tendency to look more at the immediate reward compared to the delayed reward. In a mixed-effects regression of relative dwell proportion on immediate vs. delayed, the effect (i.e., intercept) was not significant when controlling for the size and type (ascending vs. descending) of reward ($\beta = 0.002, 95\%CI = [-0.05, 0.06], p = .929$; Table F), and also not significant when controlling for the predicted values and prediction errors ($\beta = 0.03, 95\%CI = [-0.04, 0.10], p = .444$; Table G). The position of the immediate feedback at the bottom of the screen did not have a significant effect ($\beta_{ImmediateBottom} = -0.05, 95\%CI = [-0.15, 0.06], p = .381$; Table F).

Unlike in the online eye-tracking experiment, subjects were more likely to fixate first to the immediate reward compared to the delayed reward. In a mixed-effects logistic regression of first fixation location (immediate vs. delayed) the effect (i.e., intercept) was significant when controlling for the size and type of reward ($\beta = 0.98, 95\%CI = [0.52, 1.45], p < 10^{-4}$; Table F) and when controlling for the predicted values and prediction

errors ($\beta = 0.67, 95\%CI = [0.14, 1.21], p = .014$; Table G). However, position of the immediate feedback at the bottom of the screen made a significant difference, with subject being more likely to fixate first on the delayed reward ($\beta = 1.85, 95\%CI = [1.35, 2.35], p < 10^{-12}$; $\beta_{ImmediateBottom} = -1.91, 95\%CI = [-2.61, -1.22], p < 10^{-7}$; Table G).

The learning bias was significantly correlated with the first fixation bias but not with the dwell proportion bias. However, this was not true when using the behavioral bias calculated from the regressions (Dwell Proportion: $\rho(45) = -.13, p = .4$; First Fixation: $\rho(45) = .01, p = .92$) but only when using the learning bias calculated from the RL model (Dwell Proportion: $\rho(45) = .29, p = .05$; First Fixation: $\rho(45) = .23, p = .12$; Fig U).

Working Memory

At the end of the study, subjects also completed a visual working memory task, namely a change localization task (*4*). Six colored squares appeared on the screen simultaneously. At the end of the trial, subjects saw the same six squares in their original locations, but one square had changed color. Subjects identified the changed square by pressing the corresponding number on the keyboard. During the test phase, each square was labeled with a number to facilitate response selection.

We regressed behavioral and learning biases on the accuracy for the working memory task. Accuracy was not significantly related to the behavioral or learning bias (Regression-based: $\beta = 0.65[-1.93, 3.22], p = .615$, RL-based: $\beta = 0.32[-0.04, 0.68], p = .081$; Table K).

Discounting Preferences

We then regressed the intertemporal indifference point for all 3 time scales on the behavioral and learning-rate biases. There was no significant association between impatience and the behavioral bias (Regression-based: $\beta_{Today-1Month} = -2[-5.25, 1.24], p = .220$, RL-based: $\beta_{Today-1Month} = -10.34[-33.10, 12.42], p = .365$, Regression-based: $\beta_{Today-6Months} = -1.94[-5.55, 1.66], p = .283$, RL-based: $\beta_{Today-6Months} = -11.13[-36.31, 14.05], p = .378$, Regression-based: $\beta_{1Month-6Months} = -1.48[-4.99, 2.04], p = .402$, RL-based: $\beta_{1Month-6Months} = -19.45[-43.40, 4.50], p = .109$; Table M). Unlike in Study 1, the higher the impatience, the lower the immediacy bias, but no effects were significant.

Additional References

1. Greiner, B. Subject pool recruitment procedures: organizing experiments with ORSEE. *J Econ Sci Assoc* **1**, 114–125 (2015).
2. Falk, A., Becker, A., Dohmen, T., Huffman, D. & Sunde, U. The Preference Survey Module: A Validated Instrument for Measuring Risk, Time, and Social Preferences. *IZA Discussion Paper* **9674** 1–66 (2016).
3. Harel, J., Koch, C. & Perona, P. Graph-Based Visual Saliency. in *Advances in Neural Information Processing Systems 19* (eds. Schölkopf, B., Platt, J. & Hofmann, T.) 545–552 (The MIT Press, 2007).
4. Zhao, C., Vogel, E. & Awh, E. Change localization: A highly reliable and sensitive measure of capacity in visual working memory. *Atten Percept Psychophys* **85**, 1681–1694 (2023).

Supplementary Figures

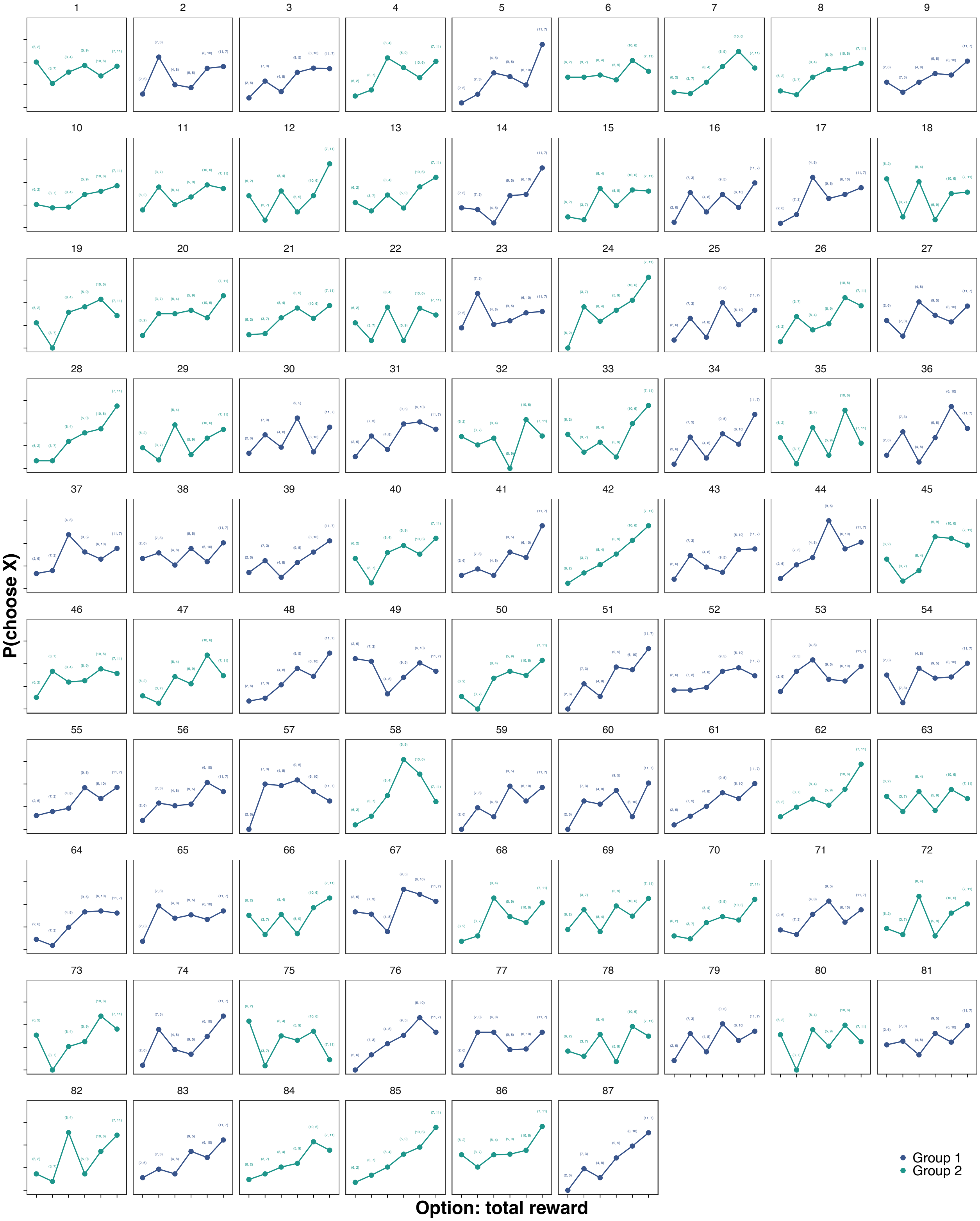

Figure A. Behavioral bias at the subject level for Study 1 Colors. Probability of choosing an option as a function of the option's total reward for Study 1 Colors.

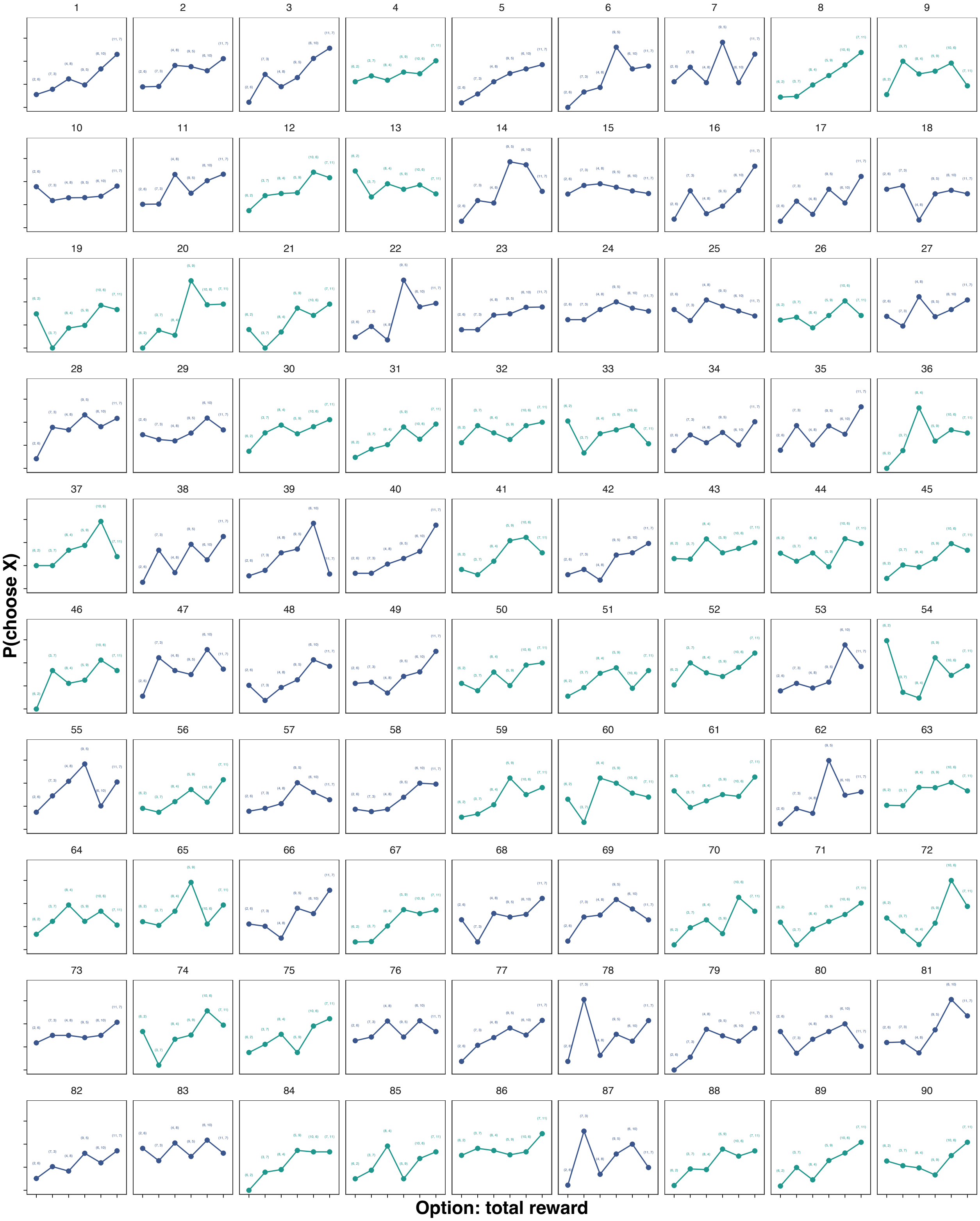

Figure B. Behavioral bias at the subject level for Study 2 Patterns. Probability of choosing an option as a function of the option's total reward for Study 2 Patterns.

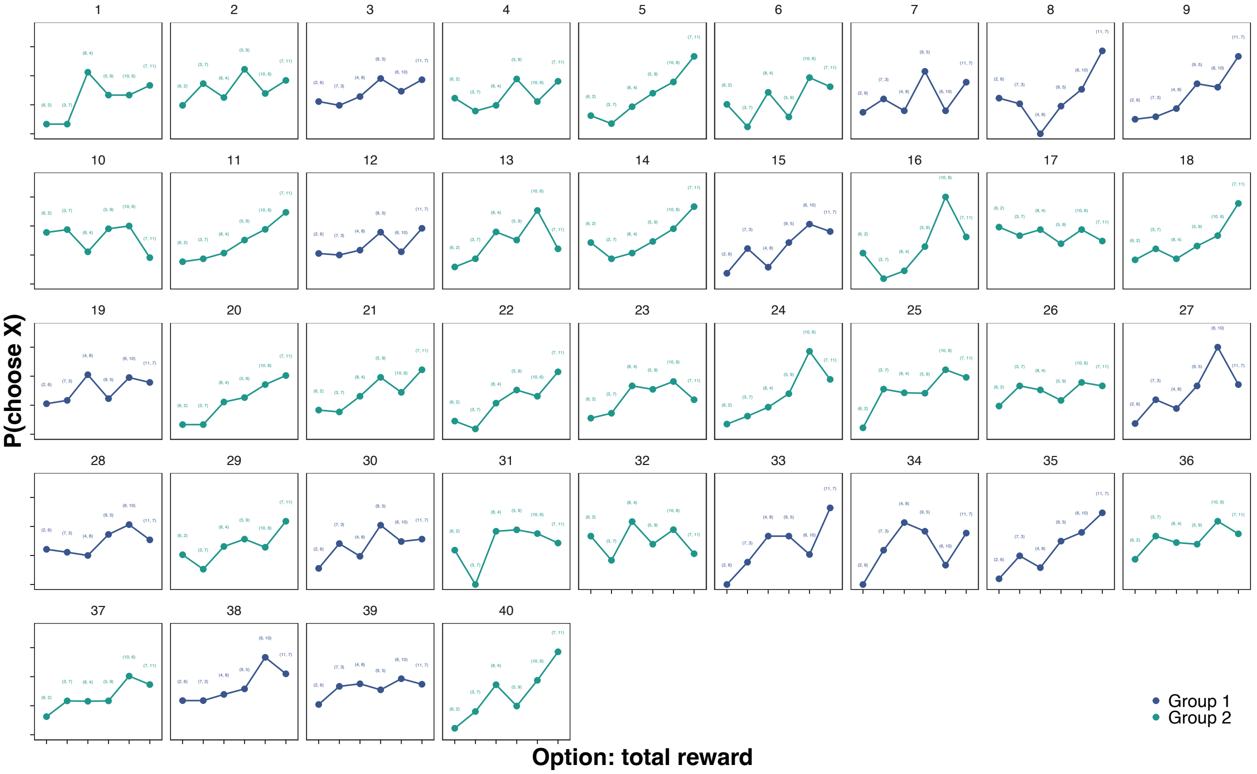

Figure C. Behavioral bias at the subject level for Study 3 Patterns Reversed Feedback Position. Probability of choosing an option as a function of the option's total reward for Study 3 Patterns Reversed Feedback Position.

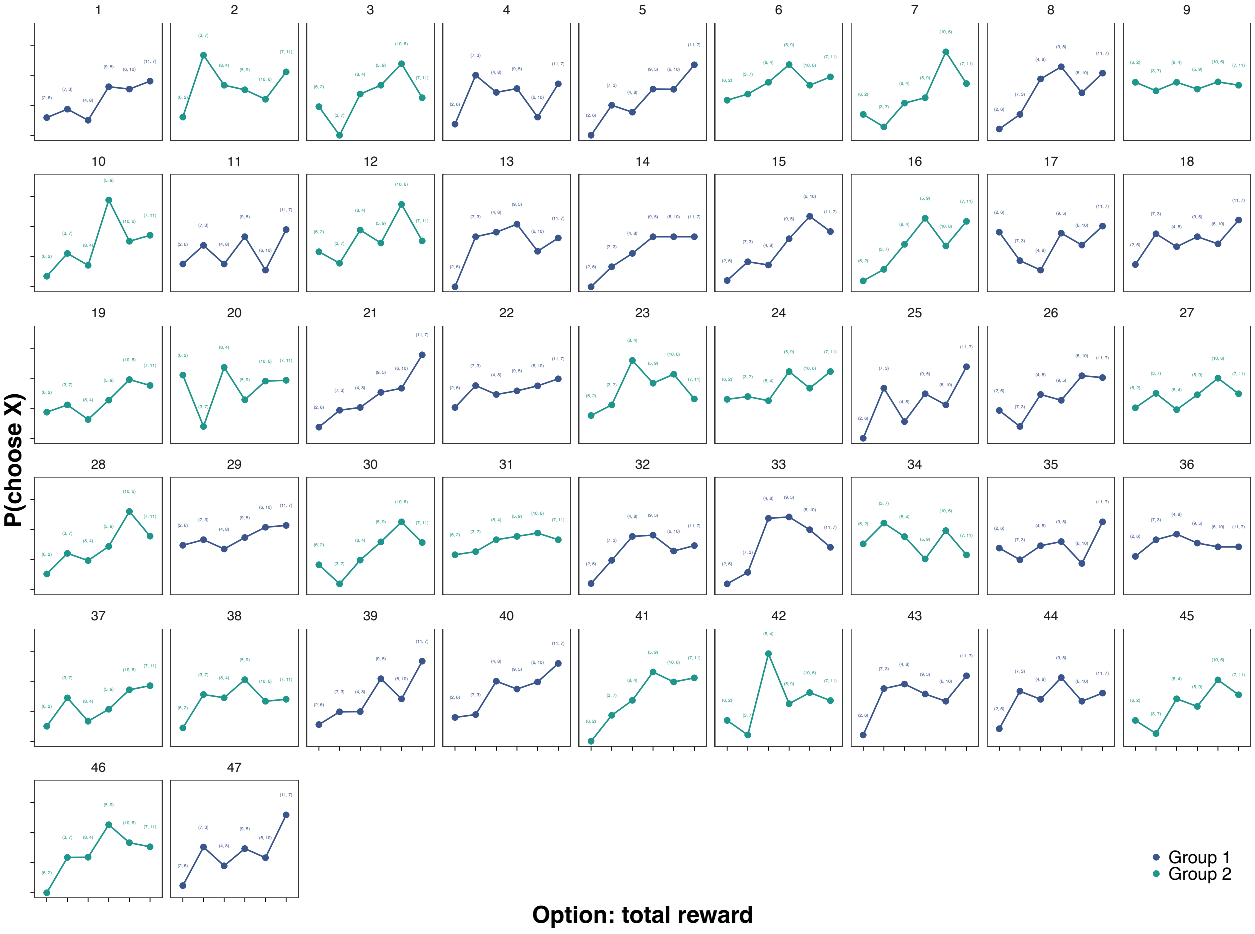

Figure D. Behavioral bias at the subject level for Study 4 In-lab Eye-tracking. Probability of choosing an option as a function of the option's total reward for Study 4 In-lab Eye-tracking.

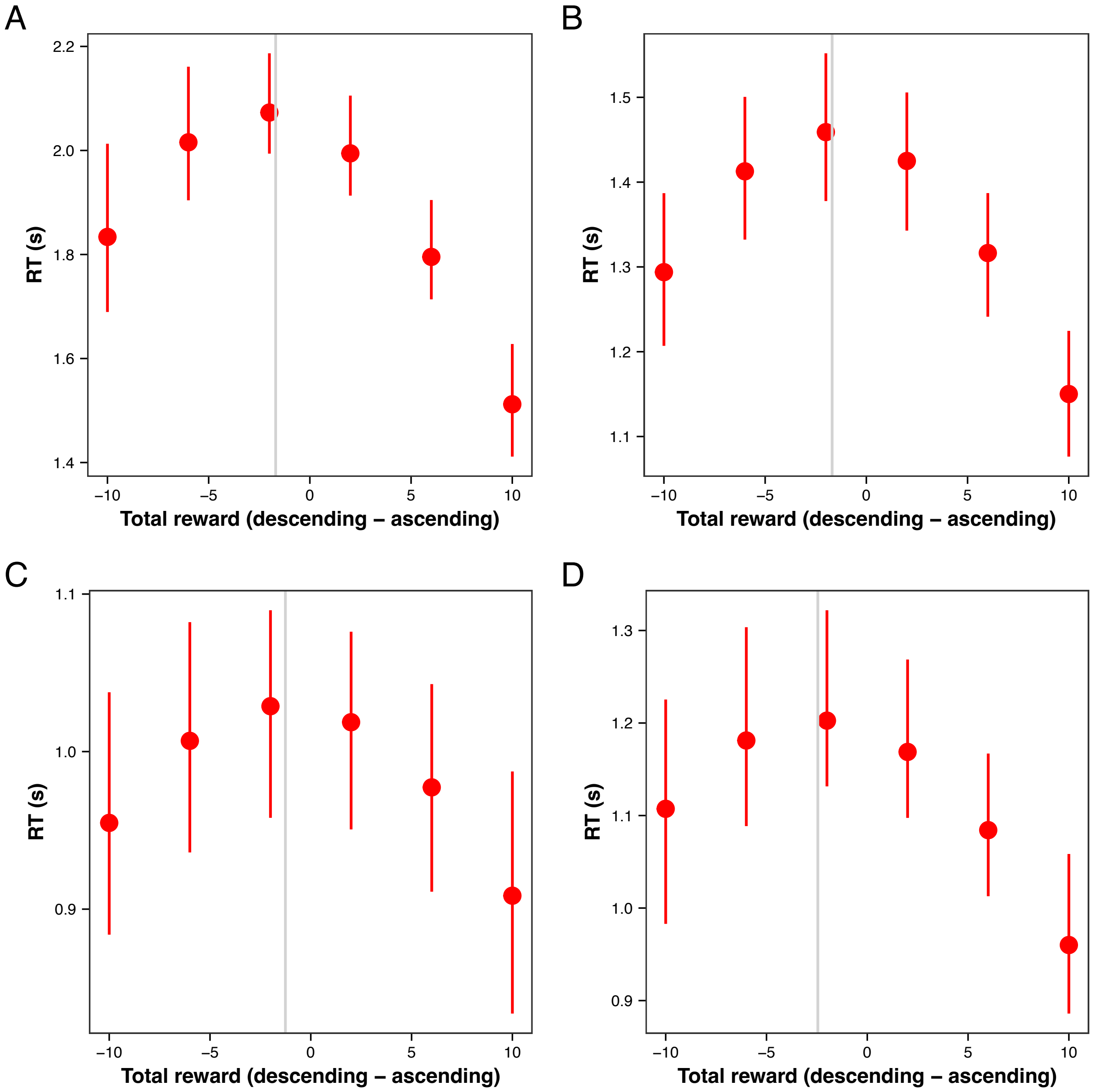

Figure E. RT indifference point. (A,B,C) Predicted RT in seconds from a mixed effects quadratic regression using difference in total underlying reward between right and left stimulus only for choice sets where one option is descending and the other is ascending. The red bars represent 95% confidence intervals. The gray line represents the predicted maximum RT. The indifference point occurs when the descending option is slightly worse than the ascending option. **(A)** Colors Task. **(B)** Patterns Task. **(A,B)** Feedback Position Condition: Immediate Reward: Top - Delayed Reward: Bottom. **(C)** Feedback Position Condition: Immediate Reward: Bottom - Delayed Reward: Top. **(D)** In-lab Eye-tracking.

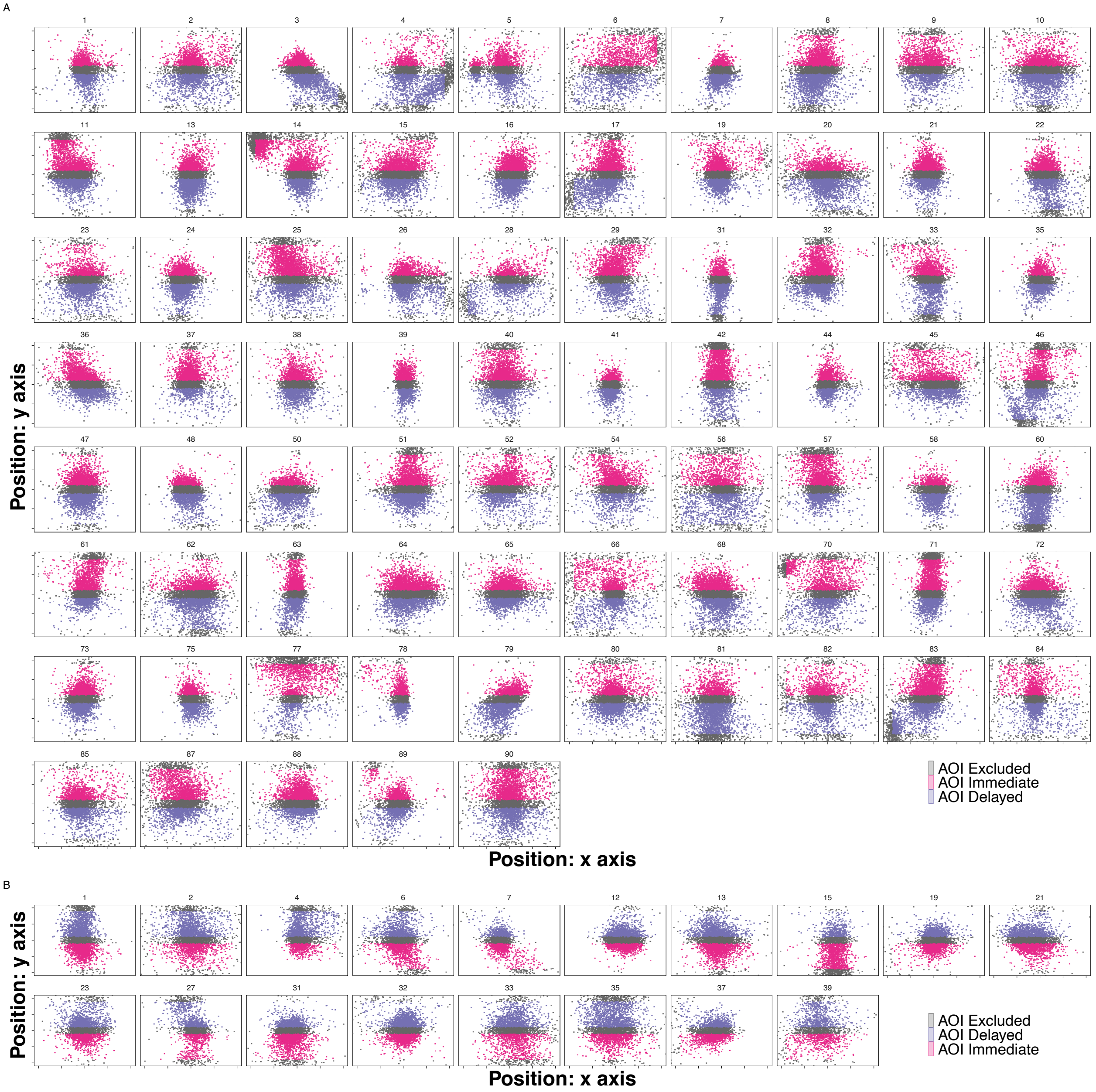

Figure F. Attention to feedback screen for each subject for Studies 2 and 3 Patterns. Online Patterns Study. **(A)** Feedback Position Condition: Immediate Reward: Top - Delayed Reward: Bottom. **(B)** Feedback Position Condition: Immediate Reward: Bottom - Delayed Reward: Top. Each dot represents one fixation.

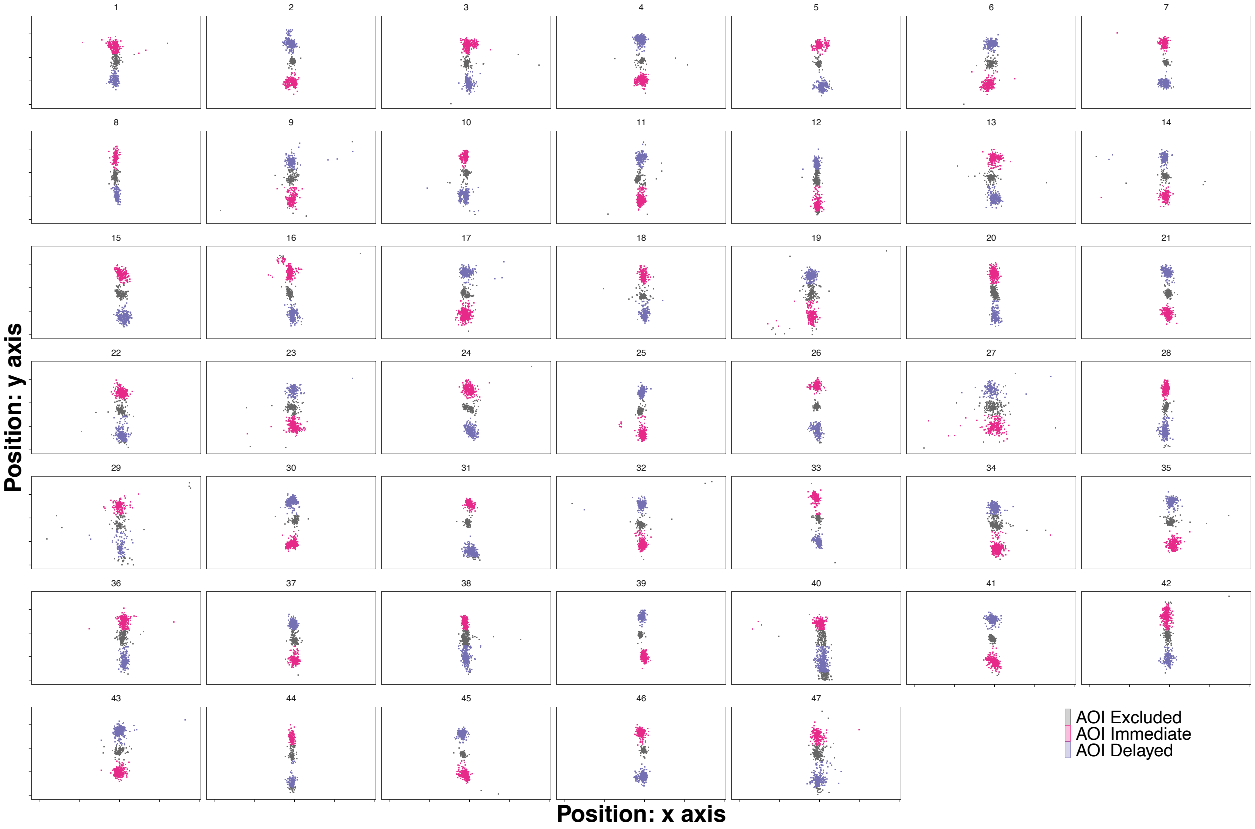

Figure G. Attention to feedback screen for each subject for Study 4 In-lab Eye-tracking. In-lab Eye-tracking Study. Each dot represents one fixation.

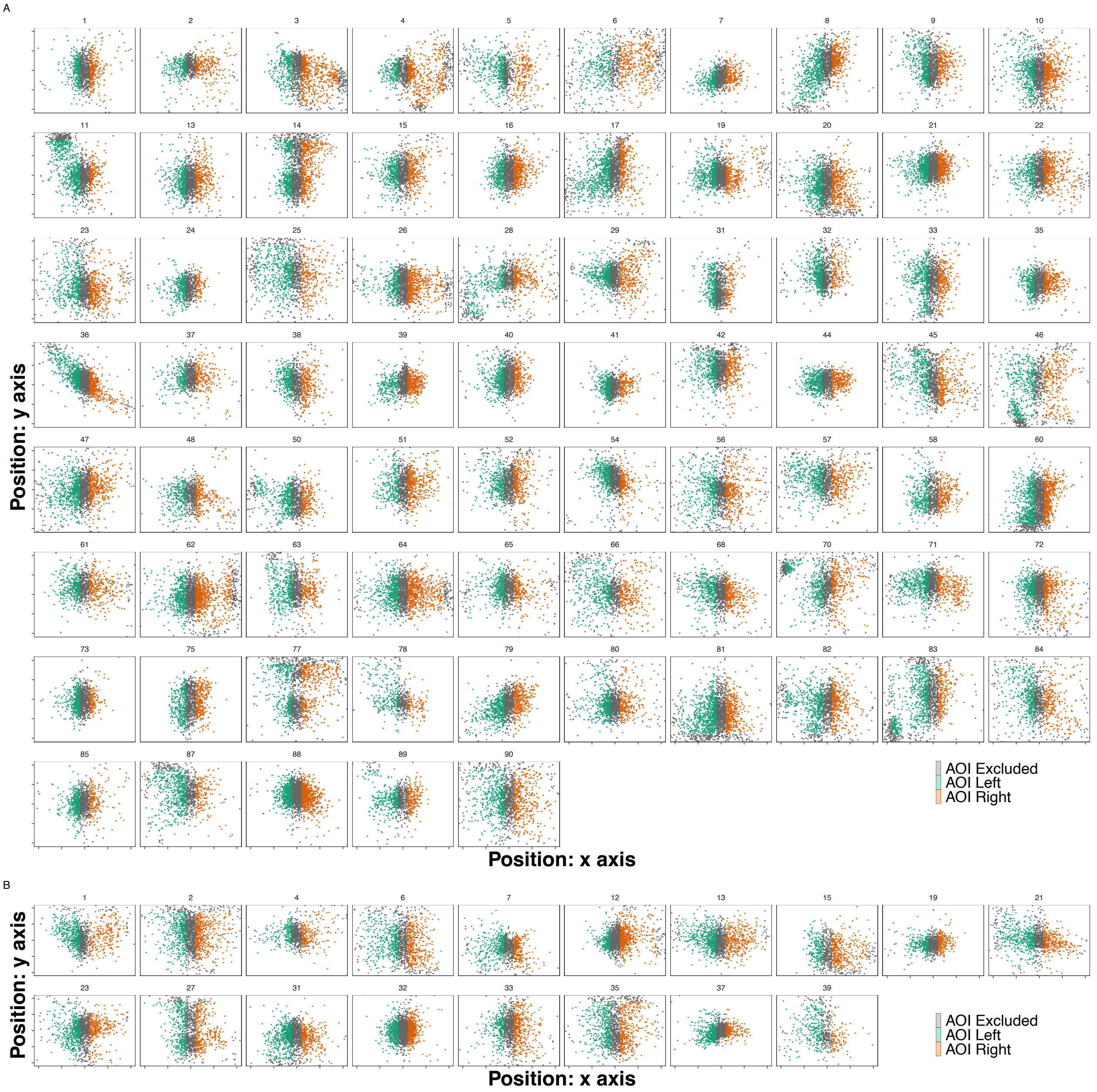

Figure H. Attention to the choice screen for each subject for Studies 2 and 3 Patterns. Online Patterns Study. **(A)** Feedback Position Condition: Immediate Reward: Top - Delayed Reward: Bottom. **(B)** Feedback Position Condition: Immediate Reward: Bottom - Delayed Reward: Top. Each dot represents one fixation.

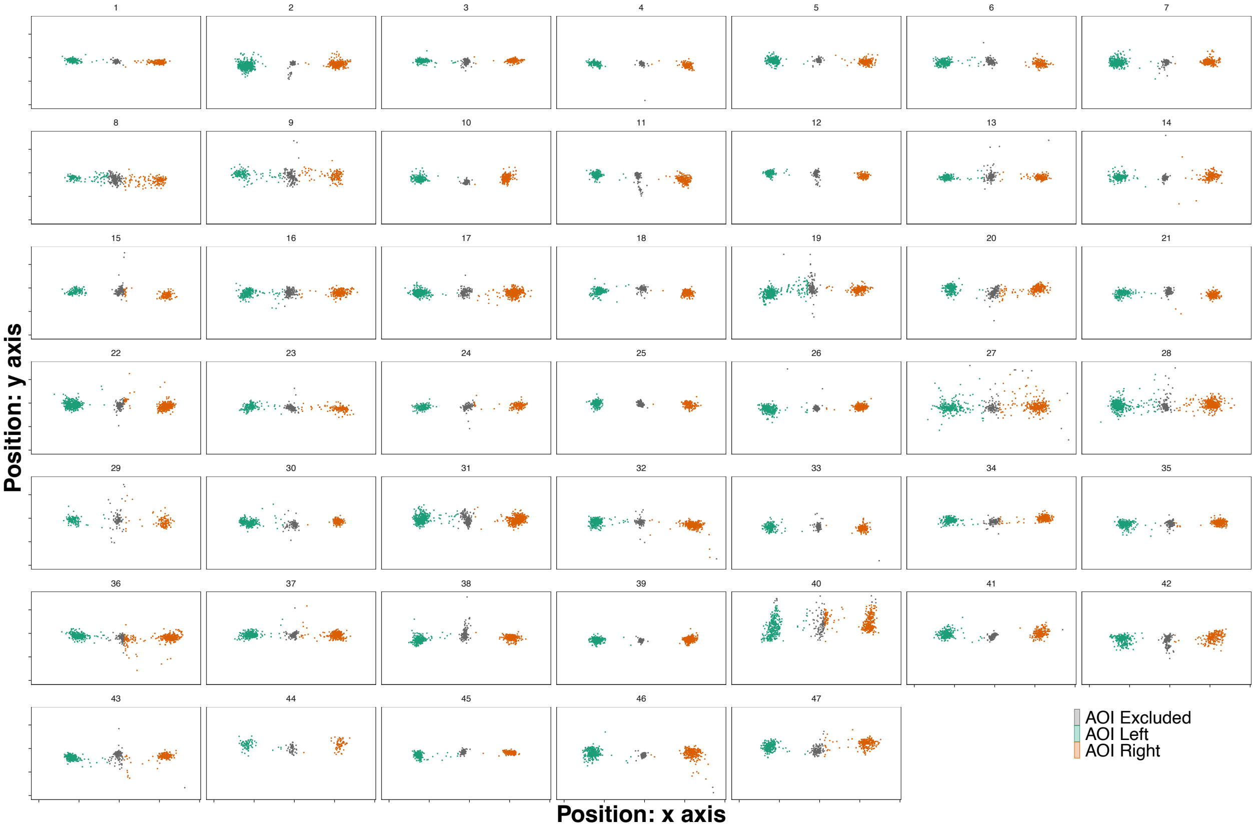

Figure I. Attention to the choice screen for each subject for Study 4 In-lab Eye-tracking. In-lab Eye-tracking Study. Each dot represents one fixation.

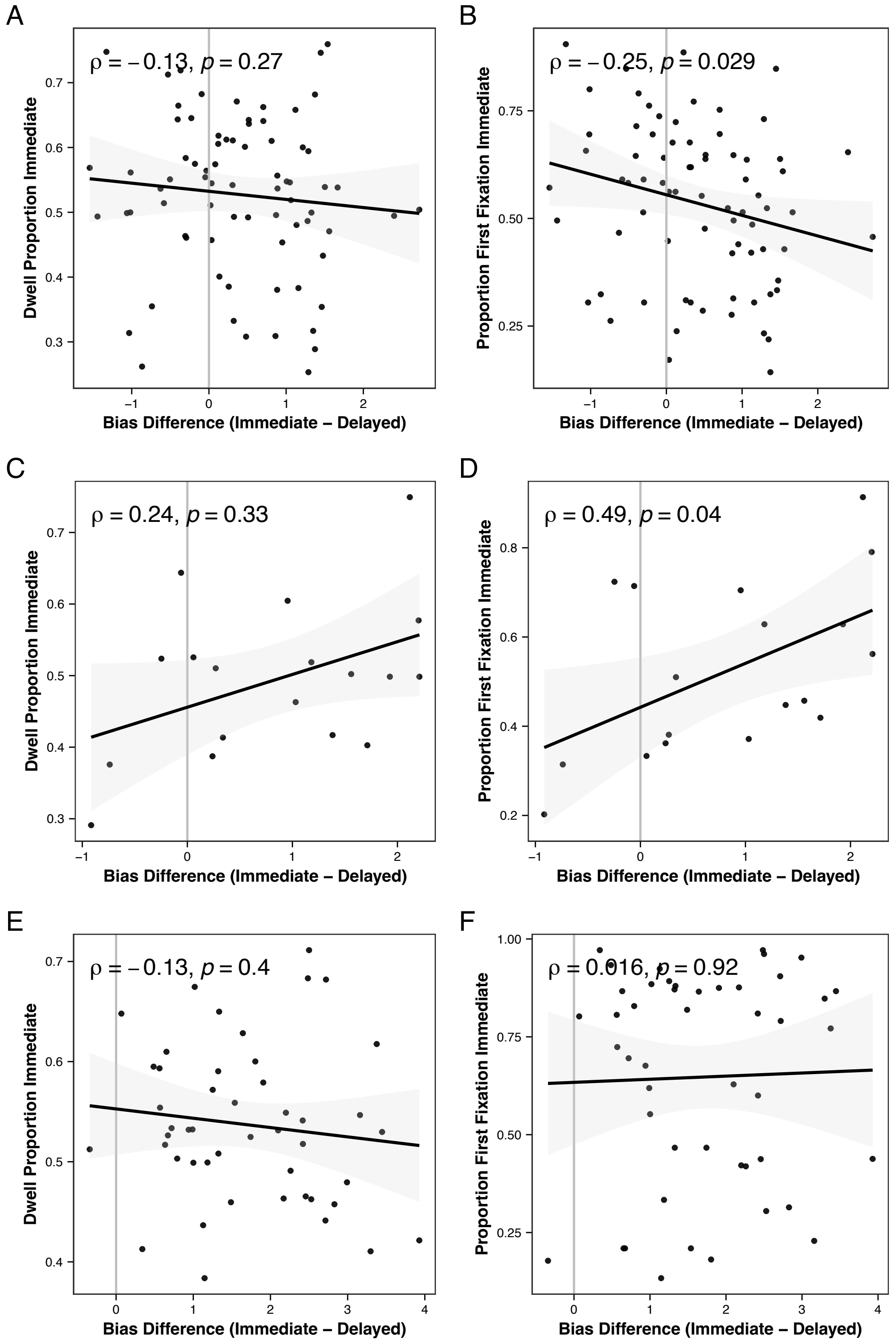

Figure J. Behavioral bias and attention. **(A,C,E)** Correlation between behavioral bias difference (the difference between coefficients in the experienced average immediate reward and delayed reward from mixed effects logistic regression of choosing the left stimulus) and average proportion of dwell time to the immediate reward across trials. **(B,D,F)** Correlation between behavioral bias difference and average proportion of first fixation to immediate rewards across trials. **(A,B)** Feedback Position Condition: Immediate Reward: Top - Delayed Reward: Bottom. **(C,D)** Feedback Position Condition: Immediate Reward: Bottom - Delayed Reward: Top. **(E,F)** In-lab Eye-tracking Study. Each dot represents a subject. The black line represents the best fitting linear regression line. The gray band represents the 95% CI.

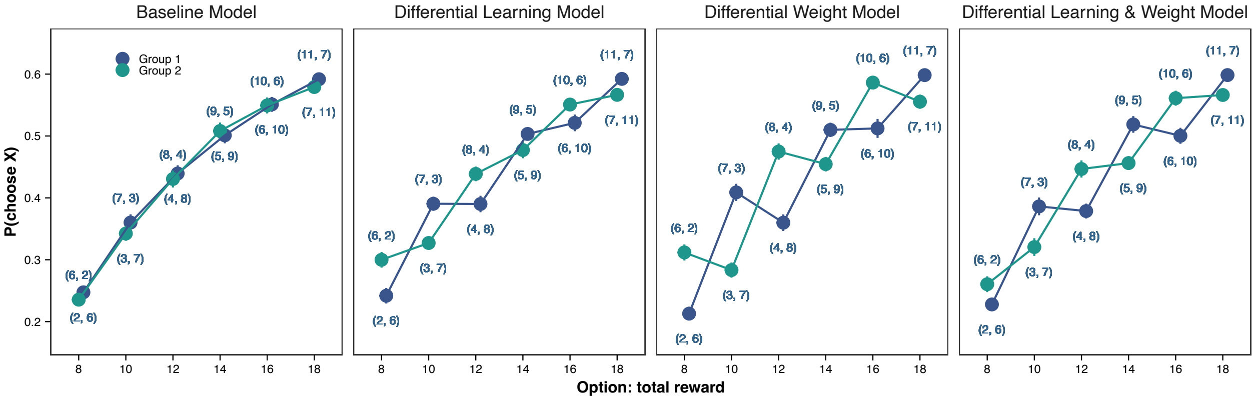

Figure K. Predicted aggregate behavioral bias. Predicted probability of choosing a stimulus given it is in the choice set as a function of the total reward of the stimulus for each condition. **(A)** For Baseline Model (same learning rate for immediate and delayed rewards). **(B)** For Differential Learning Model (different learning rates for immediate and delayed rewards). **(C)** Differential Weight Model (same learning rates for immediate and delayed rewards, weight on delayed reward). **(D)** For Differential Learning and Weight Model (different learning rates for immediate and delayed rewards and weight on delayed reward). The dots represent averages across the 100 simulated datasets for each subject in the Colors Task (Study 1) and Patterns Task (Study 2).

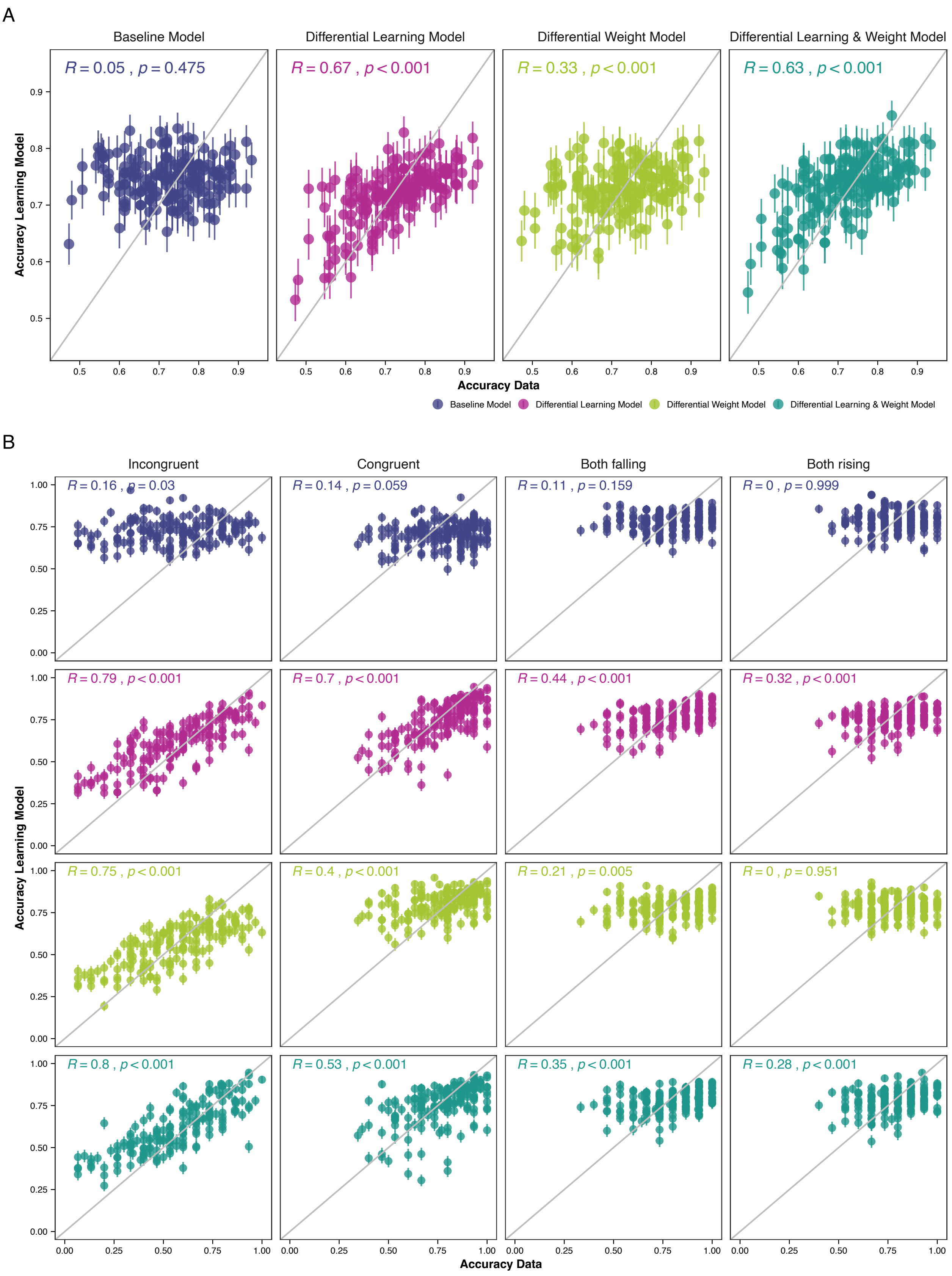

Figure L. Predicted and observed accuracy at the subject level. **(A)** Accuracy in data versus model. **(B)** Accuracy in

the data versus model. Incongruent choice sets: worse option descending, better option ascending, Congruent choice sets: worse option ascending, better option descending. The dots represent subjects. The bars represent standard errors of the 100 simulated datasets for each subject in the Colors Task (Study 1) and Patterns Task (Study 2).

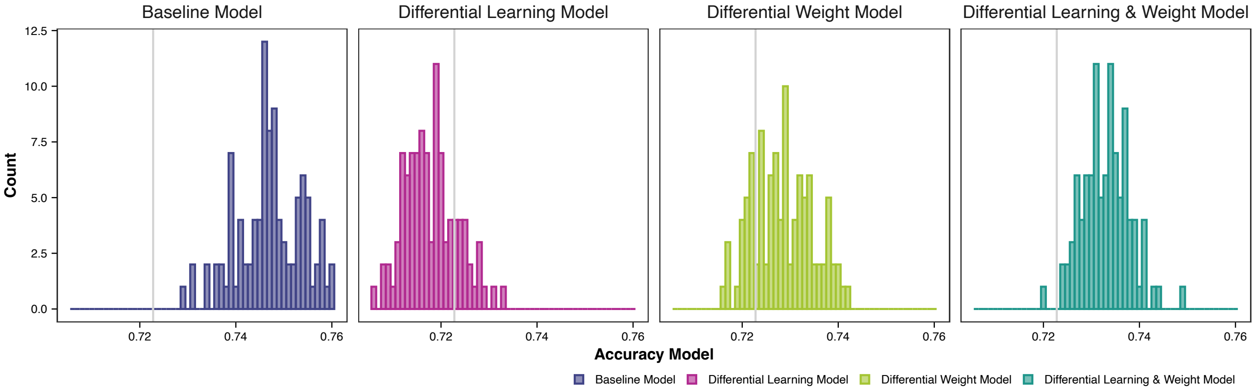

Figure M. Observed and predicted experiment accuracy. Histogram of model predicted mean accuracy across subjects in the Colors Task (Study 1) and Patterns Task (Study 2) for the 100 simulated datasets for each model. The gray line represents the mean accuracy across subjects in the data.

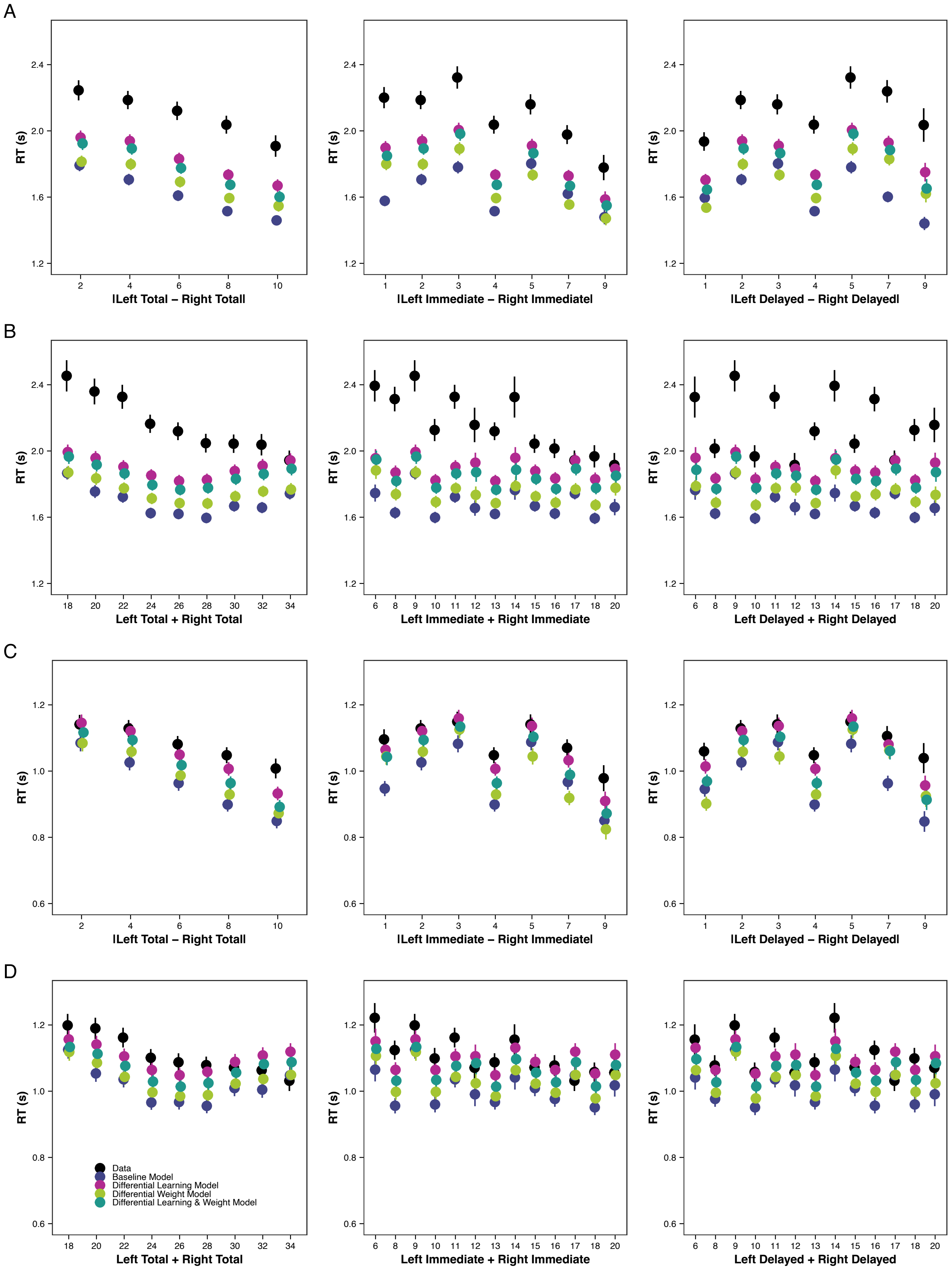

Figure N. Observed and predicted value difference (VD) and overall value (OV) effects on RT. (A,B) Colors Task

(Study 1). **(C,D)** Patterns Task (Study 2). **(A,C)** VD. **(B,D)** OV. The model data was based on the 100 simulated datasets for each subject in the Colors Task (Study 1) and Patterns Task (Study 2).

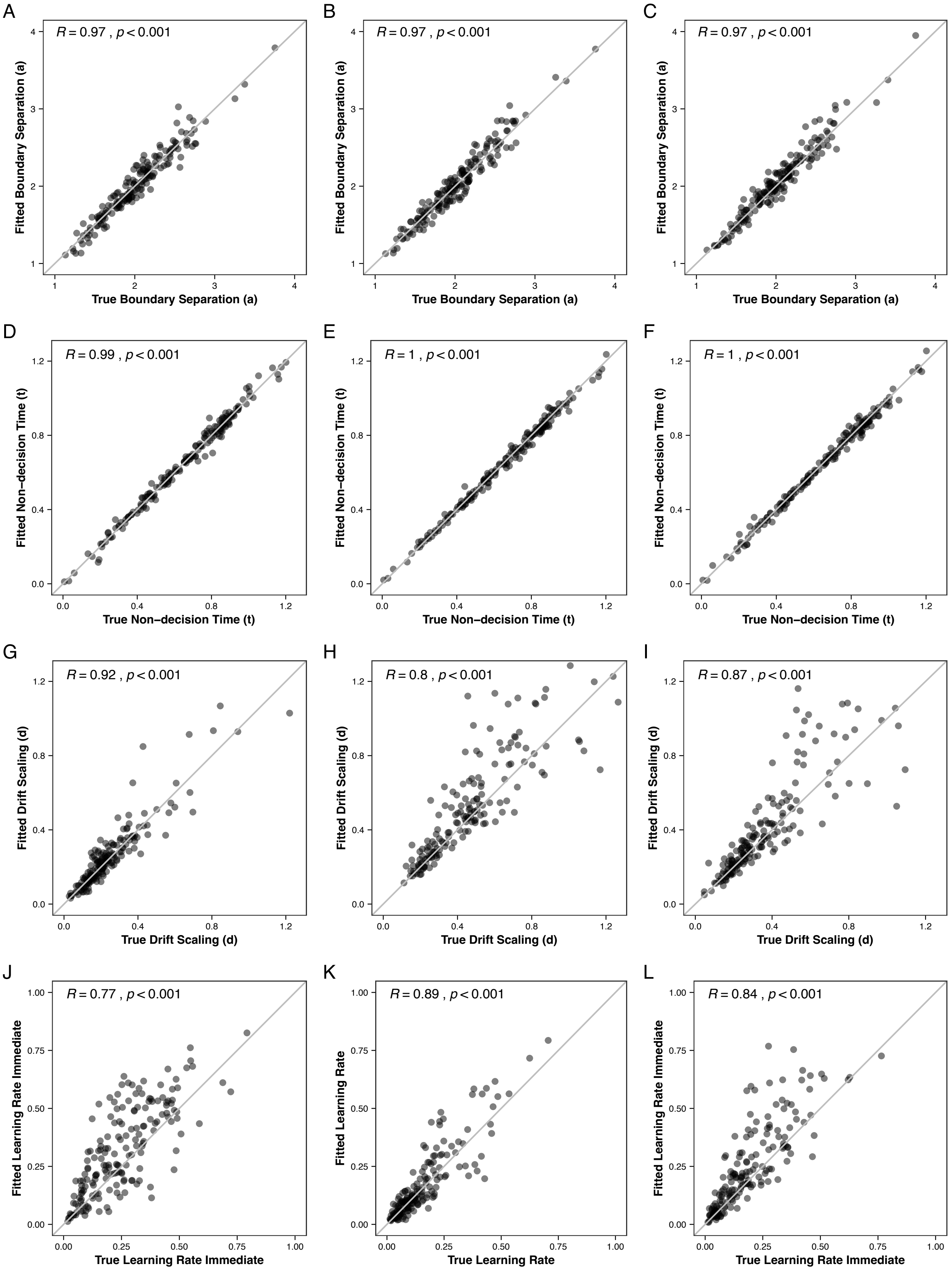
A
R = 0.97 , p < 0.001
Fitted Boundary Separation (a)
True Boundary Separation (a)
B
R = 0.97 , p < 0.001
Fitted Boundary Separation (a)
True Boundary Separation (a)
C
R = 0.97 , p < 0.001
Fitted Boundary Separation (a)
True Boundary Separation (a)
D
R = 0.99 , p < 0.001
Fitted Non−decision Time (t)
True Non−decision Time (t)
E
R = 1 , p < 0.001
Fitted Non−decision Time (t)
True Non−decision Time (t)
F
R = 1 , p < 0.001
Fitted Non−decision Time (t)
True Non−decision Time (t)
G
R = 0.92 , p < 0.001
Fitted Drift Scaling (d)
True Drift Scaling (d)
H
R = 0.8 , p < 0.001
Fitted Drift Scaling (d)
True Drift Scaling (d)
I
R = 0.87 , p < 0.001
Fitted Drift Scaling (d)
True Drift Scaling (d)
J
R = 0.77 , p < 0.001
Fitted Learning Rate Immediate
True Learning Rate Immediate
K
R = 0.89 , p < 0.001
Fitted Learning Rate
True Learning Rate
L
R = 0.84 , p < 0.001
Fitted Learning Rate Immediate
True Learning Rate Immediate

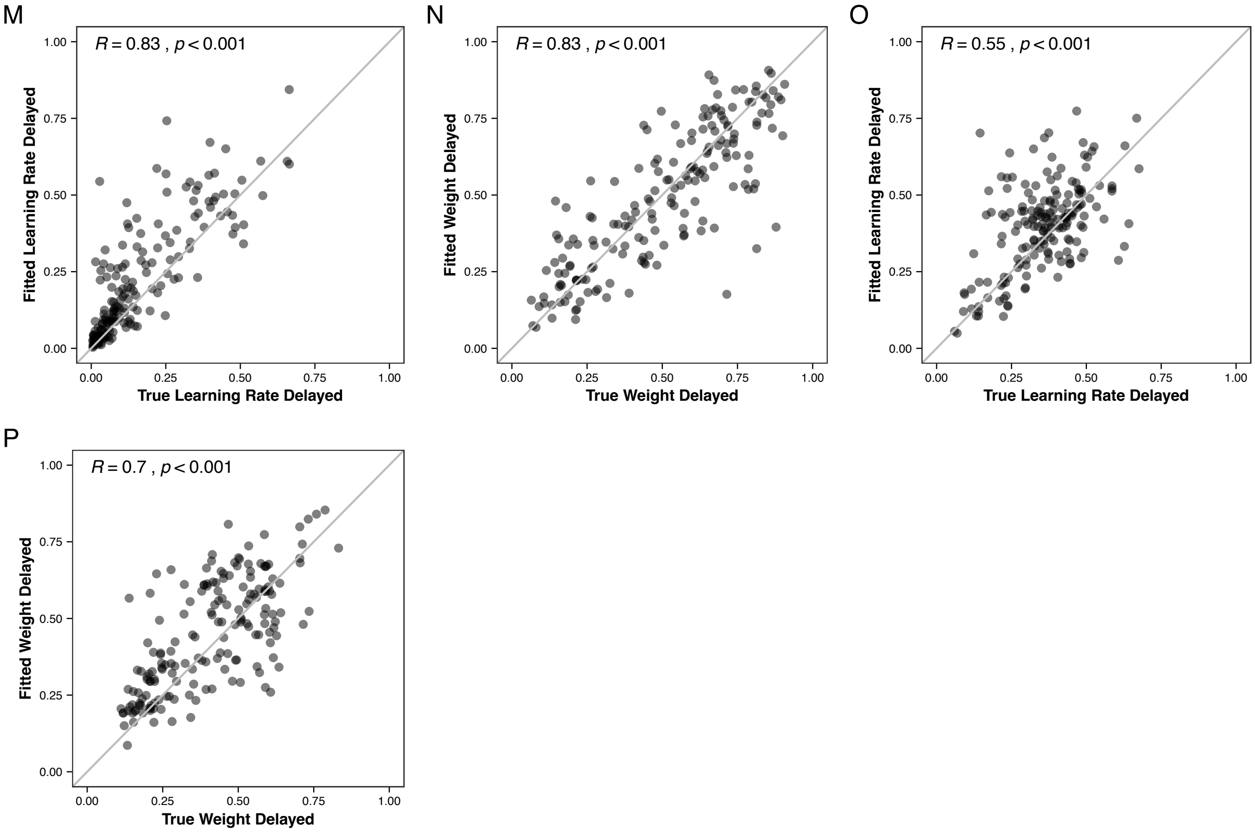

Figure O. RL parameters recovery. Correlations between true parameter value used to generate the data and the mean posterior of the parameter values for the Differential Learning Model. Each dot represents a simulated subject. **(A,B,C)** Boundary separation. **(D,E,F)** Non-decision time. **(G,H,I)** Drift scaling parameter. **(K)** Learning rate parameter. **(J,L)** Immediate learning rate. **(M,O)** Delayed learning rate. **(N,P)** Weight parameter. **(A,D,G,J,M)** Differential Learning Model. **(B,E,H,K,N)** Differential Weight Model. **(C,F,I,L,O,P)** Differential Learning and Weight Model.

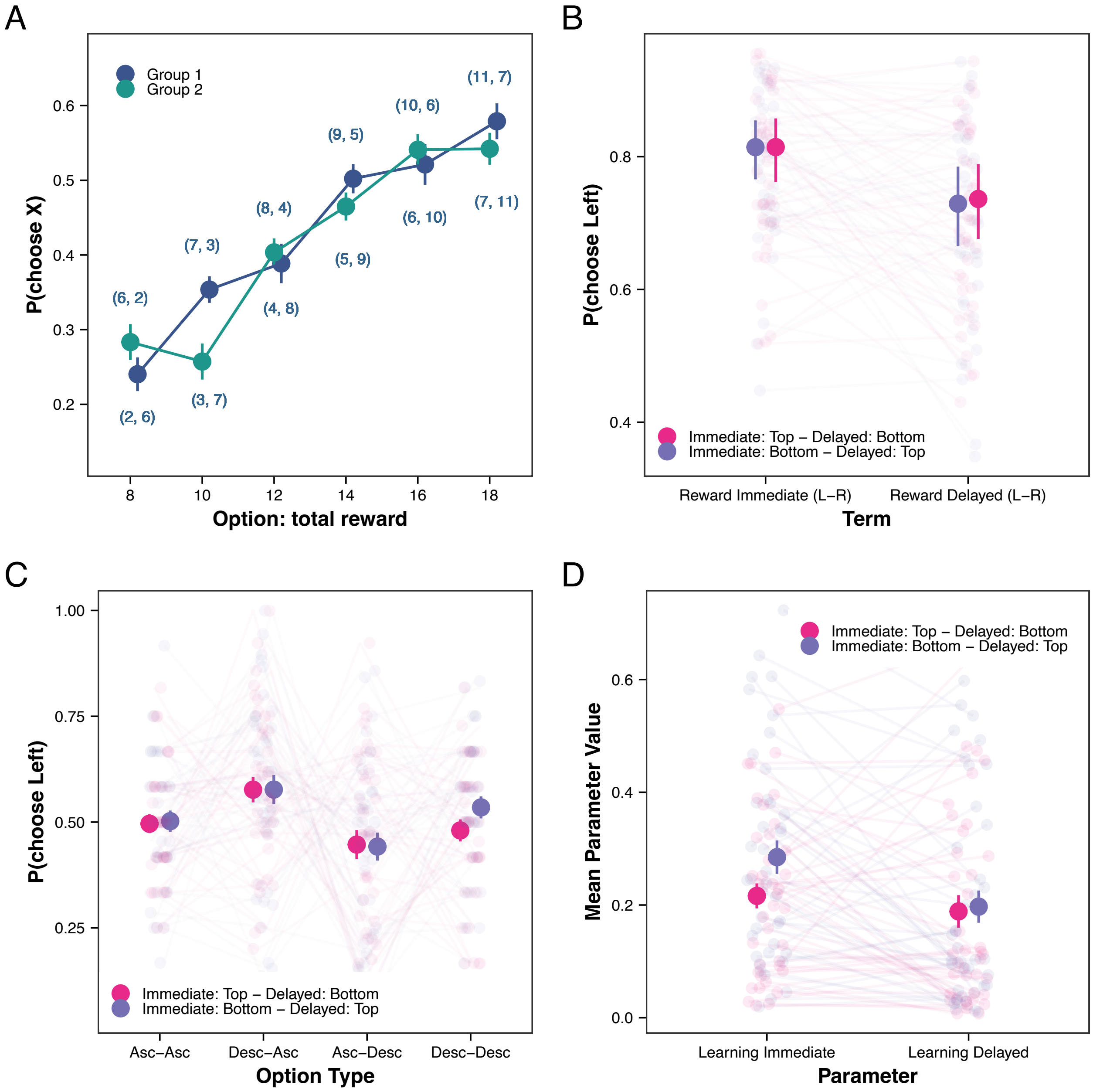

Figure P. Behavioral bias for reversed feedback position. **(A)** Probability of choosing a stimulus given it is in the choice set as a function of the total reward of the stimulus. For the same total value of the option, the descending option is more likely to be chosen than the ascending option. **(B)** Probability of choosing the left stimulus as a function of the difference between the left and right option in the experienced immediate reward and delayed reward, based on a mixed-effects logistic regression. Dots represent subject level effects and bars represent standard errors of the fixed effects. **(C)** Probability of choosing the left stimulus as a function of whether the left option was descending or ascending. Dots represent subject level averages and bars represent standard errors across subjects. **(D)** Mean and standard errors of posteriors of immediate and delayed learning rates. Dots represent subject level learning rates for each task.

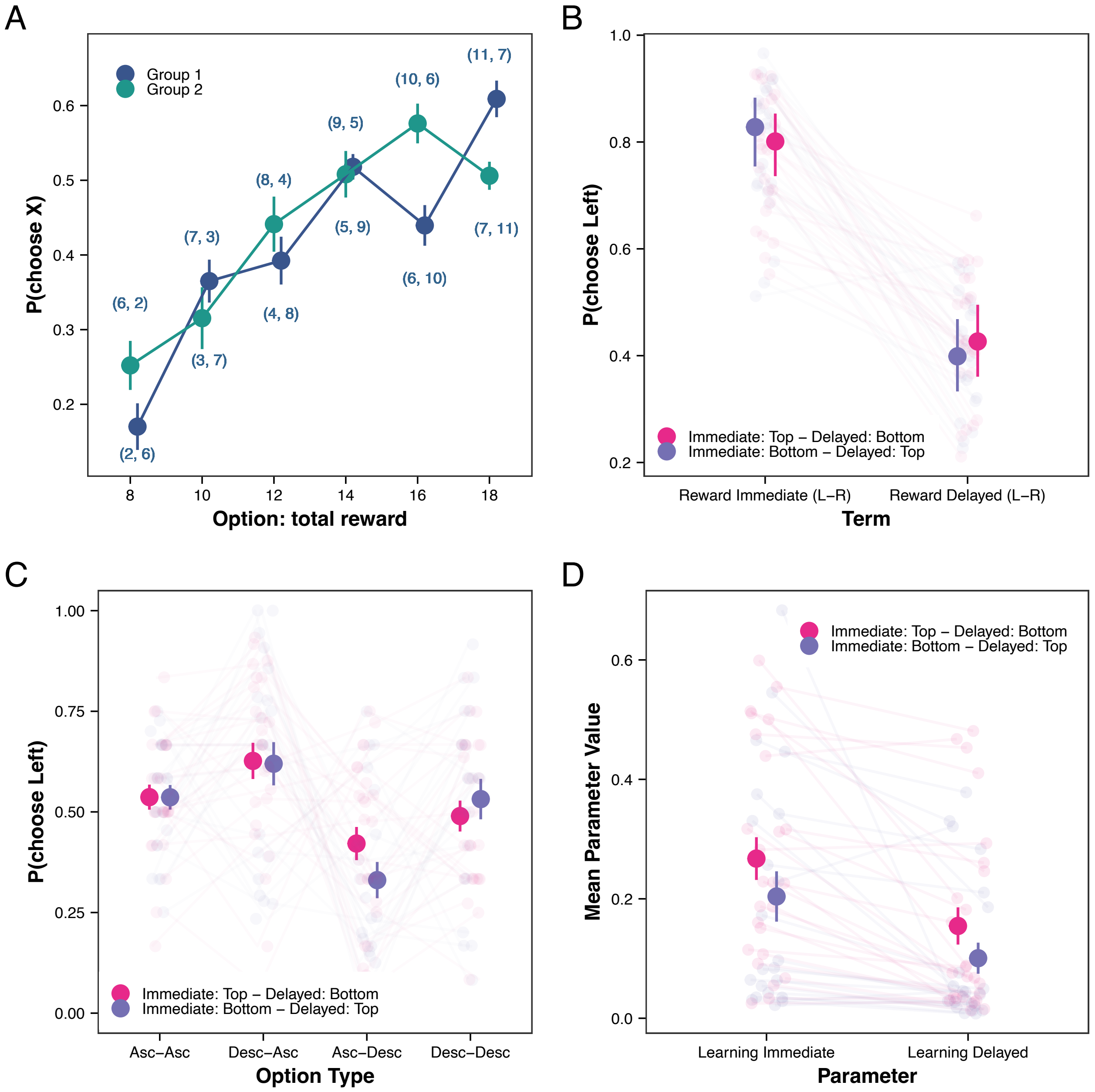

Figure Q. Behavioral bias for in-lab eye-tracking study. **(A)** Probability of choosing a stimulus given it is in the choice set as a function of the total reward of the stimulus. For the same total value of the option, the descending option is more likely to be chosen than the ascending option. **(B)** Probability of choosing the left stimulus as a function of the difference between the left and right option in the experienced immediate reward and delayed reward, based on a mixed-effects logistic regression. Dots represent subject level effects and bars represent standard errors of the fixed effects. **(C)** Probability of choosing the left stimulus as a function of whether the left option was descending or ascending. Dots represent subject level averages and bars represent standard errors across subjects. **(D)** Mean and standard errors of posteriors of immediate and delayed learning rates. Dots represent subject level learning rates for each task.

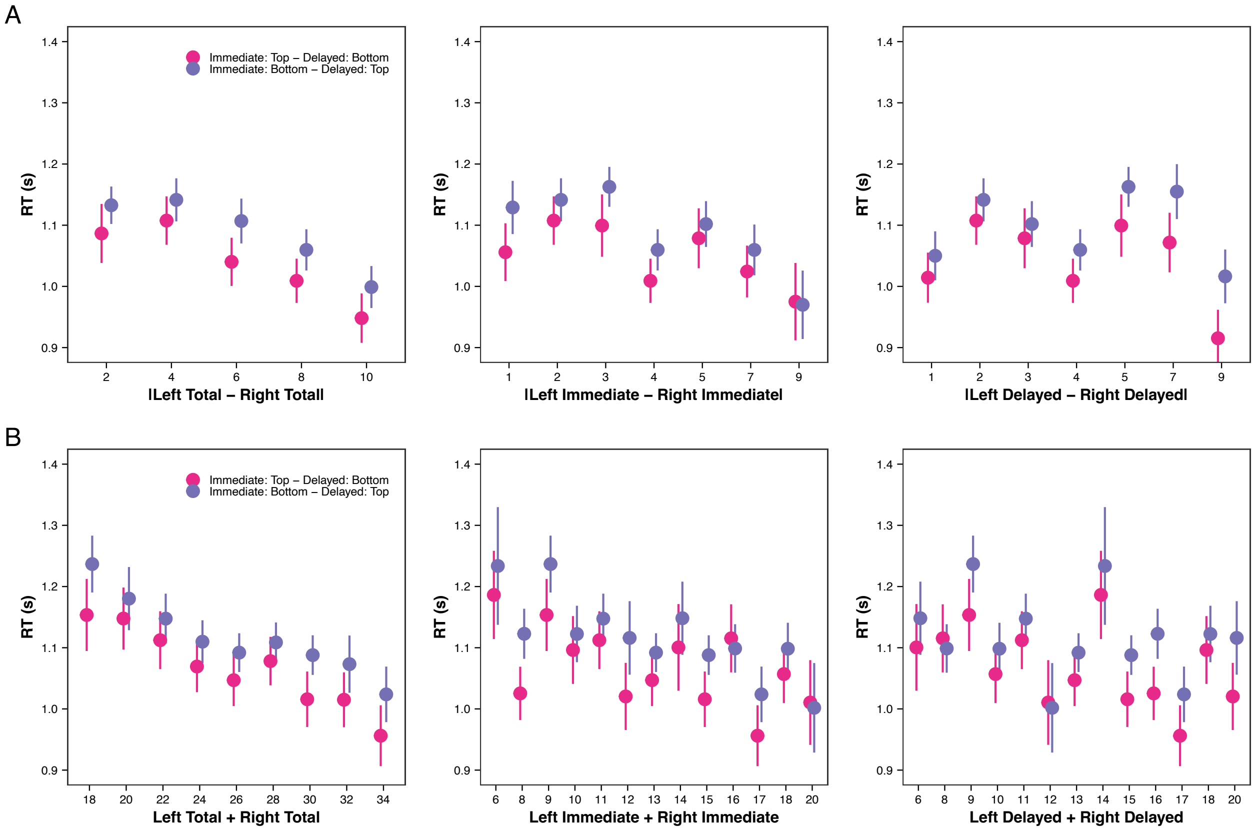

Figure R. Value difference (VD) and overall value (OV) effects on response times (RT) for reversed feedback position condition. (A) Absolute value difference (|VD|) effects on RT for each Study. RT decreases with total |VD|. RT decreases with immediate |VD|. RT does not decrease with delayed |VD|. **(B)** Overall value (OV) effects on RT for each study. RT decreases with total OV. RT decreases with immediate OV. RT weakly decreases with delayed OV. Dots and bars represent mean and standard errors across subjects for each task.

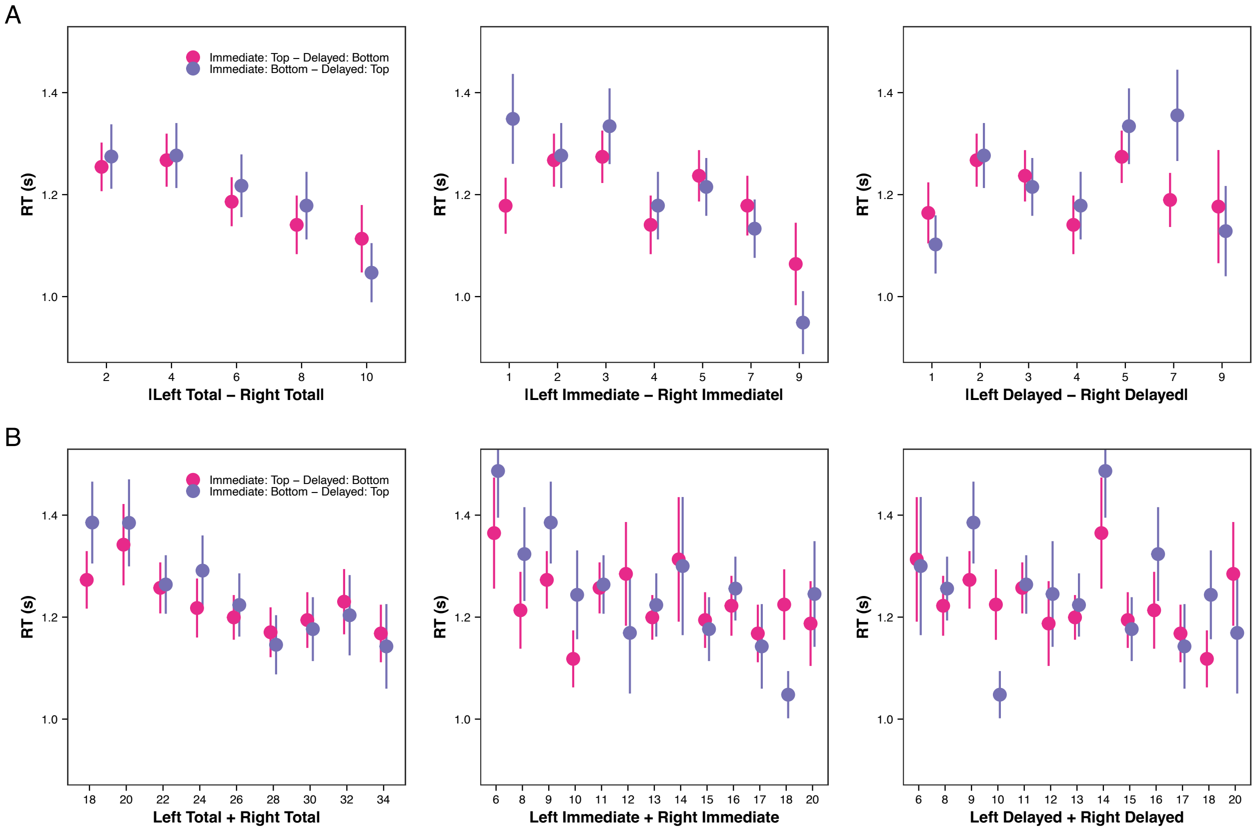

Figure S. Value difference (VD) and overall value (OV) effects on response times (RT) for in-lab eye-tracking study. (A) Absolute value difference (|VD|) effects on RT for each Study. RT decreases with total |VD|. RT decreases with immediate |VD|. RT does not decrease with delayed |VD|. **(B)** Overall value (OV) effects on RT for each study. RT decreases with total OV. RT decreases with immediate OV. RT weakly decreases with delayed OV. Dots and bars represent mean and standard errors across subjects for each task.

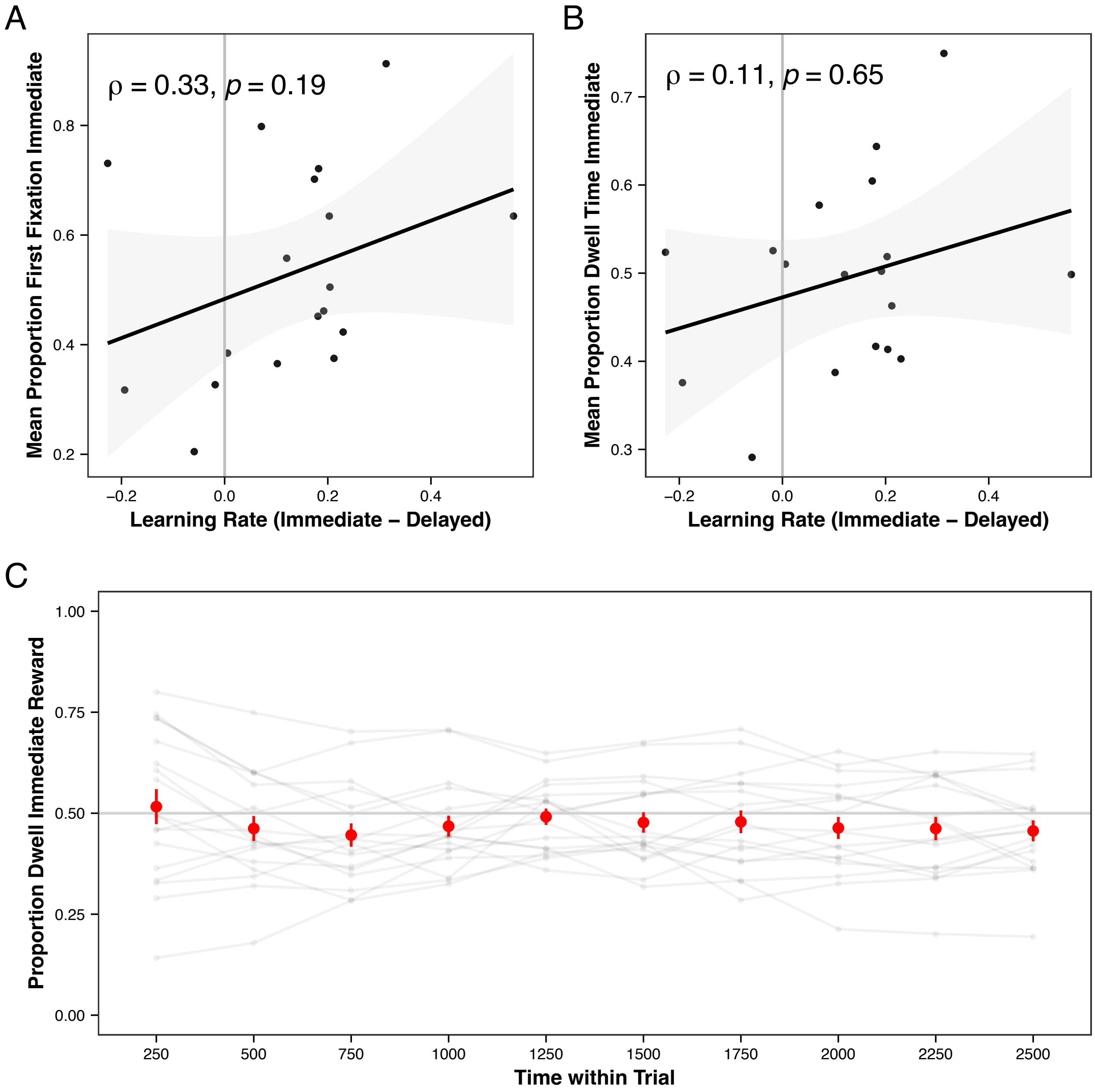

Figure T. Attention and behavioral bias for reversed feedback position. **(A)** Correlation between difference in learning rate between immediate and delayed reward and proportion of first fixation to the immediate reward. **(B)** Correlation between difference in learning rate between immediate and delayed reward and mean dwell proportion immediate reward across trials. **(A,B)** Dots represent each subject. The black line represents the best fitting linear regression line. The gray band represents the 95% CI. **(C)** Mean and standard errors of dwell proportion to immediate reward within a trial for each time bin. Black dots represent each subject within a time bin.

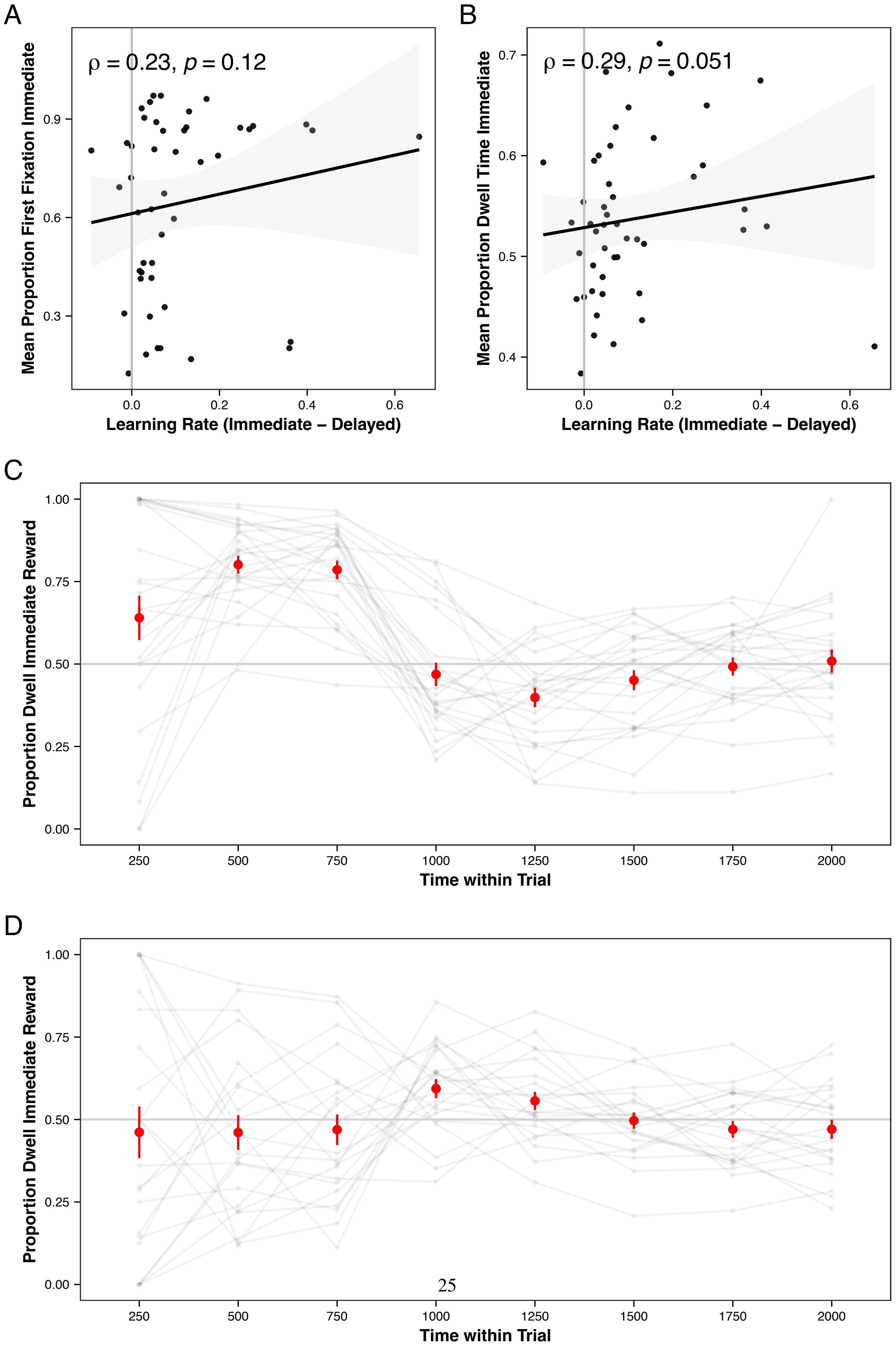
A
ρ = 0.23, p = 0.12
Mean Proportion First Fixation Immediate
Learning Rate (Immediate − Delayed)
B
ρ = 0.29, p = 0.051
Mean Proportion Dwell Time Immediate
Learning Rate (Immediate − Delayed)
C
Proportion Dwell Immediate Reward
Time within Trial
D
Proportion Dwell Immediate Reward
Time within Trial

Figure U. Attention and behavioral bias for in-lab eye-tracking data. **(A)** Correlation between difference in learning rate between immediate and delayed reward and proportion of first fixation to the immediate reward. **(B)** Correlation between difference in learning rate between immediate and delayed reward and mean dwell proportion immediate reward across trials. **(A,B)** Dots represent each subject. The black line represents the best fitting linear regression line. The gray band represents the 95% CI. **(C,D)** Mean and standard errors of dwell proportion to immediate reward within a trial for each time bin. **(C)** Feedback Position Condition: Immediate Reward: Top - Delayed Reward: Bottom. **(D)** Feedback Position Condition: Immediate Reward: Bottom - Delayed Reward: Top. Black dots represent each subject within a time bin.

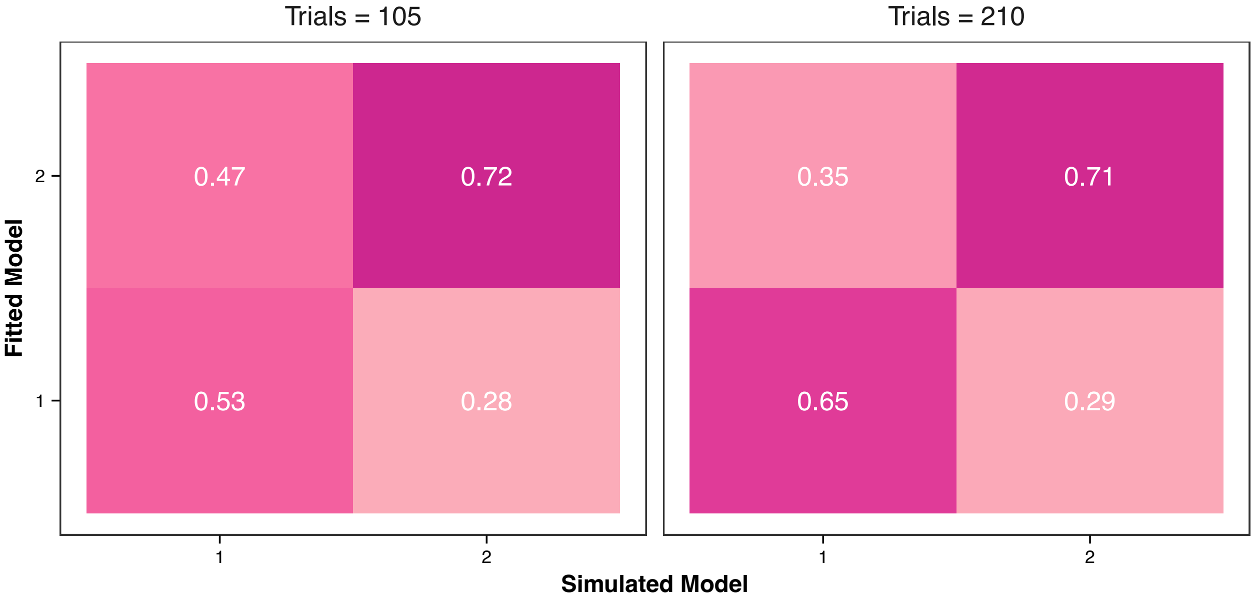

Figure V. Model recovery. Percent of subjects better fit by the data generating model versus the alternative model using WAIC for datasets with 105 or 210 trials. Model 1: Differential Learning Model. Model 2: Differential Weight Model

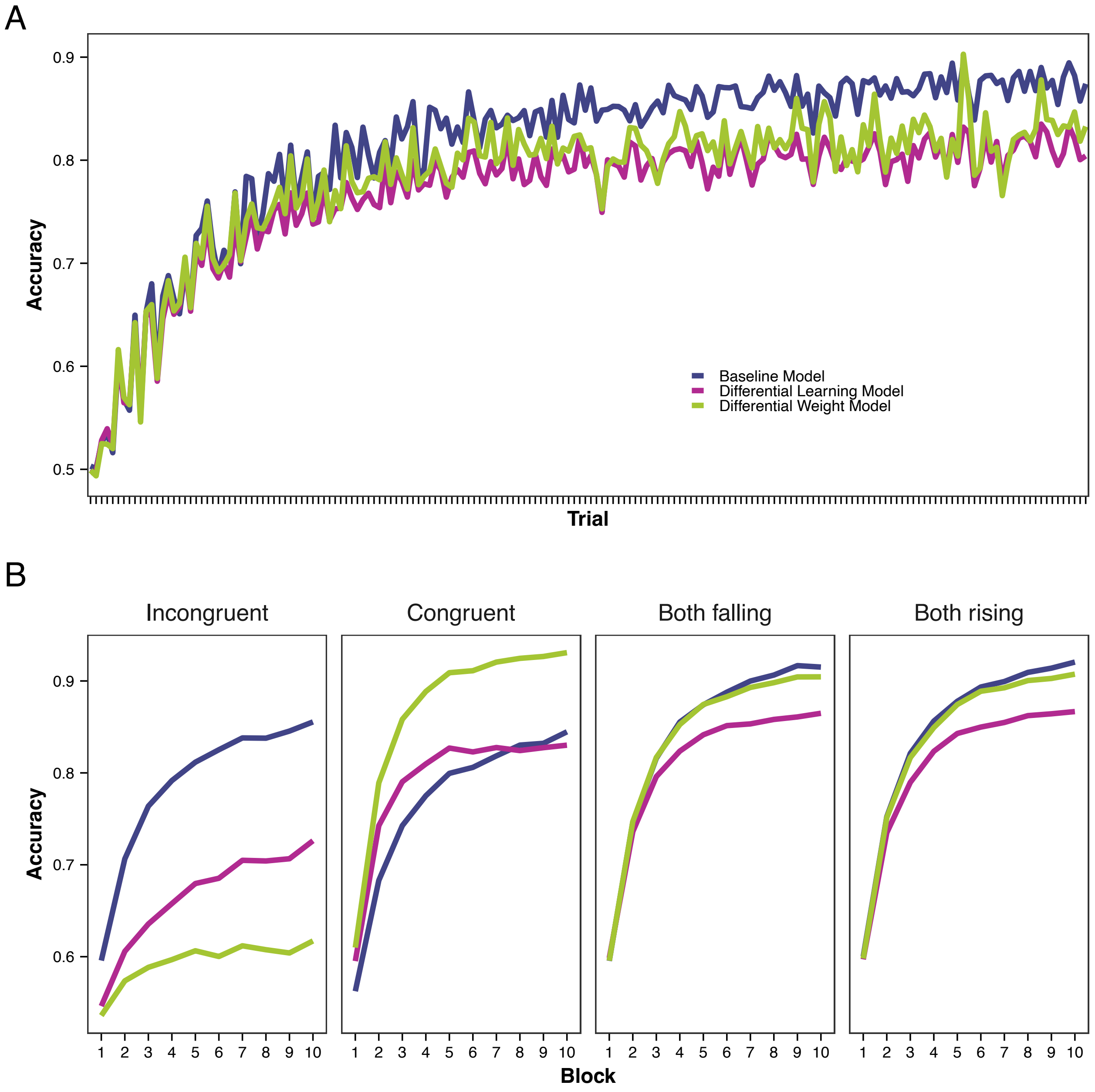

Figure W. Model simulations with double the number of trials compared to the experiment. (A,B) Average choice accuracy in the model simulations using the mean posterior values across trials and subjects. **(A)** Experiment level. **(B)** Block level for each type of trial. Incongruent choice sets: worse option descending, better option ascending, Congruent choice sets: worse option ascending, better option descending. The model simulations is based 100 simulated datasets using the fitted parameter values for each subject in the Colors Task (Study 1) and Patterns Task (Study 2).

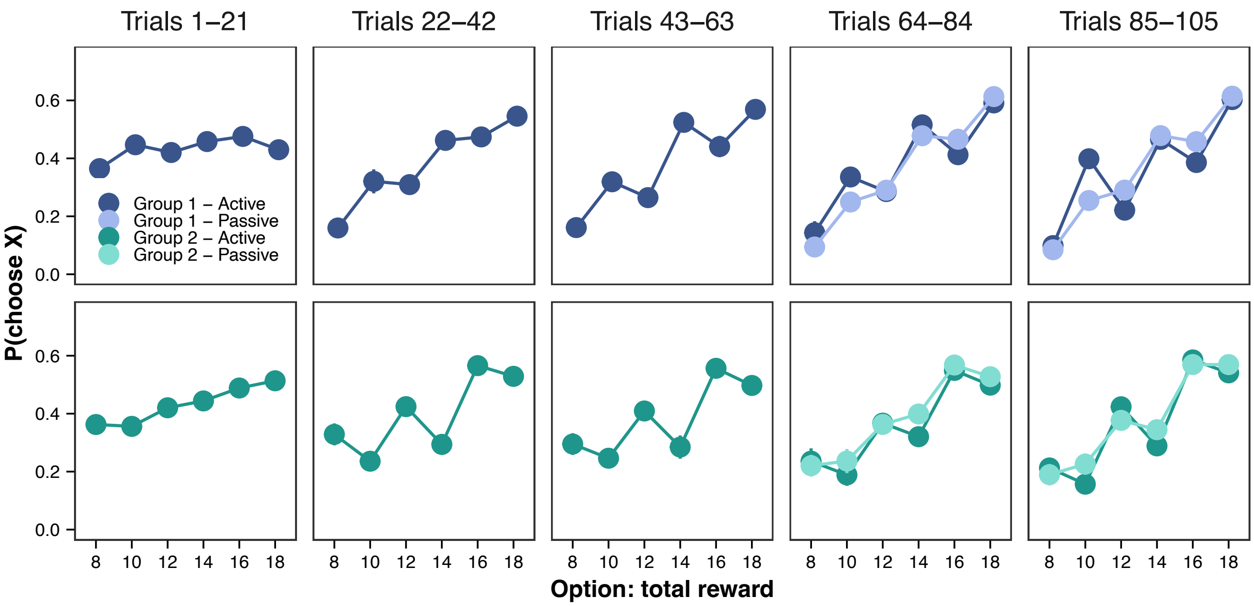

Figure X. Behavioral bias over time in the passive learning condition. Probability of choosing a stimulus given it is in the choice set as a function of the total reward of the stimulus. In the first 63 trials, subjects did not make any decisions, but could learn passively from feedback shown on their screen. The feedback shown was based on the decisions of a matched partner from the main experiment. The feedback and timing of the feedback was the same as that of the matched partner, except that the subject could not see the foregone choice option on the choice screen. After trial 64, subjects made decisions. Active condition data is based on the matched subjects. Dots represent subject level averages and bars represent standard errors across subjects.

Supplementary Tables

	Choice (Left)				
	(patterns)	(colors)	(both)	(colors)	(passive)
	(1)	(2)	(3)	(4)	(5)
Right Desc	-0.49^{***}	-1.35^{***}	-0.50^{***}	-1.75^{***}	-0.53^{***}
	(0.12)	(0.17)	(0.14)	(0.22)	(0.13)
Left Desc	0.30^{*}	1.16^{***}	0.31^{*}	1.33^{***}	0.86^{***}
	(0.12)	(0.15)	(0.13)	(0.20)	(0.13)
Total (L-R)	1.73^{***}	2.04^{***}	1.72^{***}	2.35^{***}	1.67^{***}
	(0.11)	(0.10)	(0.10)	(0.18)	(0.08)
Condition (Colors)			-0.06		
			(0.11)		
Right Desc:Condition			-0.84^{***}		
			(0.20)		
Left Desc:Condition			0.84^{***}		
			(0.19)		
Total (L-R):Condition			0.34^{*}		
			(0.15)		
Passive				-0.17	
				(0.19)	
Right Desc:Passive				0.73^{*}	
				(0.35)	
Left Desc:Passive				-0.23	
				(0.33)	
Total (L-R):Passive				0.33	
				(0.29)	
Constant	0.12	0.06	0.12	0.23	-0.12
	(0.08)	(0.08)	(0.08)	(0.13)	(0.12)
Observations	5,358	5,219	10,577	4,769	1,710
Log Likelihood	-2,631.18	-2,189.50	-4,827.39	-1,955.14	-807.41
Akaike Inf. Crit.	5,290.35	4,407.00	9,690.77	3,946.29	1,624.83
Bayesian Inf. Crit.	5,382.56	4,498.84	9,821.57	4,062.74	1,652.05
Note:	$^{*}p<0.05$; $^{**}p<0.01$; $^{***}p<0.001$				

Table A. Type of option effect on choice. Logistic regression of choice of left stimulus on whether the right and left stimuli are descending or ascending controlling for difference in mean experienced total reward between left and right stimuli. (1) Patterns Task. (2) Colors Task. (3) Patterns and Colors Tasks. (4) Colors Passive and Active Conditions. (5) Colors Passive Condition. Continuous variables are z-scored. Regressions include random intercepts and random slopes for whether the right and left stimuli are descending or ascending and for difference in mean experienced total reward at the subject level.

	Choice (Left)							
	(patterns)	(colors)	(both)	(patterns)	(colors)	(both)	(colors)	(passive)
	(1)	(2)	(3)	(4)	(5)	(6)	(7)	(8)
Immediate (L-R)	1.54***	2.18***	1.53***	1.59***	2.24***	1.57***	2.56***	2.62***
	(0.11)	(0.11)	(0.10)	(0.11)	(0.11)	(0.10)	(0.18)	(0.26)
Delayed (L-R)	1.11***	0.90***	1.09***	1.14***	0.92***	1.12***	1.00***	1.48***
	(0.10)	(0.10)	(0.10)	(0.10)	(0.10)	(0.10)	(0.14)	(0.21)
Trial				0.03	0.01	0.02		
				(0.04)	(0.04)	(0.04)		
Immediate (L-R):Trial				0.34***	0.38***	0.34***		
				(0.04)	(0.05)	(0.04)		
Delayed (L-R):Trial				0.19***	0.12**	0.19***		
				(0.04)	(0.04)	(0.04)		
Condition (Colors)			−0.06			−0.06		
			(0.07)			(0.07)		
Condition:Trial						−0.01		
						(0.05)		
Immediate (L-R):Condition			0.67***			0.68***		
			(0.15)			(0.15)		
Delayed (L-R):Condition			−0.17			−0.18		
			(0.14)			(0.14)		
Immediate (L-R):Condition:Trial						0.04		
						(0.07)		
Delayed (L-R):Condition:Trial						−0.07		
						(0.06)		
Passive							0.07	
							(0.11)	
Immediate (L-R):Passive							0.02	
							(0.29)	
Delayed (L-R):Passive							0.45	
							(0.23)	
Constant	0.02	−0.04	0.02	0.02	−0.03	0.02	0.01	0.04
	(0.05)	(0.05)	(0.05)	(0.05)	(0.05)	(0.05)	(0.07)	(0.08)
Observations	5,358	5,219	10,577	5,358	5,219	10,577	4,769	1,710
Log Likelihood	-2,643.61	-2,195.38	-4,842.52	-2,606.39	-2,167.94	-4,777.88	-1,967.13	-682.78
Akaike Inf. Crit.	5,305.23	4,408.77	9,709.05	5,236.78	4,359.87	9,591.75	3,958.26	1,383.56
Bayesian Inf. Crit.	5,364.50	4,467.81	9,796.24	5,315.82	4,438.60	9,722.55	4,035.89	1,432.56

Note: *p<0.05; **p<0.01; ***p<0.001

Table B. Type of reward effect on choice. Logistic regression of choice of left stimulus on difference in mean experienced immediate and delayed reward between left and right stimuli and interactions of these effects with trial number. (1,4) Patterns Task. (2,5) Colors Task. (3,6) Patterns and Colors Tasks. (7) Colors Task Passive and Active Learning Conditions. (8) Colors Task Passive Condition. Continuous variables are z-scored. Regressions include random intercepts and random slopes for difference in mean experienced immediate and delayed rewards between left and right stimuli at the subject level.

	log RT (s)		
	(patterns)	(colors)	(both)
	(1)	(2)	(3)
|VD Total| (L-R)	-0.04^{***}	-0.05^{***}	-0.04^{***}
	(0.01)	(0.01)	(0.01)
OV Total (L+R)	-0.05^{***}	-0.06^{***}	-0.05^{***}
	(0.01)	(0.01)	(0.01)
Condition (Colors)			0.66^{***}
			(0.03)
|VD Total| (L-R):Condition			-0.01
			(0.01)
OV Total (L+R):Condition			-0.01
			(0.01)
Constant	0.0004	0.66^{***}	0.0004
	(0.02)	(0.02)	(0.02)
Observations	5,358	5,219	10,577
Log Likelihood	-1,369.62	-2,018.76	-3,438.33
Akaike Inf. Crit.	2,759.24	4,057.53	6,902.66
Bayesian Inf. Crit.	2,825.10	4,123.13	6,997.13
Note:	*p<0.05; **p<0.01; ***p<0.001		

Table C. Value difference (VD) and overall value effects (OV) on response time. Linear regression of log RT on the absolute difference in mean experienced reward and mean experienced total reward between left and right stimuli. (1) Patterns Task. (2) Colors Task. (3) Patterns and Colors Tasks. Continuous variables are z-scored. Regressions include random intercepts and random slopes for the absolute difference in mean experiened reward and mean experienced total reward between left and right stimuli at the subject level.

	log RT (s)		
	(patterns)	(colors)	(both)
	(1)	(2)	(3)
VD Immediate (L-R)	-0.02^{***}	-0.04^{***}	-0.02^{***}
	(0.01)	(0.01)	(0.01)
VD Delayed (L-R)	-0.01	-0.002	-0.01
	(0.01)	(0.01)	(0.01)
OV Immediate (L+R)	-0.04^{***}	-0.07^{***}	-0.04^{***}
	(0.01)	(0.01)	(0.01)
OV Delayed (L+R)	-0.03^{***}	-0.03^{***}	-0.03^{***}
	(0.01)	(0.01)	(0.01)
Condition (Colors)			0.66^{***}
			(0.03)
VD Immediate (L-R):Condition			-0.02^{**}
			(0.01)
VD Delayed (L-R):Condition			0.01
			(0.01)
OV Immediate (L+R):Condition			-0.03^{**}
			(0.01)
OV Delayed (L+R):Condition			0.002
			(0.01)
Constant	0.0004	0.66^{***}	0.0004
	(0.02)	(0.02)	(0.02)
Observations	5,358	5,219	10,577
Log Likelihood	-1,379.31	-1,988.68	-3,418.83
Akaike Inf. Crit.	2,800.63	4,019.36	6,889.66
Bayesian Inf. Crit.	2,938.94	4,157.12	7,078.58
Note:			*p<0.05; **p<0.01; ***p<0.001

Table D. Value difference (VD) and overall value (OV) effects by type of reward on response time. Linear regression of log RT on the absolute difference in mean experienced reward and mean experienced total reward between left and right stimuli for both immediate and delayed rewards. (1) Patterns Task. (2) Colors Task. (3) Patterns and Colors Tasks. Continuous variables are z-scored. Regressions include random intercepts and random slopes for the absolute difference in mean experienced reward and mean experienced total reward between left and right stimuli for both immediate and delayed rewards at the subject level.

	log RT (s)		
	(patterns)	(colors)	(both)
	(1)	(2)	(3)
Condition (Colors)			0.68***
			(0.03)
Reward Total (L-R)	−0.002	−0.01***	−0.002
	(0.002)	(0.002)	(0.002)
Reward Total Squared (L-R)	−0.001***	−0.002***	−0.001***
	(0.0002)	(0.0002)	(0.0002)
Reward Total (L-R):Condition			−0.01**
			(0.003)
Reward Total Squared (L-R):Condition			−0.001*
			(0.0003)
Constant	0.04	0.72***	0.04
	(0.03)	(0.02)	(0.02)
Observations	3,211	3,131	6,342
Log Likelihood	-897.61	-1,291.13	-2,219.42
Akaike Inf. Crit.	1,809.22	2,596.26	4,458.84
Bayesian Inf. Crit.	1,851.74	2,638.61	4,526.39
Note:			*p<0.05; **p<0.01; ***p<0.001

Table E. Value difference effects between descending and ascending options on response time. Linear regression of log RT on the absolute difference in underlying reward between descending and ascending stimuli and the square of the absolute difference in underlying reward between descending and ascending stimuli. (1) Patterns Task. (2) Colors Task. (3) Patterns and Colors Tasks. (1,2) Regressions include random intercepts and random slopes for the absolute difference in underlying reward between descending and ascending stimuli at the subject level.

	Dwell Proportion (I-D)				P(First Fix to I)			
	(patterns)	(patterns)	(inlab)	(inlab)	(patterns)	(patterns)	(inlab)	(inlab)
	(1)	(2)	(3)	(4)	(5)	(6)	(7)	(8)
Immediate Points	-0.01	-0.0002	0.07^{***}	0.08^{***}	0.01	-0.02	0.06	0.12
	(0.01)	(0.02)	(0.01)	(0.01)	(0.03)	(0.07)	(0.07)	(0.11)
Delayed Points	-0.02^{**}	0.003	-0.09^{***}	-0.09^{***}	-0.04	0.15^{*}	0.01	-0.10
	(0.01)	(0.02)	(0.01)	(0.01)	(0.04)	(0.08)	(0.07)	(0.11)
Immediate Stimulus Asc	-0.01	-0.001	0.20^{***}	0.22^{***}	0.06	-0.08	-0.12	0.20
	(0.01)	(0.03)	(0.03)	(0.04)	(0.07)	(0.14)	(0.20)	(0.31)
Delayed Stimulus Desc	0.01	0.02	-0.05^{***}	-0.03	0.07	-0.27	-0.22^{**}	-0.23
	(0.01)	(0.03)	(0.01)	(0.02)	(0.07)	(0.15)	(0.08)	(0.12)
Position (Immediate Bottom)				-0.05				-1.91^{***}
				(0.05)				(0.35)
Immediate Points:Position				-0.02				-0.10
				(0.02)				(0.14)
Delayed Points:Position				0.005				0.19
				(0.02)				(0.15)
Immediate Stimulus Asc:Position				-0.04				-0.56
				(0.06)				(0.40)
Delayed Stimulus Asc:Position				-0.03				-0.001
				(0.03)				(0.15)
Constant	0.05	-0.02	0.002	0.02	0.09	0.31	0.98^{***}	1.85^{***}
	(0.03)	(0.05)	(0.03)	(0.04)	(0.11)	(0.23)	(0.24)	(0.25)
Observations	7,795	1,863	4,763	4,763	7,720	1,845	4,717	4,717
Log Likelihood	-4,442.95	-1,290.41	-2,687.06	-2,697.90	-4,914.60	-1,170.21	-2,372.51	-2,352.47
Akaike Inf. Crit.	8,899.90	2,594.82	5,388.12	5,419.80	9,841.19	2,352.42	4,757.02	4,726.94
Bayesian Inf. Crit.	8,948.63	2,633.53	5,433.40	5,497.43	9,882.90	2,385.54	4,795.77	4,797.99

Note: *p<0.05; **p<0.01; ***p<0.001

Table F. Attention and reward feedback. (1,2,3,4) Linear regression of difference in proportion of dwell time within a trial between immediate and delayed reward as a function of the size and type (descending vs. ascending) of reward. (1) Feedback Position Condition: Immediate Reward: Top - Delayed Reward: Bottom. (2) Feedback Position Condition: Immediate Reward: Bottom - Delayed Reward: Top. (3,4) In-lab Eye-tracking Study. (5,6,7,8) Logistic regression of first fixation location (immediate vs. delayed) as a function of the size and type (descending vs. ascending) of reward. (5) Feedback Position Condition: Immediate Reward: Top - Delayed Reward: Bottom. (6) Feedback Position Condition: Immediate Reward: Bottom - Delayed Reward: Top. (7,8) In-lab Eye-tracking Study. Continuous variables are z-scores. Regressions include random intercept at the subject level.

	Dwell Proportion (I-D)			P(First Fix to I)		
	(patterns)	(patterns)	(inlab)	(patterns)	(patterns)	(inlab)
	(1)	(2)	(3)	(4)	(5)	(6)
|Prediction Error Immediate|	-0.001	0.01^{*}	-0.01^{**}	0.02	0.04	-0.01
	(0.002)	(0.01)	(0.004)	(0.01)	(0.03)	(0.02)
|Prediction Error Delayed|	-0.003	0.01	-0.005	0.004	-0.01	-0.01
	(0.002)	(0.005)	(0.002)	(0.01)	(0.02)	(0.02)
Predicted Value Immediate	-0.001	-0.01	0.01^{*}	-0.01	-0.02	0.05^{**}
	(0.002)	(0.005)	(0.003)	(0.01)	(0.02)	(0.02)
Predicted Value Delayed	-0.01^{**}	-0.002	0.02^{***}	-0.01	0.05	0.02
	(0.002)	(0.01)	(0.01)	(0.01)	(0.03)	(0.04)
Constant	0.09^{**}	-0.03	0.03	0.21	0.02	0.67^{*}
	(0.03)	(0.07)	(0.04)	(0.14)	(0.31)	(0.27)
Observations	7,634	1,845	4,716	7,634	1,845	4,716
Log Likelihood	-4,320.62	-1,274.15	-2,582.94	-4,846.06	-1,169.72	-2,372.63
Akaike Inf. Crit.	8,657.24	2,564.30	5,179.88	9,706.12	2,353.44	4,757.26
Bayesian Inf. Crit.	8,712.76	2,608.46	5,225.09	9,754.70	2,392.08	4,796.01
Note:						*p<0.05; **p<0.01; ***p<0.001

Table G. Attention and model predictions. (1,2,3) Linear regression of difference in proportion of dwell time within a trial between immediate and delayed reward as a function of the absolute prediction error and the predicted values for the immediate and delayed rewards derived from the differential learning model. (1) Feedback Position Condition: Immediate Reward: Top - Delayed Reward: Bottom. (2) Feedback Position Condition: Immediate Reward: Bottom - Delayed Reward: Top. (3) In-lab Eye-tracking Study. (4,5,6) Logistic regression of first fixation location (immediate vs. delayed) as a function of the absolute prediction error and the predicted values for the immediate and delayed rewards derived from the differential learning model. Continuous variables are z-scores. Regressions include random intercept at the subject and trial level. (4) Feedback Position Condition: Immediate Reward: Top - Delayed Reward: Bottom. (5) Feedback Position Condition: Immediate Reward: Bottom - Delayed Reward: Top. (6) In-lab Eye-tracking Study.

	Choice (Left)	
	(patterns)	(patterns)
	(1)	(2)
Immediate (L-R)	1.25***	1.31***
	(0.09)	(0.16)
Delayed (L-R)	0.72***	0.48***
	(0.08)	(0.11)
Dwell Proportion (L-R)	0.43***	0.49***
	(0.07)	(0.11)
Constant	−0.08	0.12
	(0.06)	(0.10)
Observations	7,698	1,831
Log Likelihood	-4,276.45	-1,017.00
Akaike Inf. Crit.	8,580.91	2,054.00
Bayesian Inf. Crit.	8,678.19	2,109.13
Note:	*p<0.05; **p<0.01; ***p<0.001	

Table H. Dwell proportion difference on choice. (1,2) Logistic regression of choice of left stimulus on difference in mean experienced reward between left and right stimulus for immediate and delayed rewards and the proportion of dwell time between left and right options within a trial. Continuous variables are z-scored. Regressions include random intercept and slopes for difference in mean experienced reward between left and right stimulus for immediate and delayed rewards and the proportion of dwell time between left and right options within a trial at the subject level. (1) Feedback Position Condition: Immediate Reward: Top - Delayed Reward: Bottom. (2) Feedback Position Condition: Immediate Reward: Bottom - Delayed Reward: Top.

	Mean Error		Incongruent Set Error		Congruent Set Error	
	(patterns)	(inlab)	(patterns)	(inlab)	(patterns)	(inlab)
	(1)	(2)	(3)	(4)	(5)	(6)
Survey 1 Rankings Error Immediate	0.01	0.02	−0.04***	−0.08***	0.04**	0.05***
	(0.01)	(0.01)	(0.01)	(0.02)	(0.01)	(0.01)
Survey 1 Rankings Error Delayed	0.03***	0.03***	0.05***	0.02	0.01	0.02
	(0.01)	(0.01)	(0.01)	(0.02)	(0.01)	(0.01)
Survey 2 Reward Comparison Error	0.02	0.004	−0.02	−0.05	0.05*	0.04
	(0.01)	(0.02)	(0.02)	(0.03)	(0.02)	(0.02)
Survey 3 Points Error	0.01		0.02		0.02	
	(0.01)		(0.01)		(0.01)	
Survey 3 Points Error Immediate		0.04		0.18**		0.04
		(0.03)		(0.06)		(0.04)
Survey 3 Points Error Delayed		0.05		0.12*		−0.001
		(0.03)		(0.05)		(0.03)
Constant	−0.01	−0.24**	0.26*	0.03	−0.13	−0.25*
	(0.07)	(0.09)	(0.10)	(0.16)	(0.11)	(0.11)
Observations	73	46	73	46	73	46
R^2	0.23	0.51	0.52	0.66	0.28	0.53
Adjusted R^2	0.18	0.45	0.49	0.62	0.24	0.47

Note: *p<0.05; **p<0.01; ***p<0.001

Table I. Choice and memory errors. (1,2) Linear regression of mean errors at the subject level on the memory error for each survey. (3,4) Linear regression of mean errors for incongruent choice sets (worse option descending, better option ascending) at the subject level on the memory error for each survey. (5,6) Linear regression of mean errors for congruent choice sets (worse option ascending, better option descending). (1,3,5) Study 2: online patterns task. (2,4,6) Study 4: in-lab patterns task. In Survey 1, subjects ranked the stimuli in terms of total reward. Using the same ranking responses, we separately computed Kendall tau distance relative to the true immediate-reward ordering and the true delayed-reward ordering. In Survey 2, subjects indicated whether each stimulus was ascending, descending, or flat. In Survey 3, subjects estimated the average total reward for each stimulus (separately for immediate and delayed rewards in Study 4).

	Bias Choice (patterns)	Bias Choice (inlab)	Bias Learning (patterns)	Bias Learning (inlab)
	(1)	(2)	(3)	(4)
Survey 1 Rankings Error Immediate	-0.24^{***}	-0.25^{*}	-0.04^{**}	-0.02
	(0.05)	(0.10)	(0.01)	(0.02)
Survey 1 Rankings Error Delayed	0.10^{*}	-0.25^{**}	0.005	-0.01
	(0.05)	(0.09)	(0.01)	(0.02)
Survey 2 Reward Comparison Error	-0.27^{**}	-0.26	-0.03	-0.04
	(0.09)	(0.15)	(0.02)	(0.02)
Survey 3 Points Error	0.03		-0.001	
	(0.06)		(0.01)	
Survey 3 Points Error Immediate		-0.37		0.03
		(0.31)		(0.05)
Survey 3 Points Error Delayed		0.04		0.02
		(0.24)		(0.04)
Constant	1.32^{**}	5.26^{***}	0.23	0.23
	(0.49)	(0.82)	(0.12)	(0.14)
Observations	73	46	73	46
R^2	0.50	0.40	0.19	0.18
Adjusted R^2	0.48	0.32	0.14	0.08
Note:		*p<0.05; **p<0.01; ***p<0.001		

Table J. Bias and memory errors. (1,2) Linear regression of behavioral bias at the subject level on the memory error for each survey. The behavioral bias is the difference in coefficients for the mean experienced immediate and delayed rewards from the mixed-effects logistic regression on choice. (3,4) Linear regression of difference in mean posterior of the learning rates between immediate and delayed rewards on the memory error for each survey. (1,3) Study 2: online patterns task. (2,4) Study 4: in-lab patterns task. In Survey 1, subjects ranked the stimuli in terms of total reward. Using the same ranking responses, we separately computed Kendall tau distance relative to the true immediate-reward ordering and the true delayed-reward ordering. In Survey 2, subjects indicated whether each stimulus was ascending, descending, or flat. In Survey 3, subjects estimated the average total reward for each stimulus (separately for immediate and delayed rewards in Study 4.

	Bias Choice					Bias Learning				
	(colors)	(colors)	(colors)	(colors)	(inlab)	(colors)	(colors)	(colors)	(colors)	(inlab)
	(1)	(2)	(3)	(4)	(5)	(6)	(7)	(8)	(9)	(10)
Accuracy 2-back	−2.85					-0.55^{*}				
	(1.57)					(0.23)				
D' 2-back			-0.31^{*}					-0.05^{*}		
			(0.14)					(0.02)		
Accuracy 3-back		−0.89					−0.07			
		(1.48)					(0.25)			
D' 3-back				−0.12					−0.02	
				(0.21)					(0.03)	
Accuracy visual					0.65					0.32
					(1.28)					(0.18)
Constant	3.87^{**}	1.70	2.14^{***}	1.19^{***}	1.35	0.64^{**}	0.17	0.29^{***}	0.15^{**}	−0.08
	(1.46)	(1.12)	(0.43)	(0.32)	(0.79)	(0.22)	(0.19)	(0.06)	(0.05)	(0.11)
Observations	86	51	86	51	46	86	51	86	51	46
R^2	0.04	0.01	0.06	0.01	0.01	0.06	0.002	0.07	0.01	0.07
Adjusted R^2	0.03	-0.01	0.04	-0.01	-0.02	0.05	-0.02	0.06	-0.01	0.05
Note:	*p<0.05; **p<0.01; ***p<0.001									

Table K. Bias and n-back working memory task performance. Linear regression of bias at the subject level on performance on the 2-back task, 3-back task and the change localization task. (1,2,5) Behavioral bias on accuracy. (3,4) Behavioral bias on d prime. (6,7,10) Difference in mean posterior of the learning rates between immediate and delayed rewards on accuracy. (8,9) Difference in mean posterior of the learning rates between immediate and delayed rewards on d prime. (1,2,6,7) Accuracy is measured as the correct responses (both correctly identified targets as well as correctly identified non-targets) out of total number of stimuli. (5,10) Accuracy is measured as the percent correct responses in the change localization task. (3,4,8,9) D prime is the sensitivity measure calculated as the difference in z-scored hit rates and z-scored false alarm rates. Hit rate refers to the proportion of correctly identified target stimuli (i.e., stimuli that match the one presented n items back). It is calculated as the number of correct responses divided by the total number of target stimuli. False alarm rate refers to the proportion of non-target stimuli that are incorrectly identified as targets. It is calculated as the number of false alarms divided by the total number of non-target stimuli. These measures where corrected to account for cases in which the hit rate equals 1 or false alarm rate equals 0.

	Today-1 Month (colors)	Today-1 Month (colors)	Today-6 Months (colors)	Today-6 Months (colors)	1 Month-6 Months (colors)	1 Month-6 Months (colors)
	(1)	(2)	(3)	(4)	(5)	(6)
Bias Choice	2.04^{*}		1.86		1.53	
	(0.95)		(1.10)		(1.04)	
Bias Learning		11.04		8.03		3.63
		(6.41)		(7.45)		(7.04)
Constant	109.69^{***}	110.77^{***}	116.74^{***}	117.99^{***}	115.81^{***}	117.22^{***}
	(1.59)	(1.37)	(1.85)	(1.60)	(1.75)	(1.51)
Observations	87	87	87	87	87	87
R^2	0.05	0.03	0.03	0.01	0.02	0.003
Adjusted R^2	0.04	0.02	0.02	0.002	0.01	-0.01
Residual Std. Error (df = 85)	10.15	10.24	11.79	11.91	11.13	11.25
F Statistic (df = 1; 85)	4.62^{*}	2.97	2.84	1.16	2.15	0.27

Note: $^{*}p<0.05$; $^{**}p<0.01$; $^{***}p<0.001$

Table L. Bias and intertemporal preferences. (1, 3, 5) Linear regression of intertemporal choice indifference point on behavioral bias at the subject level. The behavioral bias is the difference in coefficients of mean experienced immediate and delayed rewards from the mixed effects logistic regression on choice. (2, 4, 6) Linear regression of intertemporal choice indifference point on difference in mean posterior of the learning rates between immediate and delayed rewards.

	Today-1 Month		Today-6 Months		1 Month-6 Months	
	(inlab)	(inlab)	(inlab)	(inlab)	(inlab)	(inlab)
	(1)	(2)	(3)	(4)	(5)	(6)
Bias Choice	−2.00		−1.94		−1.48	
	(1.61)		(1.79)		(1.74)	
Bias Learning		−10.34		−11.13		−19.45
		(11.29)		(12.50)		(11.88)
Constant	116.22***	113.87***	122.43***	120.26***	120.44***	120.00***
	(3.22)	(2.02)	(3.57)	(2.23)	(3.49)	(2.12)
Observations	46	46	46	46	46	46
R^2	0.03	0.02	0.03	0.02	0.02	0.06
Adjusted R^2	0.01	-0.004	0.004	-0.005	-0.01	0.04
Residual Std. Error (df = 44)	10.71	10.80	11.90	11.95	11.61	11.37
F Statistic (df = 1; 44)	1.55	0.84	1.18	0.79	0.72	2.68
Note:				*p<0.05; **p<0.01; ***p<0.001		

Table M. Bias and intertemporal preferences in-lab eye-tracking study. (1, 3, 5) Linear regression of intertemporal choice indifference point on behavioral bias at the subject level. The behavioral bias is the difference in coefficients of mean experienced immediate and delayed rewards from the mixed effects logistic regression on choice. (2, 4, 6) Linear regression of intertemporal choice indifference point on difference in mean posterior of the learning rates between immediate and delayed rewards.

	Choice (Left)					
	(combined)	(separate)	(combined)	(combined)	(separate)	(separate)
	(1)	(2)	(3)	(4)	(5)	(6)
Right Desc	-0.45^{***}	-0.39^{***}	-0.67^{***}	1.51^{***}	-0.57^{*}	1.74^{***}
	(0.07)	(0.10)	(0.17)	(0.26)	(0.24)	(0.34)
Left Desc	0.45^{***}	0.29^{**}	0.56^{***}	-1.55^{***}	0.37	-1.80^{***}
	(0.07)	(0.10)	(0.17)	(0.26)	(0.22)	(0.34)
Total (L-R)	1.60^{***}	1.58^{***}		2.22^{***}		2.34^{***}
	(0.12)	(0.17)		(0.22)		(0.30)
Position (Immediate Bottom)		−0.01			−0.17	−0.21
		(0.15)			(0.18)	(0.24)
Right Desc:Position		−0.12			−0.21	−0.52
		(0.15)			(0.35)	(0.50)
Left Desc:Position		0.32^{*}			0.39	0.57
		(0.15)			(0.33)	(0.50)
Total (L-R):Position		0.06				−0.28
		(0.23)				(0.43)
Constant	−0.01	−0.001	0.12	0.09	0.20	0.18
	(0.08)	(0.10)	(0.09)	(0.12)	(0.12)	(0.17)
Observations	4,740	4,740	2,799	2,799	2,799	2,799
Log Likelihood	-2,433.78	-2,430.14	-1,787.52	-1,496.03	-1,786.22	-1,494.68
Akaike Inf. Crit.	4,881.55	4,882.29	3,593.03	3,020.06	3,596.45	3,025.36
Bayesian Inf. Crit.	4,926.80	4,953.39	3,646.46	3,103.18	3,667.69	3,132.23
Note:						*p<0.05; **p<0.01; ***p<0.001

Table N. Type of option effect on choice by feedback position. Logistic regression of choice of left stimulus on whether the right and left stimuli are descending or ascending controlling for difference in mean experienced total reward between left and right stimuli. (1,3,4) Combined position conditions. (2,5,6) Separate position conditions. (1,2) Online Patterns Study. (3,4,5,6) In-lab Eye-tracking Study. Continuous variables are z-scored. Regressions include random intercepts and random slopes for whether the right and left stimuli are descending or ascending and for difference in mean experienced total reward at the subject level.

	Error			
	(combined)	(separate)	(combined)	(separate)
	(1)	(2)	(3)	(4)
Set Type Congruent	−0.17***	−0.14**	−0.25***	−0.19**
	(0.03)	(0.05)	(0.04)	(0.06)
Position (Immediate Bottom)		0.01		0.10
		(0.05)		(0.06)
Set Type Congruent:Position		−0.07		−0.13
		(0.06)		(0.09)
Constant	0.40***	0.39***	0.43***	0.39***
	(0.02)	(0.03)	(0.03)	(0.04)
Observations	160	160	94	94
R^2	0.15	0.16	0.26	0.28
Adjusted R^2	0.15	0.14	0.25	0.26
Residual Std. Error	0.20 (df = 158)	0.20 (df = 156)	0.21 (df = 92)	0.21 (df = 90)
F Statistic	28.31*** (df = 1; 158)	9.88*** (df = 3; 156)	31.77*** (df = 1; 92)	11.63*** (df = 3; 90)
Note:				*p<0.05; **p<0.01; ***p<0.001

Table O. Type of choice set effect on error rate by feedback position. Linear regression of type of choice set (incongruent or congruent) on mean error rate. (1,3) Combined position conditions. (2,4) Separate position conditions. (1,2) Online Patterns Study. (3,4) In-lab Eye-tracking Task.

	Choice (Left)							
	(combined)	(separate)	(combined)	(separate)	(combined)	(separate)	(combined)	(separate)
	(1)	(2)	(3)	(4)	(5)	(6)	(7)	(8)
Immediate (L-R)	1.60***	1.48***	1.52***	1.53***	1.49***	1.42***	1.53***	1.47***
	(0.24)	(0.15)	(0.11)	(0.16)	(0.15)	(0.20)	(0.15)	(0.20)
Delayed (L-R)	0.72***	1.03***	1.03***	1.06***	−0.35***	−0.30*	−0.46***	−0.41**
	(0.16)	(0.15)	(0.11)	(0.15)	(0.10)	(0.14)	(0.11)	(0.14)
Trial			0.08*	0.08			−0.02	0.07
			(0.04)	(0.05)			(0.05)	(0.07)
Immediate (L-R):Trial			0.34***	0.40***			0.29***	0.30***
			(0.04)	(0.06)			(0.06)	(0.08)
Delayed (L-R):Trial			0.06	0.08			−0.25***	−0.23**
			(0.04)	(0.06)			(0.05)	(0.07)
Position (Immediate Bottom)		0.08		0.08		−0.22		−0.24
		(0.10)		(0.10)		(0.17)		(0.17)
Position:Trial				0.003				−0.19
				(0.08)				(0.10)
Immediate (L-R):Position		0.004		−0.02		0.15		0.14
		(0.22)		(0.22)		(0.29)		(0.30)
Delayed (L-R):Position		−0.04		−0.05		−0.11		−0.12
		(0.21)		(0.21)		(0.20)		(0.21)
Immediate (L-R):Position:Trial				−0.11				−0.002
				(0.09)				(0.12)
Delayed (L-R):Position:Trial				−0.03				−0.04
				(0.08)				(0.11)
Constant	0.19*	−0.03	0.02	−0.02	0.09	0.20	0.09	0.20
	(0.09)	(0.07)	(0.05)	(0.07)	(0.09)	(0.11)	(0.09)	(0.11)
Observations	1,077	4,740	4,740	4,740	2,799	2,799	2,799	2,799
Log Likelihood	-532.01	-2,370.70	-2,337.84	-2,336.77	-1,479.77	-1,478.60	-1,461.08	-1,458.07
Akaike Inf. Crit.	1,082.02	4,765.40	4,699.68	4,709.54	2,977.53	2,981.20	2,946.15	2,952.14
Bayesian Inf. Crit.	1,126.86	4,842.96	4,777.25	4,825.89	3,030.96	3,052.45	3,017.39	3,059.00

Note: *p<0.05; **p<0.01; ***p<0.001

Table P. Type of reward effect on choice by feedback position. Logistic regression of choice of left stimulus on difference in mean experienced immediate and delayed rewards between left and right stimuli and interactions of these effects with trial number. (1,3,5,7) Combined position conditions. (2,4,6,8) Separate position conditions. (1,2,3,4) Online Patterns Study. (5,6,7,8) In-lab Eye-tracking Study. Continuous variables are z-scored. Regressions include random intercepts and random slopes for difference in mean experienced immediate and delayed rewards between left and right stimuli at the subject level.

	log RT (s)			
	(combined)	(separate)	(combined)	(separate)
	(1)	(2)	(3)	(4)
VD Total (L-R)	-0.03^{***}	-0.03^{***}	-0.02	-0.01
	(0.01)	(0.01)	(0.01)	(0.01)
OV Total (L+R)	-0.04^{***}	-0.04^{***}	-0.03^{**}	-0.01
	(0.01)	(0.01)	(0.01)	(0.01)
Position (Immediate Bottom)		0.06		-0.01
		(0.05)		(0.07)
VD Total (L-R):Position		0.005		-0.02
		(0.01)		(0.02)
OV Total (L+R):Position		0.001		-0.04^{*}
		(0.01)		(0.02)
Constant	0.01	-0.02	0.14^{***}	0.14^{**}
	(0.03)	(0.04)	(0.04)	(0.05)
Observations	4,740	4,740	2,799	2,799
Log Likelihood	-1,521.34	-1,529.24	-741.15	-746.76
Akaike Inf. Crit.	3,062.68	3,084.49	1,502.30	1,519.52
Bayesian Inf. Crit.	3,127.32	3,168.52	1,561.67	1,596.70
Note:		*p<0.05; **p<0.01; ***p<0.001		

Table Q. Value difference (VD) and overall value effects (OV) on response time by feedback position. Linear regression of log RT on the absolute difference in mean experienced reward and mean experienced total reward between left and right stimuli. (1,3) Combined position conditions. (2,4) Separate position conditions. (1,2) Online Patters Study. (3,4) In-lab Eye-tracking Study. Continuous variables are z-scored. Regressions include random intercepts and random slopes for the absolute difference in mean experienced reward and mean experienced total reward between left and right stimuli at the subject level.

	log RT (s)			
	(combined)	(separate)	(combined)	(separate)
	(1)	(2)	(3)	(4)
VD Immediate (L-R)	-0.03^{***}	-0.03^{***}	-0.04^{***}	-0.01
	(0.01)	(0.01)	(0.01)	(0.01)
VD Delayed (L-R)	-0.02^{**}	-0.02^{*}	0.01^{*}	0.01
	(0.01)	(0.01)	(0.01)	(0.01)
OV Immediate (L+R)	-0.03^{***}	-0.03^{***}	-0.03^{***}	-0.01
	(0.005)	(0.01)	(0.01)	(0.01)
OV Delayed (L+R)	-0.03^{***}	-0.03^{***}	0.02^{**}	0.01
	(0.005)	(0.01)	(0.01)	(0.01)
Position (Immediate Bottom)		0.06		-0.01
		(0.05)		(0.07)
VD Immediate (L-R):Position		0.002		-0.05^{***}
		(0.01)		(0.01)
VD Delayed (L-R):Position		0.001		0.01
		(0.01)		(0.01)
OV Immediate (L+R):Position		-0.01		-0.04^{***}
		(0.01)		(0.01)
OV Delayed (L+R):Position		0.01		0.03^{*}
		(0.01)		(0.01)
Constant	0.01	-0.02	0.14^{***}	0.14^{**}
	(0.03)	(0.04)	(0.04)	(0.05)
Observations	4,740	4,740	2,799	2,799
Log Likelihood	-1,571.04	-1,586.22	-771.04	-769.58
Akaike Inf. Crit.	3,156.08	3,196.44	1,556.08	1,563.17
Bayesian Inf. Crit.	3,201.33	3,274.01	1,597.64	1,634.41
Note:	*p<0.05; **p<0.01; ***p<0.001			

Table R. Value difference (VD) and overall value (OV) effects by type of reward on response time by feedback position. Linear regression of log RT on the absolute difference in mean experienced reward and mean experienced total reward between left and right stimuli for both immediate and delayed rewards. (1,3) Combined position conditions. (2,4) Separate position conditions. (1,2) Online Patterns Study. (3,4) In-lab Eye-tracking Study. Continuous variables are z-scored. Regressions include random intercepts at the subject level.

	log RT (s)			
	(combined)	(separate)	(combined)	(separate)
	(1)	(2)	(3)	(4)
Position (Immediate Bottom)		0.06		0.001
		(0.06)		(0.07)
Total (L-R)	-0.002	-0.002	-0.01^{*}	-0.0004
	(0.002)	(0.003)	(0.003)	(0.004)
Total Squared (L-R)	-0.001^{***}	-0.001^{**}	-0.001^{***}	-0.002^{***}
	(0.0002)	(0.0003)	(0.0003)	(0.0004)
Total (L-R):Position		-0.001		-0.01^{**}
		(0.004)		(0.01)
Total Squared (L-R):Position		-0.0001		0.0002
		(0.0004)		(0.001)
Constant	0.03	-0.005	0.18^{***}	0.18^{***}
	(0.03)	(0.04)	(0.04)	(0.05)
Observations	2,851	2,851	1,679	1,679
Log Likelihood	-998.66	-1,011.42	-370.60	-379.73
Akaike Inf. Crit.	2,011.32	2,042.83	755.21	779.45
Bayesian Inf. Crit.	2,053.00	2,102.39	793.19	833.71
Note:				*p<0.05; **p<0.01; ***p<0.001

Table S. Value difference effects between descending and ascending options on response time by feedback position. Linear regression of log RT on the absolute difference in underlying reward between descending and ascending stimuli and the square of the absolute difference in underlying reward between descending and ascending stimuli. (1,3) Combined position conditions. (2,4) Separate position conditions. (1,2) Online Patterns Task. (3,4) In-lab Eye-tracking Task. Regressions include random intercepts and random slope for the absolute difference in underlying reward between descending and ascending stimuli at the subject level.

Supplementary Text B

Instructions: Colors Task

Welcome to today's experiment!

- The experiment consists of the following parts:
  1. A main task
  2. Some questions regarding this task, and your person
  3. A few shorter tasks
  4. Your payment – for which will call you to the front at the end

- You will receive more detailed instructions before each section.
- The experiment will take about 50 minutes in total.

- We begin by introducing the main task.
- Please read all instructions on the following pages carefully.
  - You can proceed by clicking "next".
  - Use "back" to return to previous pages.
  - If anything remains unclear after reading, simply raise your hand we will approach you at your cubicle to answer any questions.

next

First a short overview – details will follow:

- The main task consists of 105 rounds. In each round you can choose one of two colors.
- Each of these decisions generates points for you twice: Once immediately after the choice, once with one round delay.

- Your goal is to collect as many points as possible.

- How many points each choice yields depends on the chosen color: some colors are worth more points than others.
- You do not know in advance which of the colors yield many or few points – you can learn this while doing the task.

- The following page allows you to familiarize yourself with the task.
- It explains both the interface and how points are generated.

- **Important**:
  - The following page is for demonstration only!
  - The different options are represented by black, white, and shades of grey rather than colors.
  - All numbers are just examples.
  - The points you earn during the demonstration do not influence your payoff.

back next

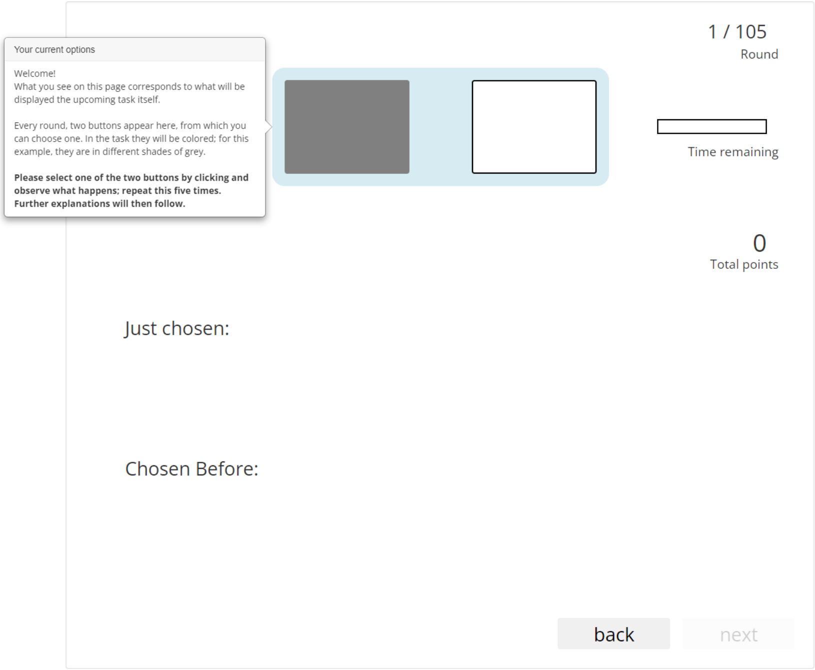
Your current options
Welcome!
What you see on this page corresponds to what will be displayed the upcoming task itself.
Every round, two buttons appear here, from which you can choose one. In the task they will be colored; for this example, they are in different shades of grey.
Please select one of the two buttons by clicking and observe what happens; repeat this five times. Further explanations will then follow.
1 / 105
Round
Time remaining
0
Total points
Just chosen:
Chosen Before:
back
next

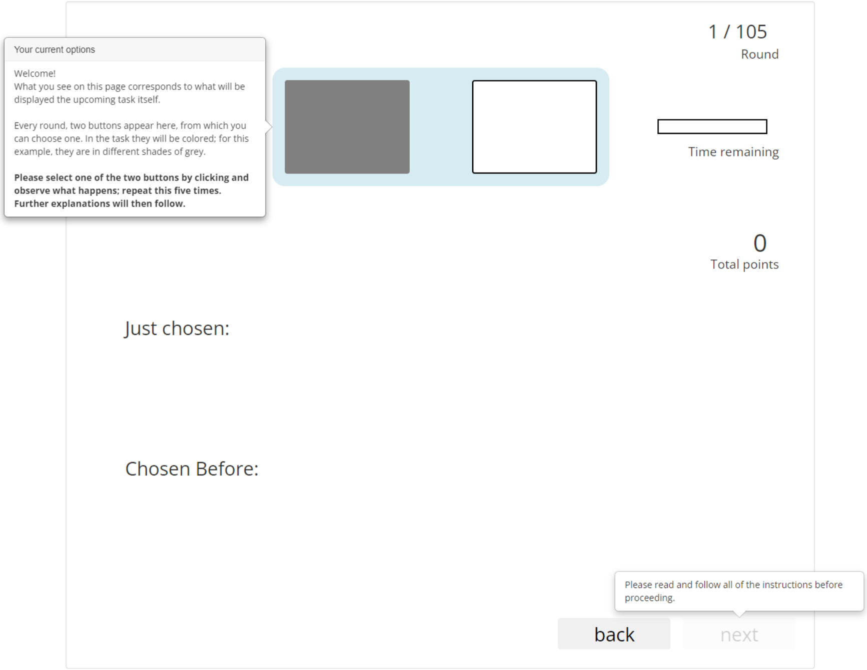
Your current options
Welcome!
What you see on this page corresponds to what will be displayed the upcoming task itself.
Every round, two buttons appear here, from which you can choose one. In the task they will be colored; for this example, they are in different shades of grey.
Please select one of the two buttons by clicking and observe what happens; repeat this five times. Further explanations will then follow.
1 / 105
Round
Time remaining
0
Total points
Just chosen:
Chosen Before:
Please read and follow all of the instructions before proceeding.
back
next

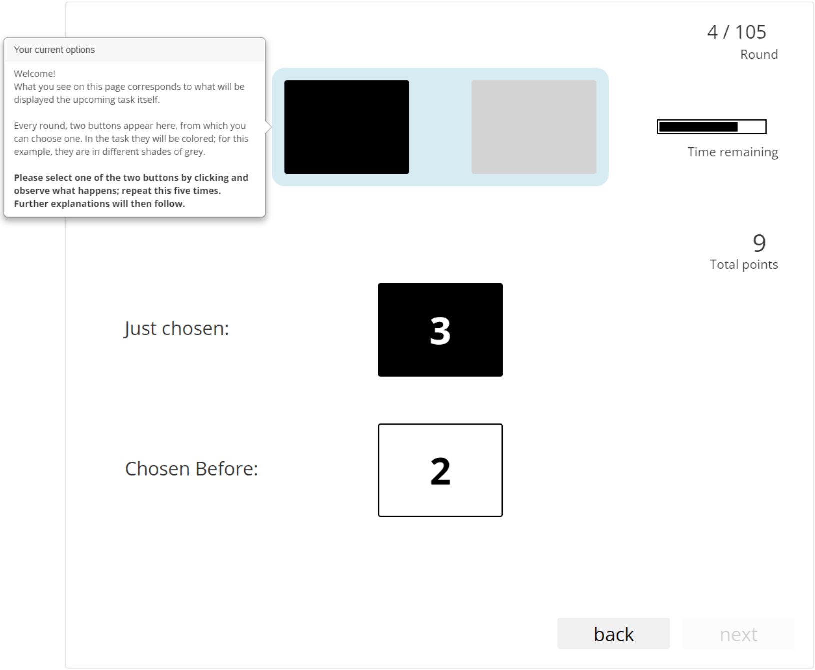

Your current options
Welcome!
What you see on this page corresponds to what will be displayed the upcoming task itself.
Every round, two buttons appear here, from which you can choose one. In the task they will be colored; for this example, they are in different shades of grey.
Please select one of the two buttons by clicking and observe what happens; repeat this five times. Further explanations will then follow.
4 / 105
Round
Time remaining
9
Total points
Just chosen:
3
Chosen Before:
2
back
next

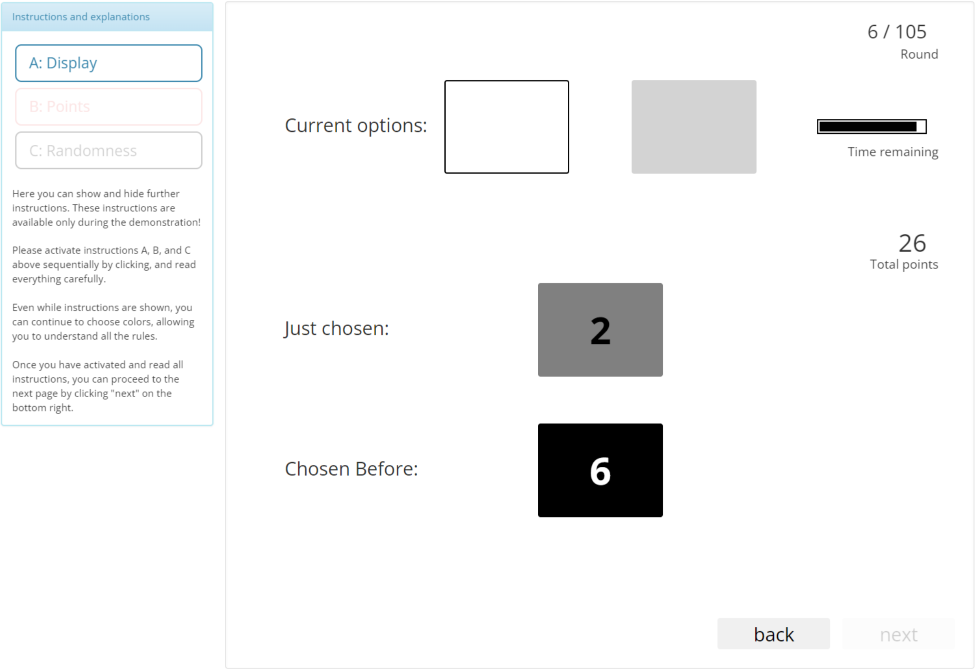

Instructions and explanations
A: Display
B: Points
C: Randomness
Here you can show and hide further instructions. These instructions are available only during the demonstration!
Please activate instructions A, B, and C above sequentially by clicking, and read everything carefully.
Even while instructions are shown, you can continue to choose colors, allowing you to understand all the rules.
Once you have activated and read all instructions, you can proceed to the next page by clicking "next" on the bottom right.
6 / 105
Round
Current options:
Time remaining
26
Total points
Just chosen:
2
Chosen Before:
6
back
next

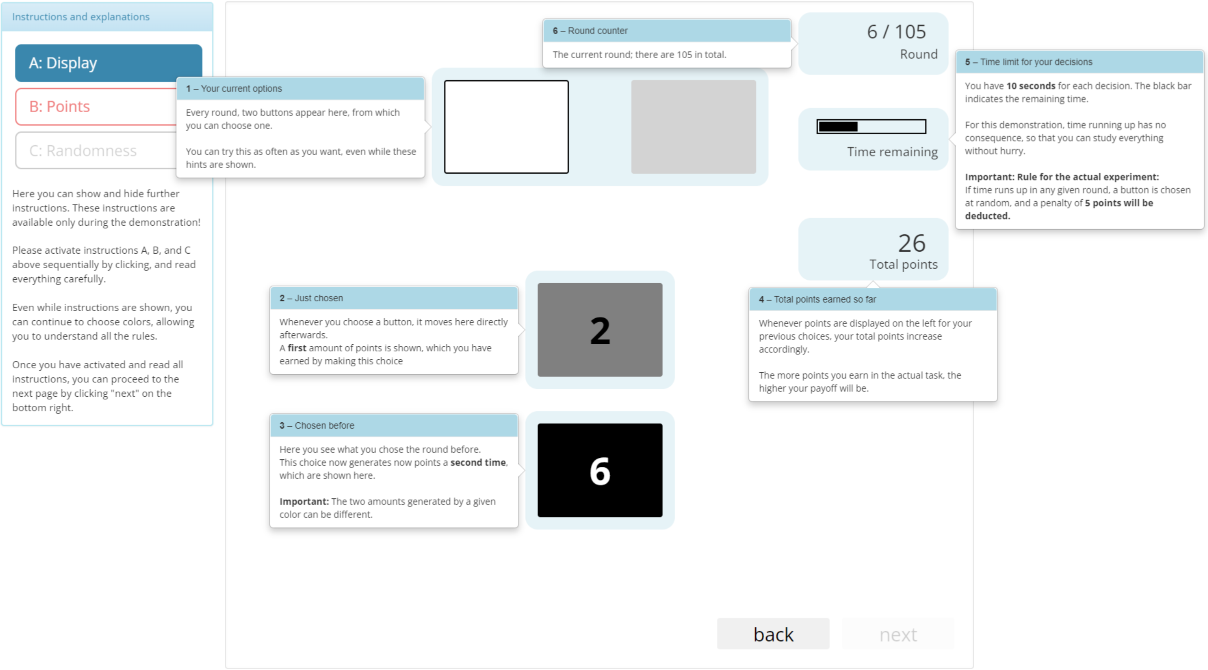
Instructions and explanations
A: Display
B: Points
C: Randomness
Here you can show and hide further instructions. These instructions are available only during the demonstration!
Please activate instructions A, B, and C above sequentially by clicking, and read everything carefully.
Even while instructions are shown, you can continue to choose colors, allowing you to understand all the rules.
Once you have activated and read all instructions, you can proceed to the next page by clicking "next" on the bottom right.
6 – Round counter
The current round; there are 105 in total.
6 / 105
Round
1 – Your current options
Every round, two buttons appear here, from which you can choose one.
You can try this as often as you want, even while these hints are shown.
5 – Time limit for your decisions
You have **10 seconds** for each decision. The black bar indicates the remaining time.
For this demonstration, time running up has no consequence, so that you can study everything without hurry.
Important: Rule for the actual experiment:
If time runs up in any given round, a button is chosen at random, and a penalty of **5 points will be deducted.**
Time remaining
26
Total points
2 – Just chosen
Whenever you choose a button, it moves here directly afterwards.
A **first** amount of points is shown, which you have earned by making this choice
2
4 – Total points earned so far
Whenever points are displayed on the left for your previous choices, your total points increase accordingly.
The more points you earn in the actual task, the higher your payoff will be.
3 – Chosen before
Here you see what you chose the round before.
This choice now generates now points a **second time**, which are shown here.
Important: The two amounts generated by a given color can be different.
6
back
next

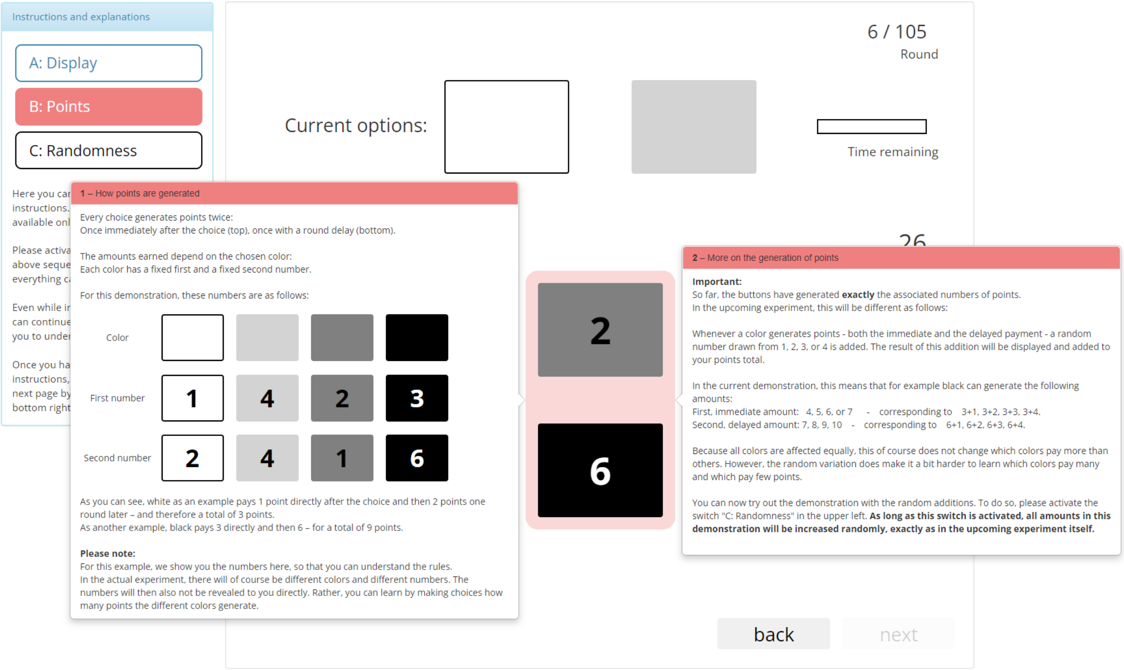
Instructions and explanations
A: Display
B: Points
C: Randomness
Here you car
instructions.
available onl
Please activa
above seque
everything c
Even while ir
can continue
you to under
Once you ha
instructions,
next page by
bottom right
6 / 105
Round
Current options:
Time remaining
1 – How points are generated
Every choice generates points twice:
Once immediately after the choice (top), once with a round delay (bottom).
The amounts earned depend on the chosen color:
Each color has a fixed first and a fixed second number.
For this demonstration, these numbers are as follows:
Color
First number
1
4
2
3
Second number
2
4
1
6
As you can see, white as an example pays 1 point directly after the choice and then 2 points one round later – and therefore a total of 3 points.
As another example, black pays 3 directly and then 6 – for a total of 9 points.
Please note:
For this example, we show you the numbers here, so that you can understand the rules.
In the actual experiment, there will of course be different colors and different numbers. The numbers will then also not be revealed to you directly. Rather, you can learn by making choices how many points the different colors generate.
2
6
2 – More on the generation of points
Important:
So far, the buttons have generated **exactly** the associated numbers of points.
In the upcoming experiment, this will be different as follows:
Whenever a color generates points - both the immediate and the delayed payment - a random number drawn from 1, 2, 3, or 4 is added. The result of this addition will be displayed and added to your points total.
In the current demonstration, this means that for example black can generate the following amounts:
First, immediate amount: 4, 5, 6, or 7 - corresponding to 3+1, 3+2, 3+3, 3+4.
Second, delayed amount: 7, 8, 9, 10 - corresponding to 6+1, 6+2, 6+3, 6+4.
Because all colors are affected equally, this of course does not change which colors pay more than others. However, the random variation does make it a bit harder to learn which colors pay many and which pay few points.
You can now try out the demonstration with the random additions. To do so, please activate the switch "C: Randomness" in the upper left. **As long as this switch is activated, all amounts in this demonstration will be increased randomly, exactly as in the upcoming experiment itself.**
back
next

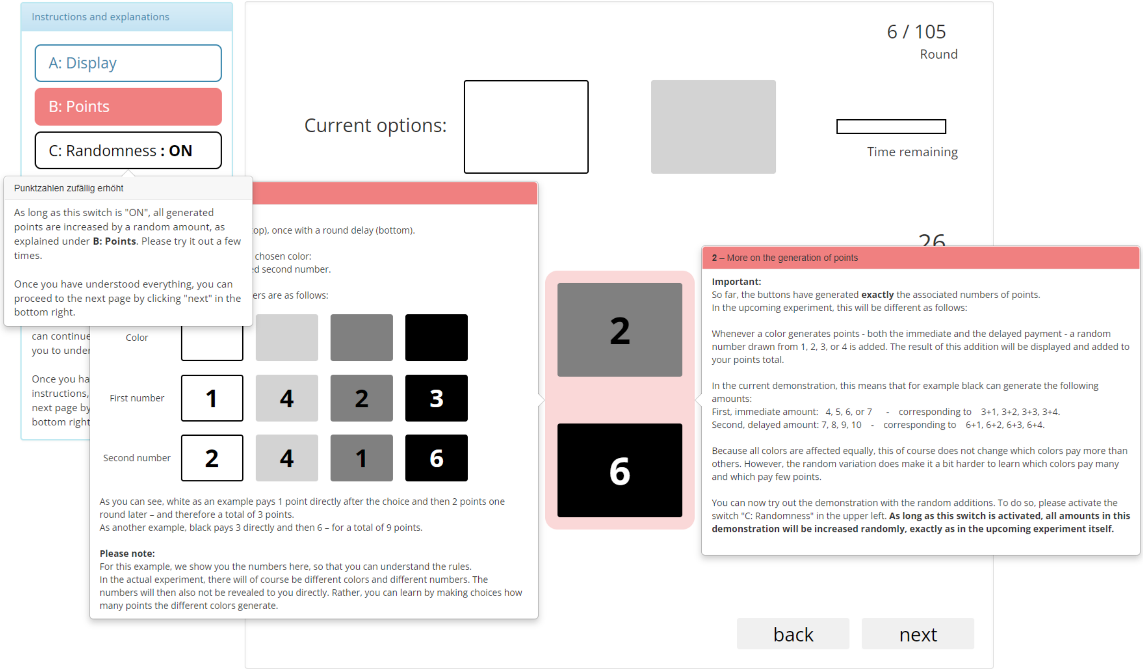
Instructions and explanations
A: Display
B: Points
C: Randomness : ON
Punktzahlen zufällig erhöht
As long as this switch is "ON", all generated points are increased by a random amount, as explained under B: Points. Please try it out a few times.
Once you have understood everything, you can proceed to the next page by clicking "next" in the bottom right.
6 / 105
Round
Current options:
Time remaining
op), once with a round delay (bottom).
chosen color:
d second number.
ers are as follows:
Color
First number
Second number
1 4 2 3
2 4 1 6
As you can see, white as an example pays 1 point directly after the choice and then 2 points one round later – and therefore a total of 3 points.
As another example, black pays 3 directly and then 6 – for a total of 9 points.
Please note:
For this example, we show you the numbers here, so that you can understand the rules.
In the actual experiment, there will of course be different colors and different numbers. The numbers will then also not be revealed to you directly. Rather, you can learn by making choices how many points the different colors generate.
2
6
2 – More on the generation of points
Important:
So far, the buttons have generated exactly the associated numbers of points.
In the upcoming experiment, this will be different as follows:
Whenever a color generates points - both the immediate and the delayed payment - a random number drawn from 1, 2, 3, or 4 is added. The result of this addition will be displayed and added to your points total.
In the current demonstration, this means that for example black can generate the following amounts:
First, immediate amount: 4, 5, 6, or 7 - corresponding to 3+1, 3+2, 3+3, 3+4.
Second, delayed amount: 7, 8, 9, 10 - corresponding to 6+1, 6+2, 6+3, 6+4.
Because all colors are affected equally, this of course does not change which colors pay more than others. However, the random variation does make it a bit harder to learn which colors pay many and which pay few points.
You can now try out the demonstration with the random additions. To do so, please activate the switch "C: Randomness" in the upper left. As long as this switch is activated, all amounts in this demonstration will be increased randomly, exactly as in the upcoming experiment itself.
back
next

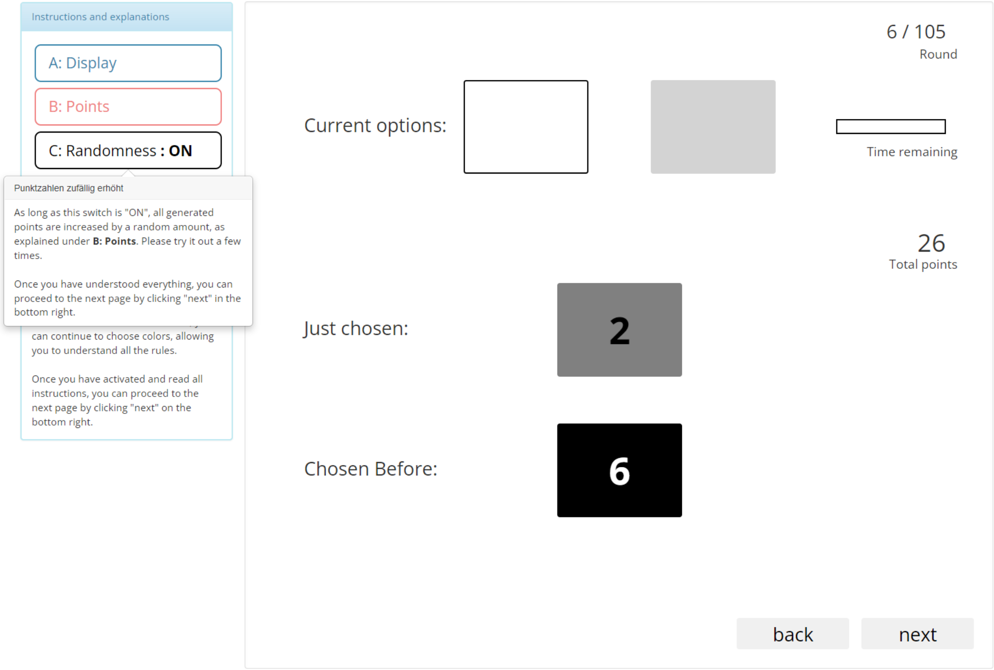
Instructions and explanations
A: Display
B: Points
C: Randomness : ON
Punktzahlen zufällig erhöht
As long as this switch is "ON", all generated points are increased by a random amount, as explained under B: Points. Please try it out a few times.
Once you have understood everything, you can proceed to the next page by clicking "next" in the bottom right.
can continue to choose colors, allowing you to understand all the rules.
Once you have activated and read all instructions, you can proceed to the next page by clicking "next" on the bottom right.
6 / 105
Round
Current options:
Time remaining
26
Total points
Just chosen:
2
Chosen Before:
6
back
next

To summarize:

- In each round, you choose one of two buttons.
- Each choice generates points *twice*.
- The amounts generated depend on the chosen color.
  First and second amounts generated by each color can be different.

- On top, 1, 2, 3 oder 4 points are always added at random – independently of the color.

- Your goal is to earn as many points as possible throughout the 105 rounds.
- To this end, you can learn bit by bit which colors yield more points than others.

- In each round, you have only 10 seconds to observe the latest numbers and choose the next color.
-
- A bar indicates the remaining time.
- If you do not choose in time, a color is picked at random for you.
  In addition, you incur a penalty of 5 points.

back next

- These are the six colors that are used in the experiment:

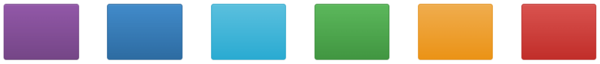

- If you have trouble differentiating any two of these colors:
  Please raise your hand **now** - an experimenter will come to your desk!

- Which two buttons are available in any given round does not depend on your past choices.

- It can happen that both buttons have the same color – this is on purpose.
  In this case it makes no difference which of the two you click.

back next

- Your payoff for this experiment depends considerably on how many points you earn in the upcoming task.
  - Thus, please make sure you understand all rules well before clicking on "start" below.
  - Before you start, you can go back in the instructions and also re-play the example, if you like.
  - If anything remains unclear, please let us know – we will come to your desk.

Your payment for this part of the experiment is calculated as follows:

- You receive **5 cents** for each point you earn **above 1800 points**.
- The first 1800 points are not paid.

- Example: Suppose you earn 1960 points – which is 160 above 1800.
  Thus, you would receive 160×0.05€ = 8.00€ for the main task.

back START

Instructions and Quiz: Patterns Task

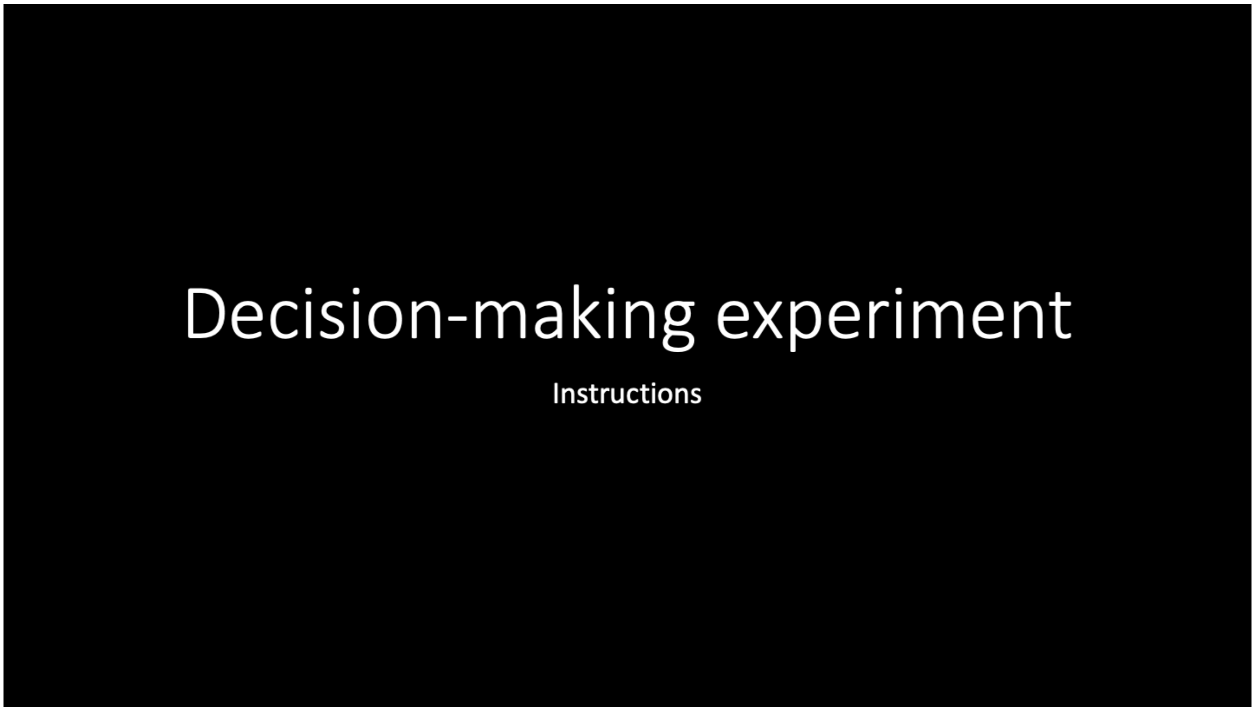

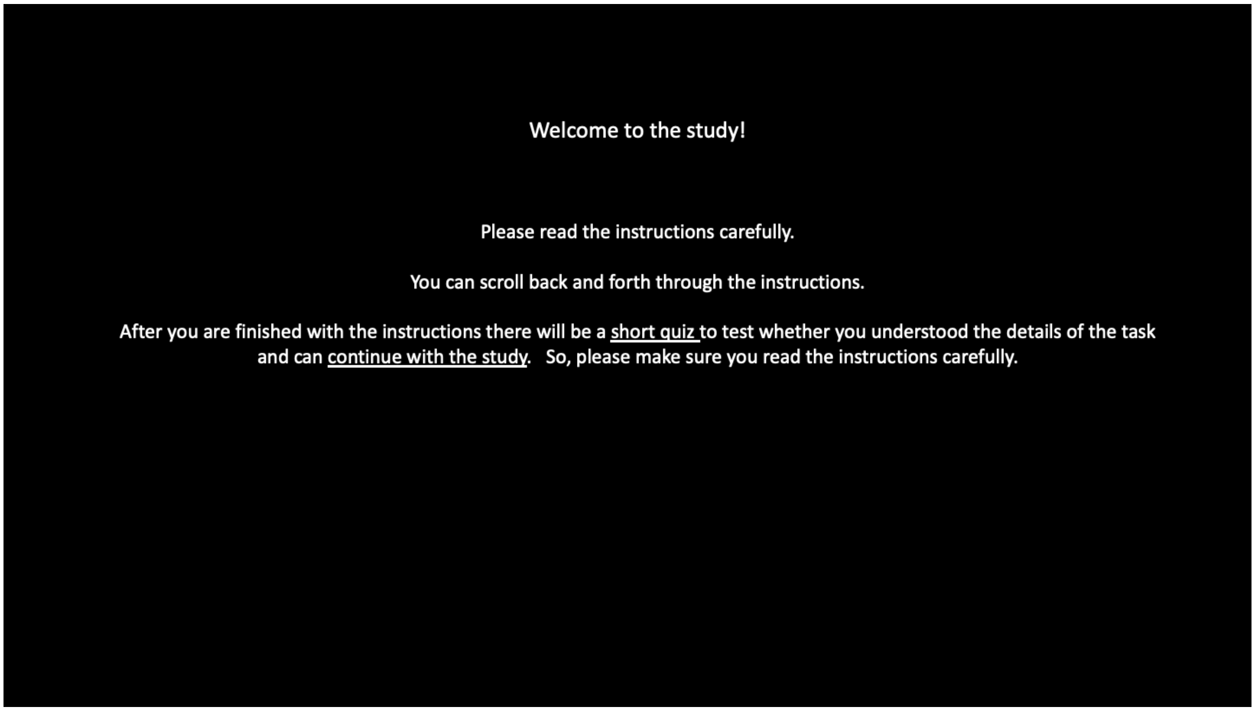

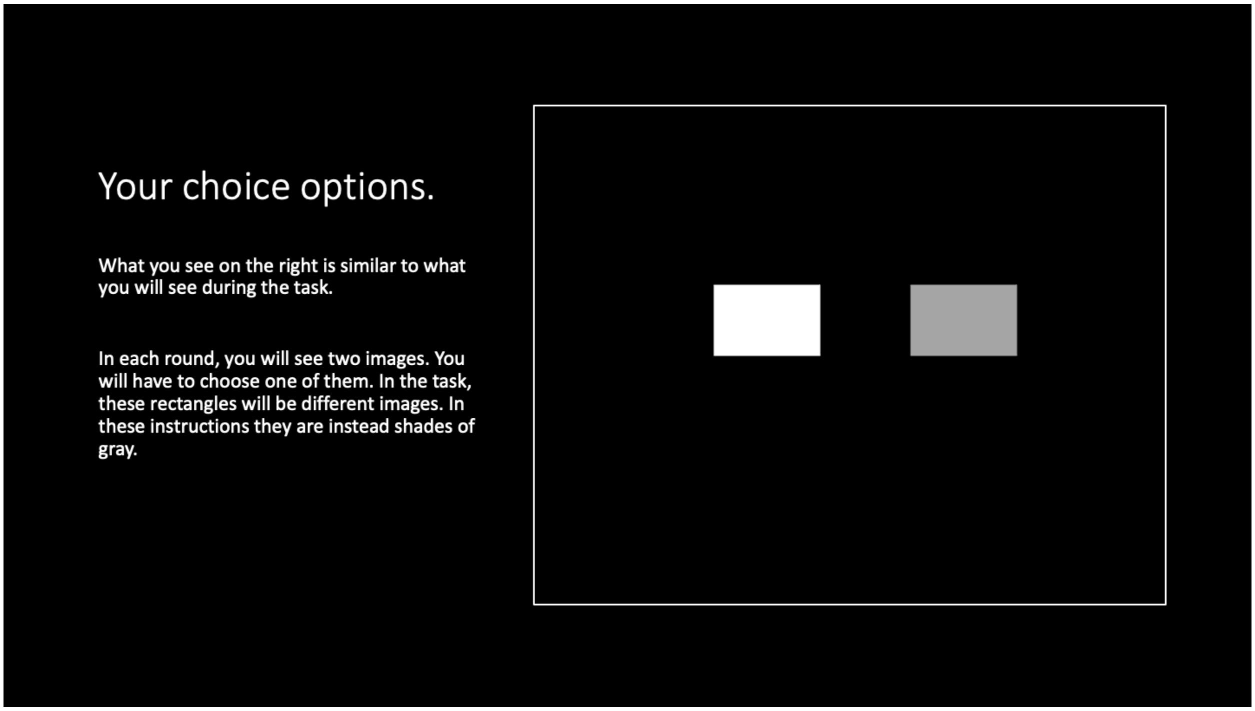
Your choice options.
What you see on the right is similar to what you will see during the task.
In each round, you will see two images. You will have to choose one of them. In the task, these rectangles will be different images. In these instructions they are instead shades of gray.

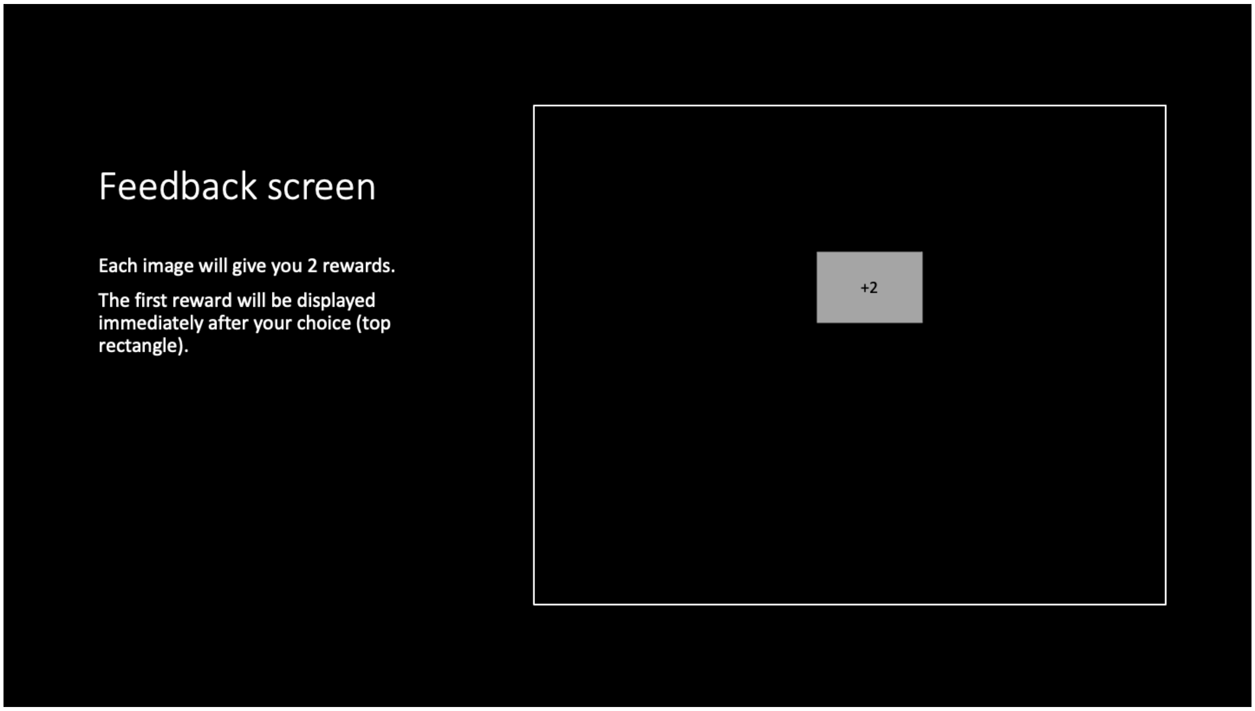
Feedback screen
Each image will give you 2 rewards.
The first reward will be displayed immediately after your choice (top rectangle).
+2

Feedback screen

The second reward will be displayed in the following round after your next choice (bottom rectangle).

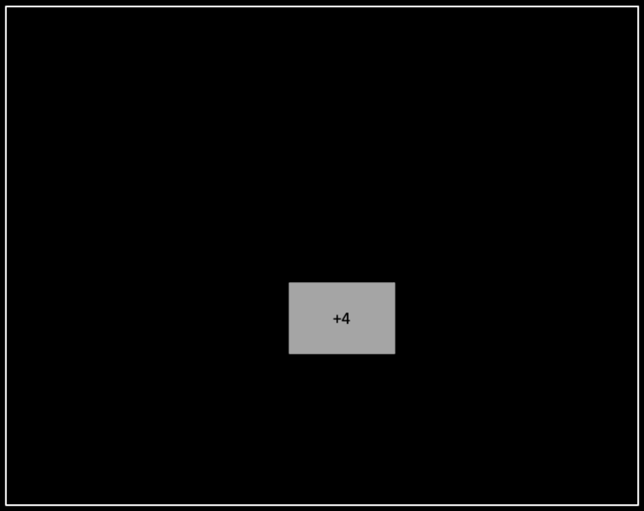

Feedback screen

The rewards depend on the image you choose and on chance. <u>Each image has a different average reward</u>, but there is some randomness so that each reward can vary by up to 3 points.

The more points you earn in the task, the higher your payment will be.

Feedback screen

This is what a feedback screen would really look like. The top rectangle (dark gray) is the one you just chose. On it you can see the first reward that this image has given you.

The bottom rectangle (light gray) is the one you chose in the previous round. On it you can see the second reward that this image has given you.

It's worth noting that the first and second reward from an image are usually different.

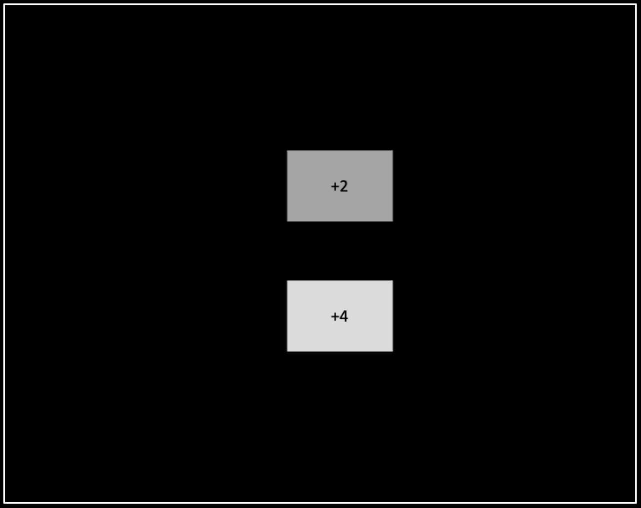

Making your choice

You make your choice by pressing the left arrow key (for the left image) or the right arrow key (for the right image).

If you do not decide in 3 seconds, the computer will randomly choose one image for you. Each time you fail to decide within 3 seconds, we will **subtract 5 points** from the total number of points you earned in the experiment.

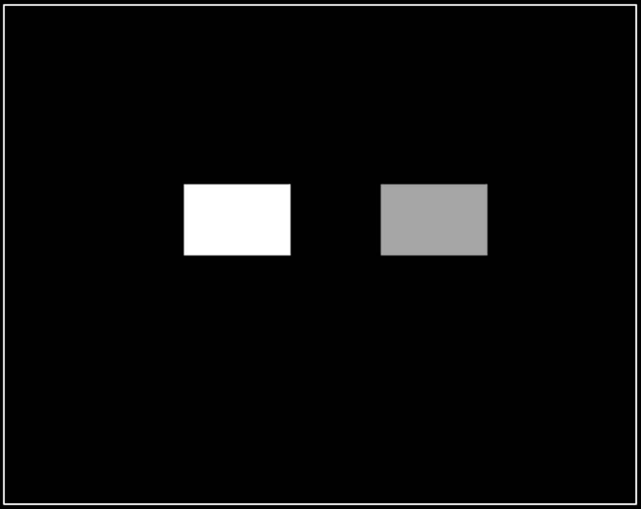

Your payment for the study will be calculated as follows:

- You will receive **4 cents** for each point you earn after you have achieved 1505 points.
- The first 1505 points are not rewarded.

- Example: you gather 1805 points – that is 300 points above 1505.
- You would therefore receive 300 x 0.04 = $12.00.

Summary

- You will choose between **2 images** in each round.
- Each image will bring **2 rewards**, in points. Points are converted to cash at the end.
- The first and second reward for an image are usually different.
- The average reward for each image is different.
- You have **3 seconds** to make each decision, by pressing the **left or right arrow keys**.

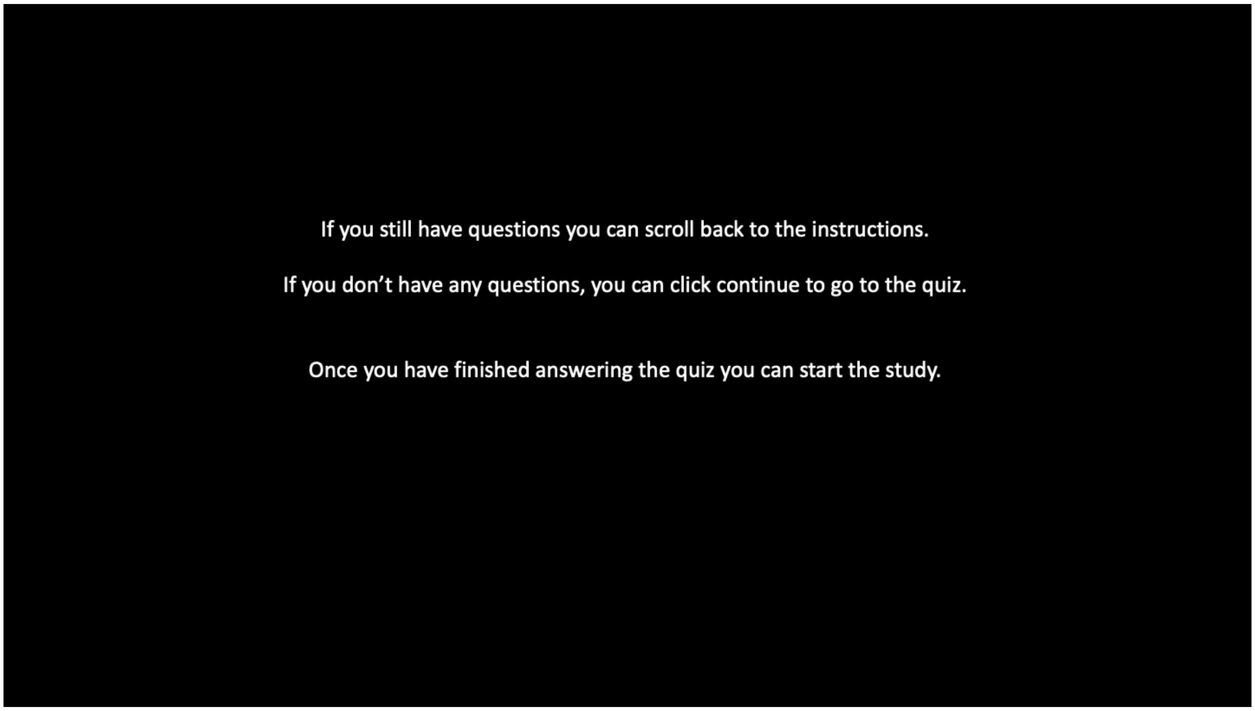
If you still have questions you can scroll back to the instructions.
If you don’t have any questions, you can click continue to go to the quiz.
Once you have finished answering the quiz you can start the study.

Consider the following example displaying choices and payoffs from two consecutive rounds of the task.

Round 31 – Feedback screen

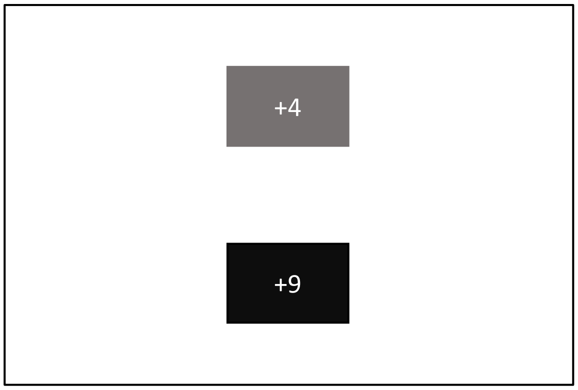

Round 32 – Feedback screen

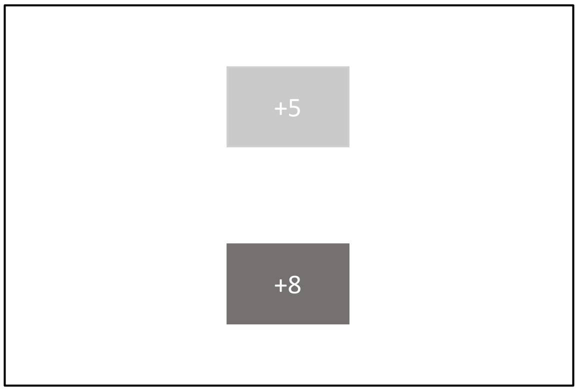

Question 1: What does the top number (+4) in Round 31 represent?

- **The first payoff for the dark gray square.**
- The payoff is not relevant to the task.
- The first payoff for the black square.
- The second payoff for the dark gray square.

Question 2: What does the bottom number (+9) in Round 31 represent?

- The payoff is not relevant to the task.
- The first payoff for the black square.
- **The second payoff for the black square.**
- The first payoff for the dark gray square.

Question 3: What is your total payoff for Round 31?

- 4
- 9
- 11
- **13**

Question 4: What is the total payoff of the dark gray square from Round 31 and Round 32?

- **12**
- 16
- 13

Question 5: What was chosen in Round 32?

- The dark gray square.
- **The light gray square.**

Question 1

In round 31, it can be inferred from the feedback screen that the dark gray square was chosen in that round and the white square was chosen in the previous round.

Recall that each image will give you 2 payoffs; one immediately after your choice (always the top image) and one in the next round (always the bottom image). Therefore, the number +4 on the top image (the dark gray square) represents the **first payoff for the dark gray square**.

Question 2

In round 31, it can be inferred from the feedback screen that the dark gray square was chosen in that round and the white square was chosen in the previous round.

Recall that each image will give you 2 payoffs; one immediately after your choice (always the top image) and one in the next round (always the bottom image). Therefore, the number +9 on the bottom image (the white square) represents the **second payoff for the white square**.

Question 3

Your total payoff in points for a round is the sum of the payoffs for that round. In this case, for round 31 you got:

- + 4 as the first payoff for the dark gray square chosen in that round.
- + 9 as the second payoff for the white square chosen in the previous round.

Your total payoff is therefore: 4 + 9 = **13.**

Question 4

Recall that each image will give you 2 payoffs; one immediately after your choice (always the top image) and one in the next round (always the bottom image). The dark gray square gives you + 4 in round 31 and + 8 in round 32. Therefore, the total payoff for the dark gray square is **12.**

Question 5

Recall that each image will give you 2 payoffs; one immediately after your choice (always the top image) and one in the next round (always the bottom image).

In round 32, it can be inferred from the feedback screen that the **light gray square** was chosen in that round and the dark gray square was chosen in the previous round.
